# LA-iMageS: a software for elemental distribution bioimaging using LA–ICP–MS data

**DOI:** 10.1186/s13321-016-0178-7

**Published:** 2016-11-18

**Authors:** Hugo López-Fernández, Gustavo de S. Pessôa, Marco A. Z. Arruda, José L. Capelo-Martínez, Florentino Fdez-Riverola, Daniel Glez-Peña, Miguel Reboiro-Jato

**Affiliations:** 1 ESEI: Escuela Superior de Ingeniería Informática, University of Vigo, Edificio Politécnico, Campus Universitario As Lagoas s/n, 32004 Ourense, Spain; 2 Group of Spectrometry, Sample Preparation and Mechanization (GEPAM), Institute of Chemistry, University of Campinas, UNICAMP, PO Box 6154, Campinas, SP 13084-62 Brazil; 3 National Institute of Science and Technology for Bioanalytics, Institute of Chemistry, University of Campinas, UNICAMP, Campinas, SP 13083-862 Brazil; 4 UCIBIO-REQUIMTE, Chemistry Department, Faculty of Science and Technology, University NOVA of Lisbon, 2829-516 Monte da Caparica, Portugal; 5 ProteoMass Scientific Society, Madan Parque, Rua dos Inventores, 2825-182 Caparica, Portugal

**Keywords:** Elemental distribution, Laser ablation, LA–ICP–MS imaging, Software

## Abstract

The spatial distribution of chemical elements in different types of samples is an important field in several research areas such as biology, paleontology or biomedicine, among others. Elemental distribution imaging by laser ablation inductively coupled plasma mass spectrometry (LA–ICP–MS) is an effective technique for qualitative and quantitative imaging due to its high spatial resolution and sensitivity. By applying this technique, vast amounts of raw data are generated to obtain high-quality images, essentially making the use of specific LA–ICP–MS imaging software that can process such data absolutely mandatory. Since existing solutions are usually commercial or hard-to-use for average users, this work introduces LA-iMageS, an open-source, free-to-use multiplatform application for fast and automatic generation of high-quality elemental distribution bioimages from LA–ICP–MS data in the PerkinElmer Elan XL format, whose results can be directly exported to external applications for further analysis. A key strength of LA-iMageS is its substantial added value for users, with particular regard to the customization of the elemental distribution bioimages, which allows, among other features, the ability to change color maps, increase image resolution or toggle between 2D and 3D visualizations.

**Electronic supplementary material:**

The online version of this article (doi:10.1186/s13321-016-0178-7) contains supplementary material, which is available to authorized users.

## Background

Advances in technology, including software, new generations of instruments, and the fastest electronic devices, allow the application of image-forming techniques that capture the complexity of a sample in a single image by considering a dynamic or static system. Such techniques include electron/ion microscopy, satellite imaging, tomography, NMR and, more recently, mass spectrometry based on molecular or elemental-based techniques [1–4]. Also, mass spectrometry imaging (MSI) increased its application in several science areas, since improvements in instrumentation, sample preparation and image software has been carried out [5]. In studies of biological systems, different classes of biomolecules were assessed with spatial resolution at the microscale. The main strategies of MSI involve matrix-assisted laser desorption/ionization mass spectrometry (MALDI–MS) as well as secondary ion mass spectrometry (SIMS).

In this context, laser ablation inductively coupled plasma mass spectrometry (LA–ICP–MS) has been widely used to qualitative or quantitative imaging [6–10]. In brief, LA–ICP–MS consists of a hyphenated technique where a laser unit is coupled to ICP–MS equipment. The laser unit is composed of an active medium (the most popular are Nd:YAlG lasers operated at 266 or 213 nm, or the ArF laser at 193 nm) which will produce a pulse with enough energy to ablate the sample, as well as other components, such as a resonator cavity and optical camera, among others [11, 12]. The ablated material is then transported to the ICP–MS through a gas (currently Ar, in addition to others), allowing an analysis of the sample. The source of ICP–MS produces ions, which are separated by their mass-to-charge ratio in the mass spectrometer. Ion intensities of each element are recorded against time during the laser scanning. These data are subsequently converted to pixels and an image is then built-up, enabling a spatial visualization of all phenomena occurring in the sample.

Coupling a laser unit with ICP equipment is now such a common task that various companies are commercializing this hyphenated technique. In fact, our research group has recently demonstrated in a tutorial review how to obtain bioimaging from elemental distribution by using MatLab software [13]. Nevertheless, such a strategy does not match the speed of data acquisition, since several hours are needed for the initial data acquisition process, while several days are required for their subsequent processing and image building. Although different solutions such as IMAGENA [14], Origin [15], PMOD [16], Maya vi2 [17], SMAK [18], or Microsoft Excel 2007 Macros [19], have been successfully used for generating images from LA–ICP–MS data, they still suffer from major drawbacks: (1) most of them are not freely available (e.g., IMAGENA, Origin, PMOD), (2) most of them are general-purpose programs requiring users to adapt data and learn specific skills (e.g., programming languages and coding knowledge), or (3) they are not specifically designed to run automated analysis workflows and require a lot of user intervention. In this scenario, there is a clear gap to develop a freely-available software that can automatically produce accurate images and make the LA–ICP–MS application more popular and friendly in its imaging mode.

This is precisely the direction of this study, which proposes the LA-iMageS software to easily process all the data generated by LA–ICP–MS in the PerkinElmer Elan XL format, ensuring a fast processing workflow (data processing is done in few seconds after inserting the data) as well as the generation of elemental distribution images (with a quality comparable to those obtained through MatLab). To demonstrate the usefulness of our LA-iMageS application, a diversity of systems/samples were used as examples, such as *Arabidopsis thaliana* seeds and histological slides from human tissues, working in two or three dimensions. In addition to the multitude of applications, the LA-iMageS proposal produces a link with different fields of scientific research (e.g., metallomics, medicine, biology, environmental and geology, among others), enabling a suitable space for transdisciplinary collaborations.

## Methods

**Fig. 1:**
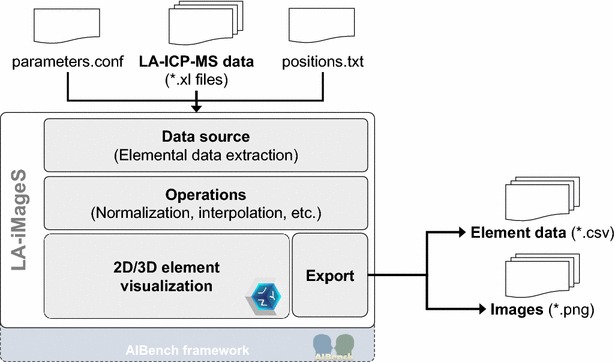
LA-iMageS software architecture

The LA-iMageS program is a graphical application that automates the data processing and elemental distribution visualization of LA–ICP–MS bioimaging. The overall architecture of LA-iMageS can be seen in Fig. 1.

### Input data

LA-iMageS uses datasets in PerkinElmer Elan XL format (*.xl) as input, which is commonly generated by ICP–MS instrument control software from PerkinElmer, such as Elan 6 × 00 or Elan DRC-e. Each input dataset should be placed in a folder containing the XL files corresponding to each data line taken by the ICP–MS instrument. Each line file must contain a number that indicates the order in which it has been acquired by the ICP–MS instrument. For instance, in a dataset with ten lines, a valid set of names can be: *line 1.xl*, *line 2.xl*, *line 3.xl*, *line 4.xl*, *line 5.xl*, *line 6.xl*, *line 7.xl*, *line 8.xl*, *line 9.xl*, and *line 10.xl*. Additionally, the dataset folder may include two optional files: *parameters.conf*, containing the ICP–MS data acquisition parameters, and *positions.txt*, containing the physical position of each line.

### Data acquisition parameters

The optional *parameters.conf* file is used by the LA-iMageS software to automatically load acquisition parameters. If this file is not present in the dataset’s directory, the user must manually introduce them. However, experience demonstrates that it is a good practice to save the acquisition parameters along with data line files.

The data acquisition parameters that can be specified in this file are the following:

- *Standard* the standard element in the dataset. Internal standard is an element used to normalize the results and to overcome instrumental oscillation. The other elements will be normalized using this element as standard. Users must choose a specific element to be monitored, for example, a known matrix element in the sample or those intentionally added by the user. The chosen element should minimize the variability of the ablation process, which can be caused by local differences in tissue thickness and/or different interaction between the laser and the sample surface, allowing the observed signal to correspond to an elemental concentration in a specific location.
- *Ablation speed* speed set by the user in the laser operational mode as *continuous firing*. This parameter relates to the spot size, which is determined by the laser beam diameter, and by the frequency, which corresponds to the repetition rate of the laser. Usually, the value of ablation speed used is lower than spot size.
- *Acquisition time* refers to the time needed for the acquisition of one point considering all the elements monitored by the ICP–MS. This parameter is intrinsically correlated to ICP–MS parameters, such as the number of isotopes monitored, sweeps, number of replicates and dwell (or residence) time. The acquisition time should not be higher than 1.0 s, since the elemental distribution information would be lost.
- *Space interval* represents the distance among the center of two lines. The lowest space interval results in the highest image resolution.

### Data lines positions

The *positions.txt* file can be used to specify the physical position of each line in the instrument during the ablation process. This information is important for the elemental data extraction process explained below, since data acquisition can be made with laser position in the horizontal or vertical profile.

This file is optional and is used by the LA-iMageS software to read the position of each line and determine whether they are horizontal or vertical: if each line has the same X position while the initial and final Y-positions are different, it means that it is vertical; otherwise, if each line has the same Y-position while the initial and final X-positions are different, it means that it is horizontal. If this file is not present in the dataset’s directory, then LA-iMageS will consider the lines to be horizontal and will automatically generate their positions based on the acquisition parameters.

Since this file can be easily generated by ICP–MS instrument control software, we strongly encourage keeping a positions file along with the data lines files.

### Elemental data extraction

Elemental data is extracted from the input dataset in XL format, using the two optional configuration files if necessary. LA-iMageS parses input data in order to obtain one two-dimensional matrix per element in the dataset, which stores the analyte distribution in the sample. Acquisition parameters are used along each positions file for axis definition.

**Fig. 2:**
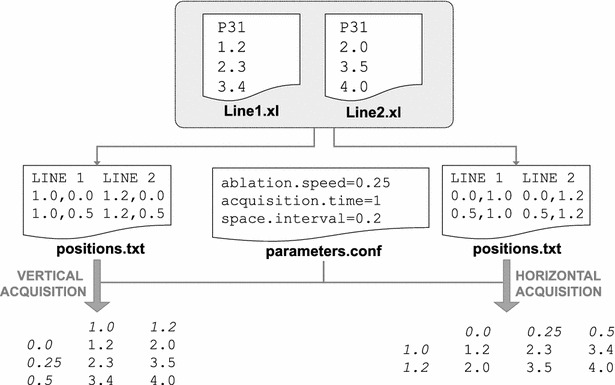
Elemental data extraction process. In this example, ^31^P^+^ distribution is extracted from two line files using two positions files, one that defines a *vertical* orientation and another that defines a *horizontal* orientation

For instance, when data is acquired using the laser in the horizontal position, acquisition time and ablation speed parameters are used for *x*-*axis* definition: line measurements are separated by intervals of acquisition time multiplied by ablation speed. Conversely, the *y*-*axis* is simply defined by the spacing among the lines (i.e., space interval parameter). When data is acquired using the laser in the vertical position, the *x*-*axis* and *y*-*axis* are opposite to those in the horizontal mode, as Fig. 2 illustrates.

After the elemental data extraction process, each element is normalized by the specified standard element, dividing its intensity matrix by that of the standard.

### Data visualization

**Fig. 3:**
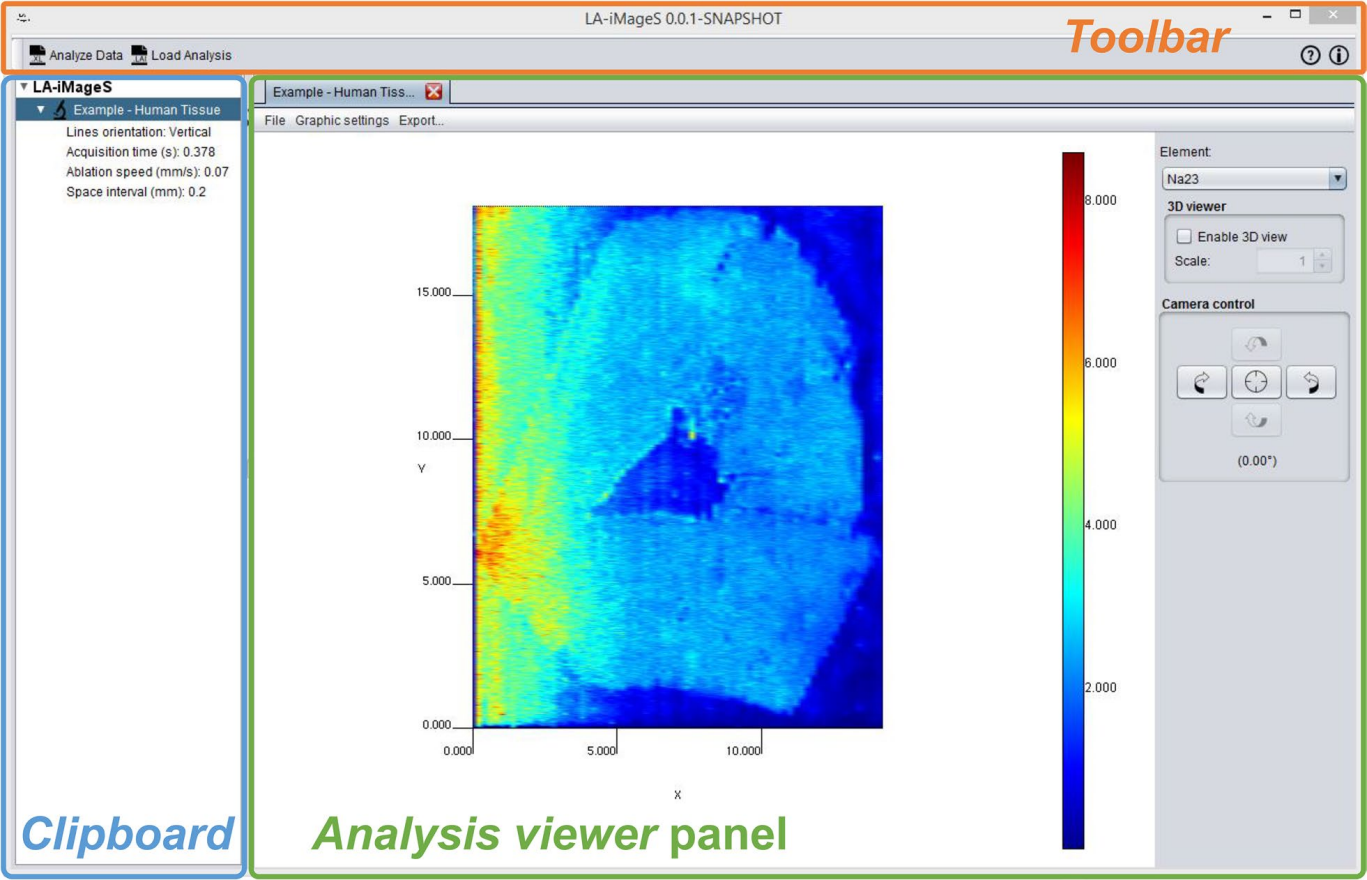
LA-iMageS graphical user interface (GUI) showing ^23^Na^+^ distribution from a human tissue sample (Additional file 1)

The main user interface of LA-iMageS (Fig. 3) is organized into three main sections: the *Toolbar*, the *Clipboard*, and the *Analysis viewer*. Through the toolbar, users can access the main functions of LA-iMageS, where ‘data analysis’ is the most important operation. On the clipboard tree, users can find a list of loaded datasets. Finally, users can explore elemental data through the *Analysis viewer* panel.

The most important section is the *Analysis viewer* panel, which consists of a 2D/3D representation of the current element distribution with a menu bar and a right sidebar providing access to several configuration options. It is important to stress out that 3D visualization reflects the signal intensity of each analyte only, and not the real topology of the sample.

The right sidebar allows users to select the element distribution that is being currently displayed, enable or disable the 3D view, and control the camera position.

The menu bar contains three submenus: (1) *File*, which allows saving the current analysis; (2) *Graphical Settings*, which enables the customization of the elemental distribution image; and (3) *Export*, which provides exporting facilities.

### Tuning up the elemental distribution image

**Fig. 4:**
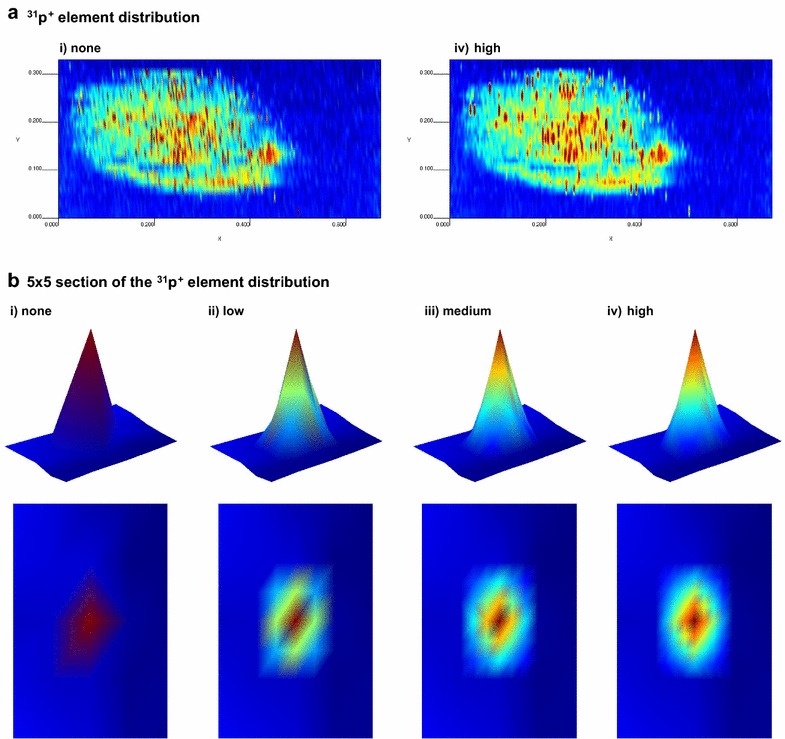
Effect of interpolations. **a** ^31^P^+^ elemental distribution image in *Arabidopsis thaliana* seed (Additional file 2) using different interpolation levels: *i* no interpolation and *iv* high. **b** Detail of a 5 × 5 section of the ^31^P^+^ elemental distribution (Additional file 3) using different interpolation levels: *i* no interpolation, *ii* low, *iii* medium and *iv* high

Elemental distribution images can be tuned up throughout the *Graphical Settings* submenu. A very useful feature of this submenu is the interpolation level, since it allows creating new data points by interpolation within the range of the original set of data points, thus increasing image quality. Figure 4 illustrates how interpolation can help to improve image resolution.

**Fig. 5:**
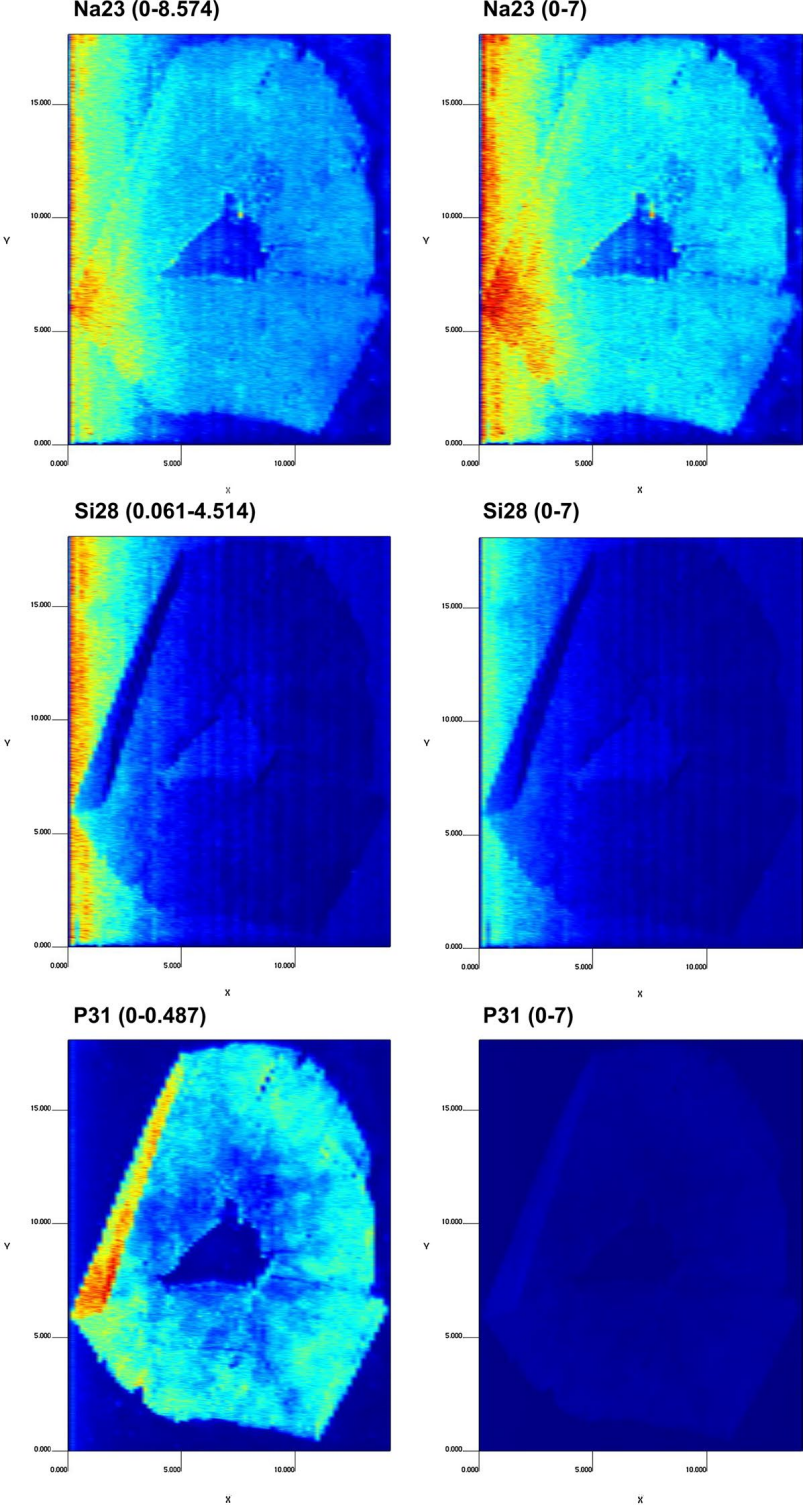
Effect of the range of values used for the *color map* for three elements from histological slides of a human sample (Additional file 1). Images on the *left* correspond to *color maps* generated using the corresponding element distribution’s range of values. Images on the *right* correspond to *color maps* generated by using the same, fixed, range of values (0–7)

Another important aspect to obtain good images is the color map adjustment. LA-iMageS allows customizing the color palette used to represent the image, as well as the range of values of the color map. Since each element has its own intensity range, this latter option is especially useful to obtain comparable images of different elemental distributions by setting a color map within the same range of values for each element (Fig. 5).

### Data export

After exploring data in the LA-iMageS, it is expected that users can employ their results in other complementary applications such as a word processor or another analysis package. To fulfill these needs, both elemental distributions (i.e., the data matrices) and images can be easily saved throughout the *Export* submenu of the *Analysis viewer* panel. While elemental distributions are exported using comma-separated values (CSV) files, 2D/3D bioimages are exported into portable network graphics (PNG) files.

When exporting data, users can choose to save only the element shown in the *Analysis viewer* panel, or all the elements of the dataset in a row with the same export configuration.

### Implementation

The LA-iMageS software is implemented in Java using the AIBench framework [20]. It is provided as a self-contained, multiplatform Java standalone application. LA-iMageS is an open source project hosted on Github. The code architecture is interface driven, so developers can easily integrate new data formats and/or functions. The jzy3D and the Apache Commons Math Java libraries are also integrated within the project to render 2D/3D images and to perform mathematical operations (e.g., bilinear interpolation).

## Results and discussion

With the goal of demonstrating the usefulness and features of LA-iMageS, this section presents a case study showing the ^31^P^+^ and ^63^Cu^+^ distribution in *Arabidopsis thaliana* seed. For a better comprehension of the use of the software, all steps regarding image edition are properly addressed in Additional file 4, and the dataset used in the case study can be found in Additional file 2.

### Case study dataset

A correct image analysis depends on a good balance between laser speed and ICP–MS acquisition. As we have discussed in our previous work [13], the quality of the images can be improved with optimization of several LA and ICP–MS parameters. Data are usually obtained by scanning the sample surface as parallel lines in order to show the elemental distribution in the sample, and each line ablated is then recorded in different files. Several file formats can be used by the manufacturer to acquire data. As previously explained, LA-iMageS accepts ICP–MS data in the PerkinElmer Elan XL format, where data are organized as rows and columns. Each row represents one intensity value at a time interval determined by parameters that influence the acquisition time (e.g., residence time, number of replicates, sweeps or readings, among others) of the ICP–MS, considering all *m/z* measured. The first column indicates the time, while subsequent columns show the results for each ion monitored. Using LA-iMageS, the user needs to indicate the local path where data were saved, and the acquisition parameters to automatically obtain the corresponding image.

In our example, the speed ablation was 10 µm s^−1^ and the ICP–MS acquisition time for each point was settled at 0.270 s. For providing the y-coordinate resolution, the distance among the lines was 15 µm for image building. All LA–ICP–MS instrumental conditions are shown in Table 1. The acquisition of two-dimensional images was performed in accordance to the method previously proposed [9, 13].

**Table 1:** Instrumental operational conditions and measurement by LA–ICP–MS

*Instrument settings*	
Nebulizer	Meinhard
Spray chamber	Cyclonic
RF power (W)	1300
Nebulizer gas flow (L min^−1^)	1.0
Auxiliary gas flow (L min^−1^)	2.0
*Data acquisition parameters*	
Reading mode	Peak hopping
Detector mode	Pulse
Sweeps	3
Dwell time (ms)	30
Integration time (ms)	270 (for each point)
Detector dead time (ns)	60
Lens voltage (V)	Automatic mode
Monitored isotopes	^12^C, ^63^Cu and ^31^P
*Laser conditions*	
Wavelength of Nd:YAG laser (nm)	213
Laser ablation intensity (%)	50
Frequency (Hz)	20
Spot size (µm)	12
Scan speed (µm s^−1^)	10
Resolution—X axis (µm)	2.7
Resolution—Y axis (µm)	15

Thus, the XL files obtained from the ICP–MS acquisition were copied from an instrument computer controller (Additional file 2). Each line generated through the ablation process resulted in one different file, and 23 lines were needed for mapping the entire sample surface, generating 23 XL files (considering the distance among the lines, as previously indicated).

### Elemental distribution of the Arabidopsis thaliana seed

In order to facilitate comprehension of the case study, a summary is presented in Additional file 4 (slide 1), showing all steps involved in the image building process. First, a view of the initial software panel is presented (slide 2), where the “Analyze Data” option is used to start the image building. This option shows a dialog box (slide 3), allowing users to select the folder containing the input XL files (slides 4 and 5) and introduce the ablation parameters (standard, ablation speed, acquisition time, and space interval). It is important to remember that these parameters could be stored in the *parameter.conf* file along with input XL files, to avoid the need for users to manually introduce them. A first view of the image is readily generated using default parameters, with additional edition steps required to obtain a good representation (slide 6).

To obtain an optimal image, the element intensity must be adjusted (slides 7–9) in accordance to the ratio between the analyte ^31^P^+^ and standard ^12^C^+^. This is achieved by customizing both the color map range and the color map palette. The color map range (slide 7) can be edited by clicking the sequence “Graphic settings” → “Range mode” → “Custom”, which enables a dialog (slide 8) to set the minimum and maximum color data levels (in this case study, 0.007 and 0.025, respectively). As illustrated in Fig. 5, each element can have a particular range of intensities, and the user must seek the best relation for obtaining the desired output.

Another way to improve the image is smoothing or interpolation, which results in better image resolution. By default, no interpolation is applied to the images but LA-iMageS allows users to apply three different levels: low, medium, and high. To improve the resolution of the image in this case study, the highest level is selected by clicking the sequence “Graphic settings” → “Interpolation level” → ”High” (slides 10 and 11).

**Fig. 6:**
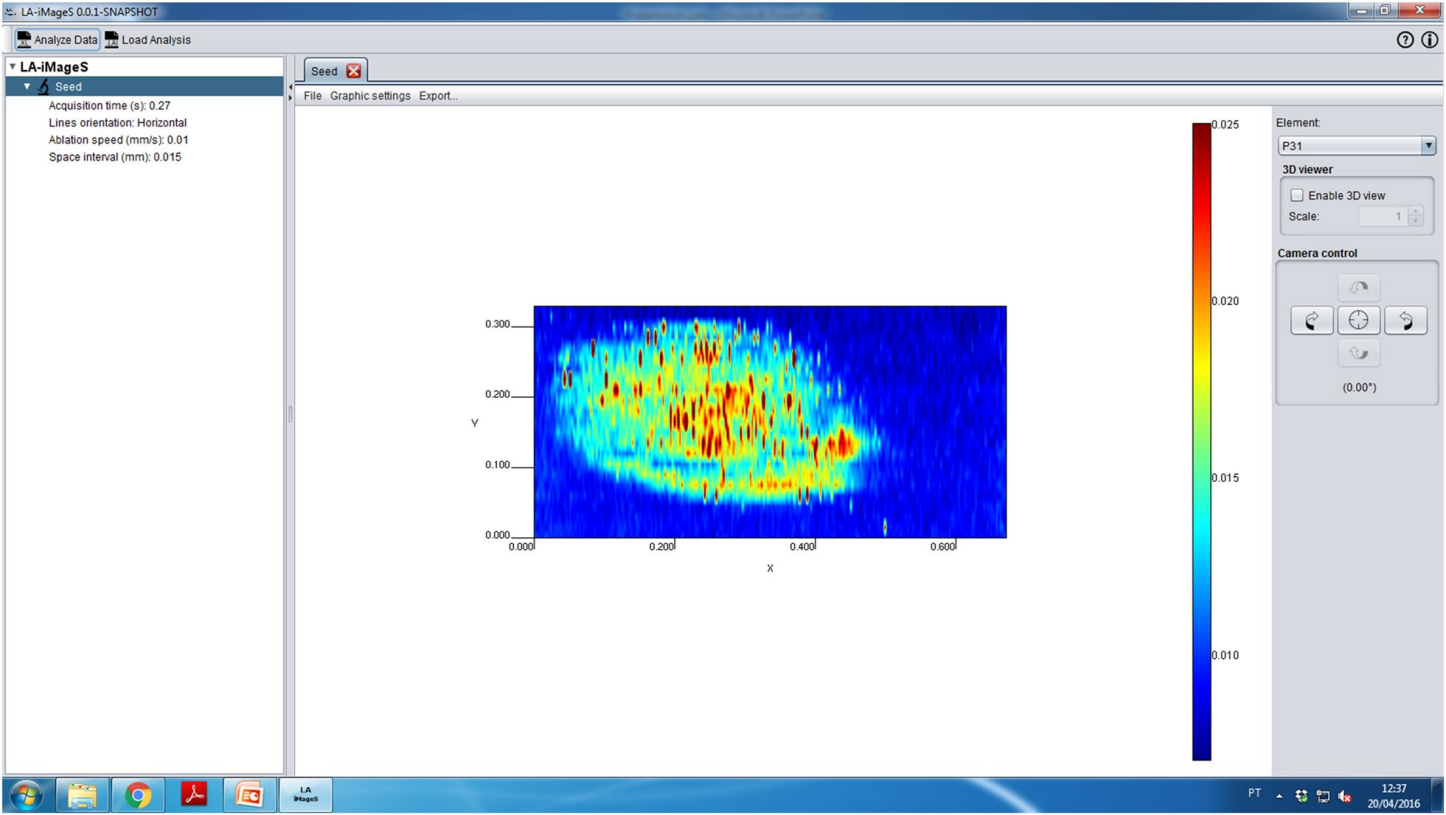
Screenshot of the LA-iMageS application showing the analyte ^31^P^+^ distribution after *color map* customization and interpolation

After carrying out these important editing steps (Fig. 6), the image is ready to be exported as a PNG file for further use in external applications (slides 12–15). By using the “Export” → “As image” menu options (slide 12), a new dialog box appears (slide 13) allowing users to set the size of the image in pixels. By clicking the “OK” button, the image is stored in the selected directory (slide 14), and can now be easily visualized or edited using any computer with Windows, Linux, or Mac operating systems (slide 15).

Alternatively, elemental distributions can be also saved as CSV files (slides 16–20) so that they can be further used as input in general-purpose scientific applications such as Matlab or Excel. Users can export the intensity ratio values between analyte and standard into a CSV file by using the “Export” → ”As CSV” menu options (slide 16), which opens a dialog to select a predefined CSV format (Excel compatible CSV, used in this case study, or Libre/Open Office compatible CSV) or by defining a custom format (slide 17). By clicking the “OK” button, the data is saved in the selected directory (slides 18 and 19). Finally, this CSV file is opened with Excel (slide 20).

A particularly useful feature of the LA-iMageS software is the possibility of saving the image configuration, so that it can be edited later or even reused in future experiments (slide 21–30). This can be done by using the “File” → “Save analysis” menu options, which opens a new dialog to select the folder and file with .lai extension (slides 22 and 23). This way, if LA-iMageS software is closed, the image edition can be retaken later at the same status. To recover the image (slides 25–30), users must use the “Load analysis” option of the toolbar (slide 25) and select the previously saved file (*Seed.lai* in our case study).

Finally, LA-iMageS provides additional features allowing a high degree of image customization. These features, illustrated in Additional file 4 (slides 32–55), include: (1) image rotation (slides 32–34), (2) three-dimensional elemental distribution visualization (slides 35–37), (3) axis hiding (slides 38–39), (4) restart image settings to the original conditions (slides 40–41), (5) element selection (slides 42–47), (6) color bar hiding (slides 48 to 51), and (7) axis tick lines hiding (slides 52–55).

## Conclusions

This work has presented LA-iMageS as a new open-source software for rapid processing and visualization of LA–ICP–MS data. Our application fully automates the process of generating elemental distribution images from LA–ICP–MS data. LA-iMageS is completely free and provides a friendly graphical user interface designed to avoid the need for a bioinformatics expert to use it.

Finally, LA-iMageS is open to further extension, such as supporting new data formats, including new operations, or improving those currently available.

## Availability and requirements

- Project name: LA-iMageS.
- Project home page: http://www.la-images.net
- Project source code repository: http://github.com/sing-group/la-images
- Operating system(s): Platform independent.
- Programming language: Java.
- License: GNU GPL v3.
- Any restrictions to use by non-academics: None.

For proper use, guidance and maintenance, please contact laimages@sing.ei.uvigo.es.

## Additional files

**Additional file 1.** Dataset corresponding to LA–ICP–MS analysis of a human tissue used to illustrate LA-iMageS features.

**Additional file 2.** Case study dataset corresponding to LA–ICP–MS analysis of a *Arabidopsis thaliana* seed.

**Additional file 3.** Dataset corresponding to a 5 × 5 section of ^31^P^+^ element from case study dataset (Additional file 2) to reproduce Fig. 4b

**Additional file 4.** Tutorial describing the steps to reproduce the case study and perform a full LA-iMageS analysis.
