# Supplementary figures and images for: LA-iMageS: a software for elemental distribution bioimaging using LA–ICP–MS data
Source: J Cheminform. 2016 Nov 18;8:65. doi: 10.1186/s13321-016-0178-7 (PMC5116144; doi:10.1186/s13321-016-0178-7)

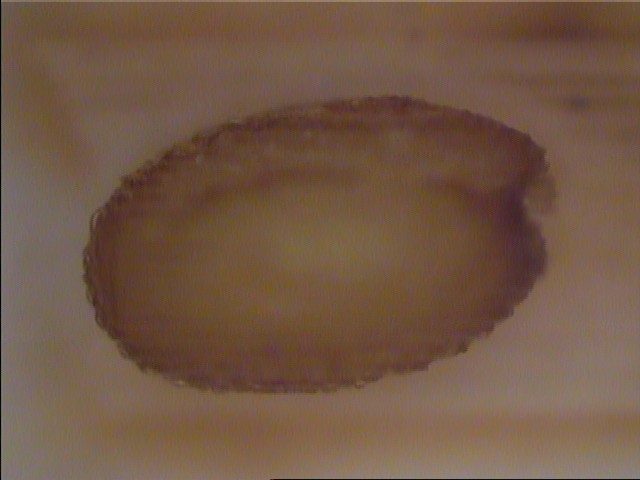

Supplement: Supplementary file 2 — Additional file 2. Case study dataset corresponding to LA–ICP–MS analysis of a Arabidopsis thaliana seed. [file 13321_2016_178_MOESM2_ESM.zip › seed0203.JPG]
