# Supplementary material for: LA-iMageS: a software for elemental distribution bioimaging using LA–ICP–MS data
Source: J Cheminform. 2016 Nov 18;8:65. doi: 10.1186/s13321-016-0178-7 (PMC5116144; doi:10.1186/s13321-016-0178-7)
Supplement: Supplementary file 4 — Additional file 4. Tutorial describing the steps to reproduce the case study and perform a full LA-iMageS analysis. [file 13321_2016_178_MOESM4_ESM.pptx]

## Slide 1
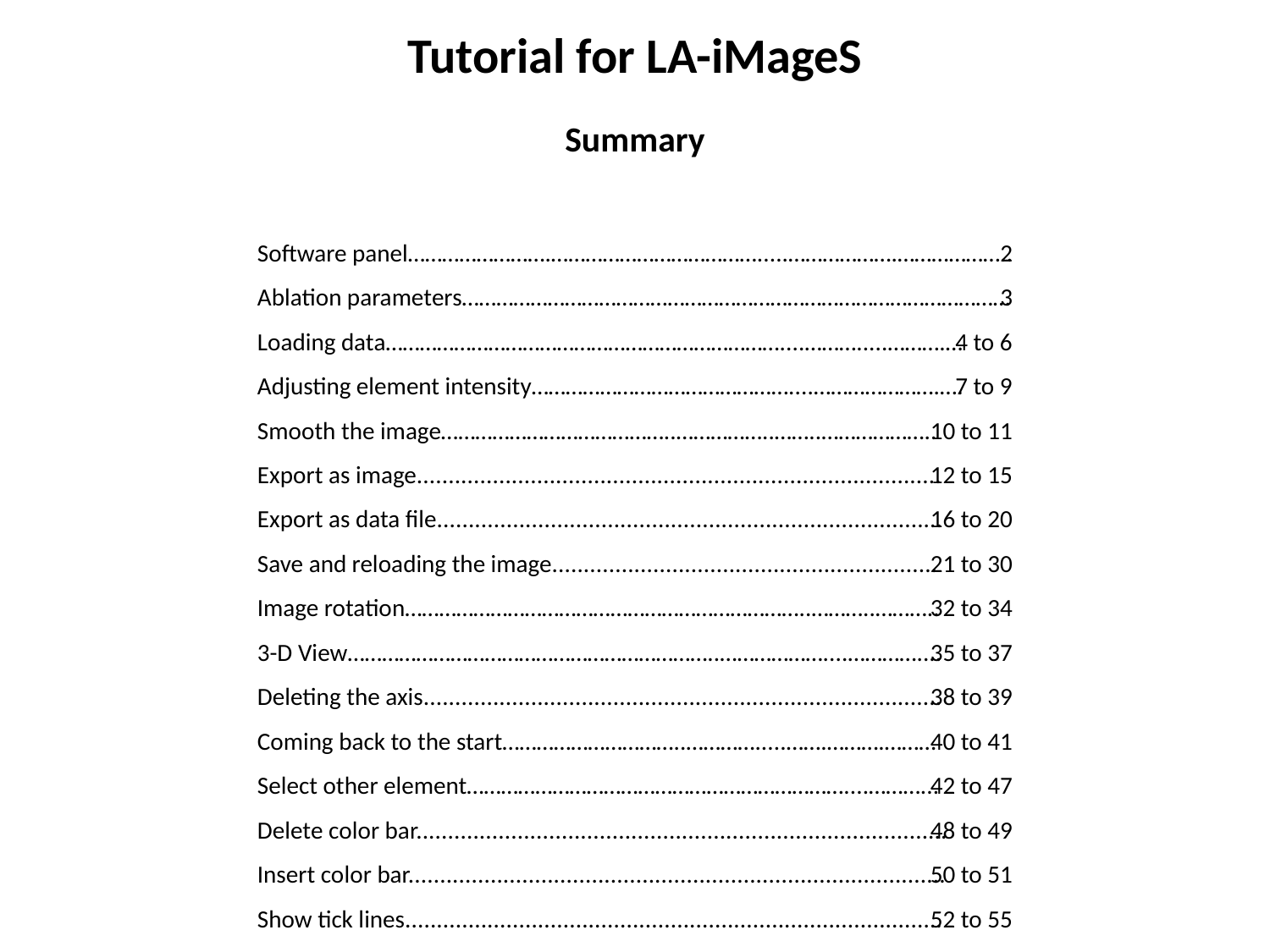

# Tutorial for LA-iMageS Summary
Software panel…………………….……………………………….....……………….………………..
Ablation parameters……………………………………………………………………………………
Loading data……………………………………………………………....……….....……….…
Adjusting element intensity………………………………………....………………….….
Smooth the image…………………………………..……………..……..………………..
Export as image...................................................................................
Export as data file................................................................................
Save and reloading the image.............................................................
Image rotation……………………………………………………………..………..………..
3-D View………………………………………………………..………………....…………....
Deleting the axis..................................................................................
Coming back to the start…………………………..………….....…….……….………
Select other element…………………………………………………………....………...
Delete color bar....................................................................................
Insert color bar.....................................................................................
Show tick lines.....................................................................................
2
3
4 to 6
7 to 9
10 to 11
12 to 15
16 to 20
21 to 30
32 to 34
35 to 37
38 to 39
40 to 41
42 to 47
48 to 49
50 to 51
52 to 55

## Slide 2
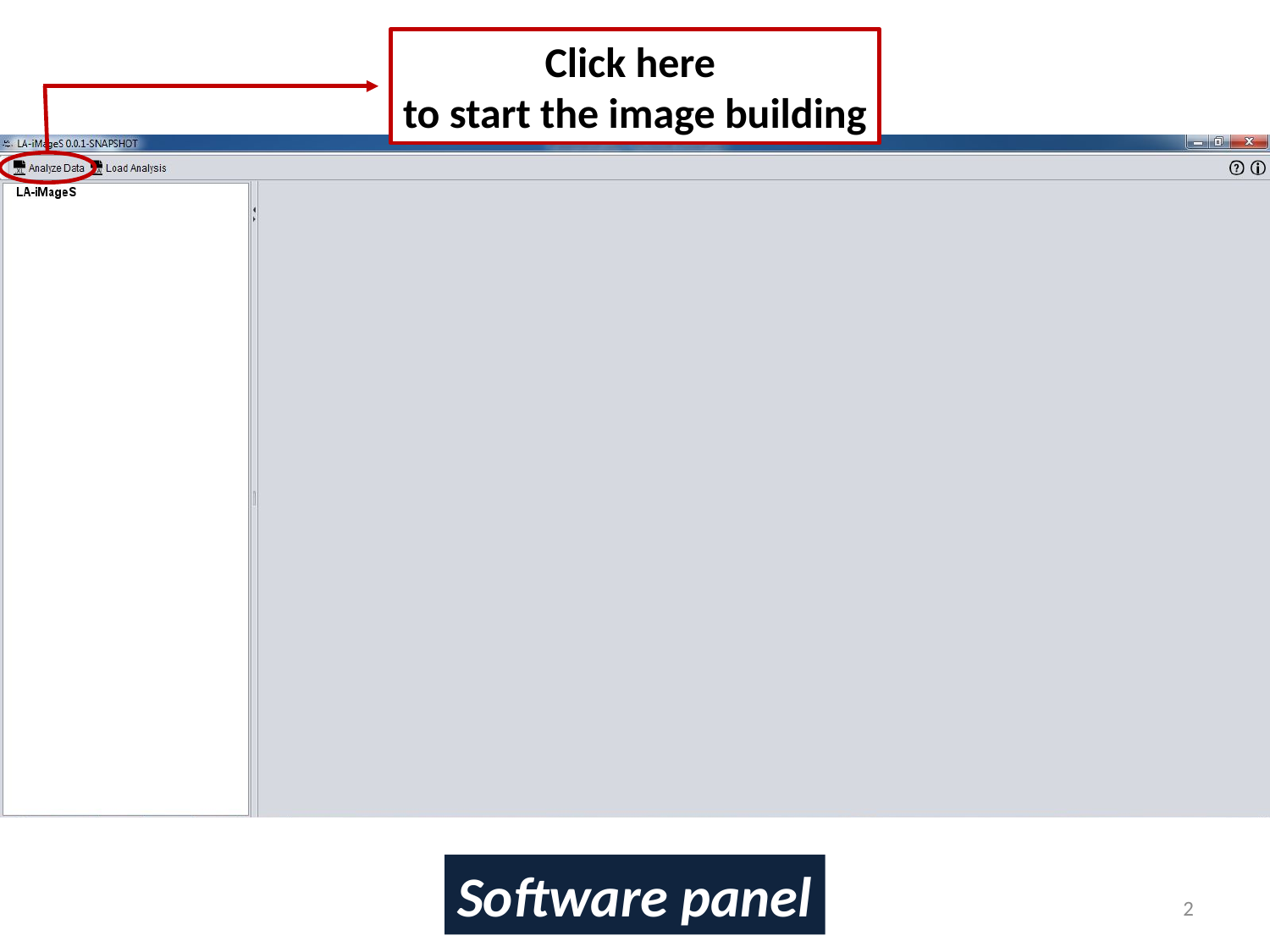

Click here
to start the image building
Software panel
2

## Slide 3
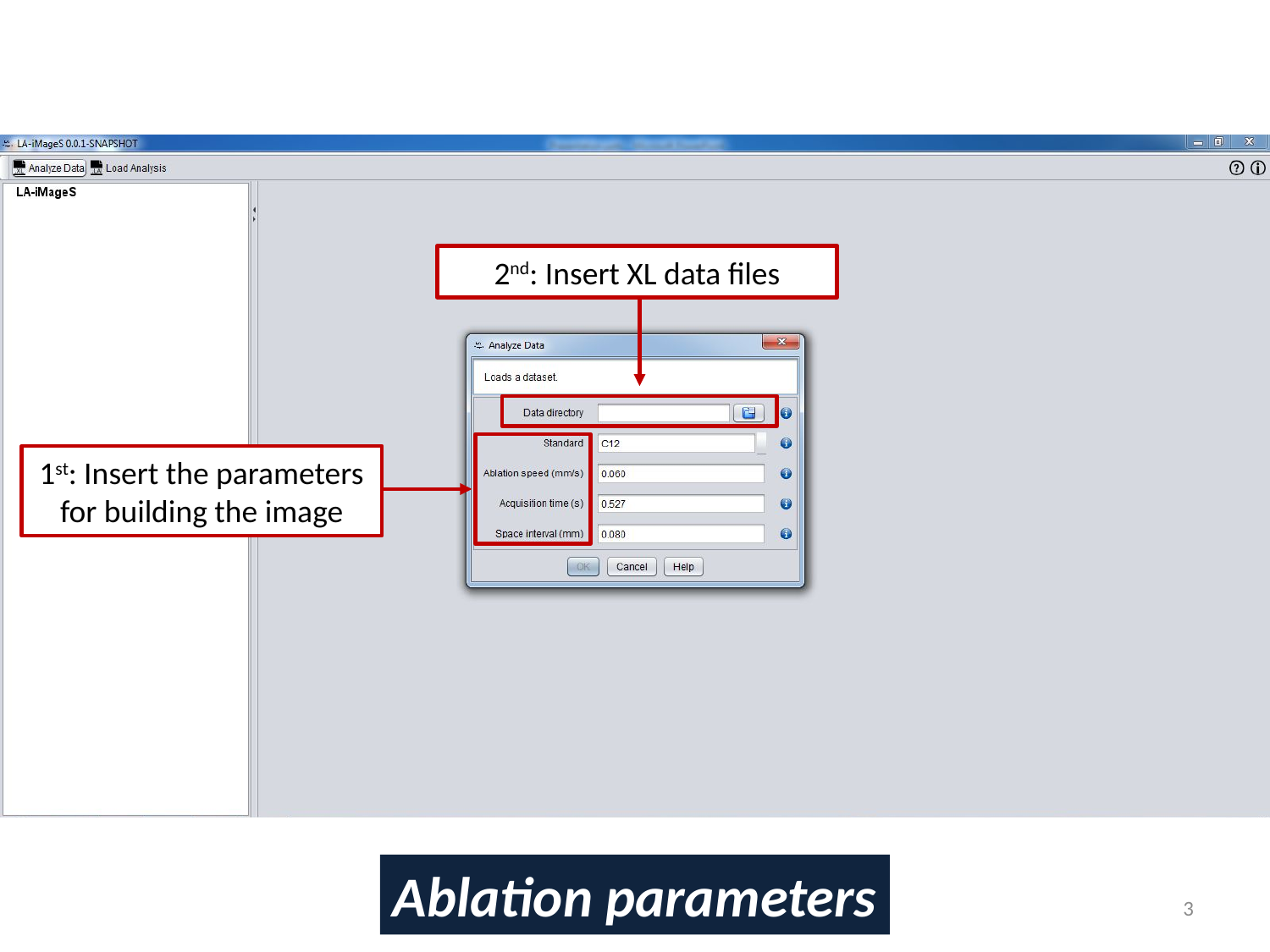

2nd: Insert XL data files
1st: Insert the parameters
for building the image
Ablation parameters
3

## Slide 4
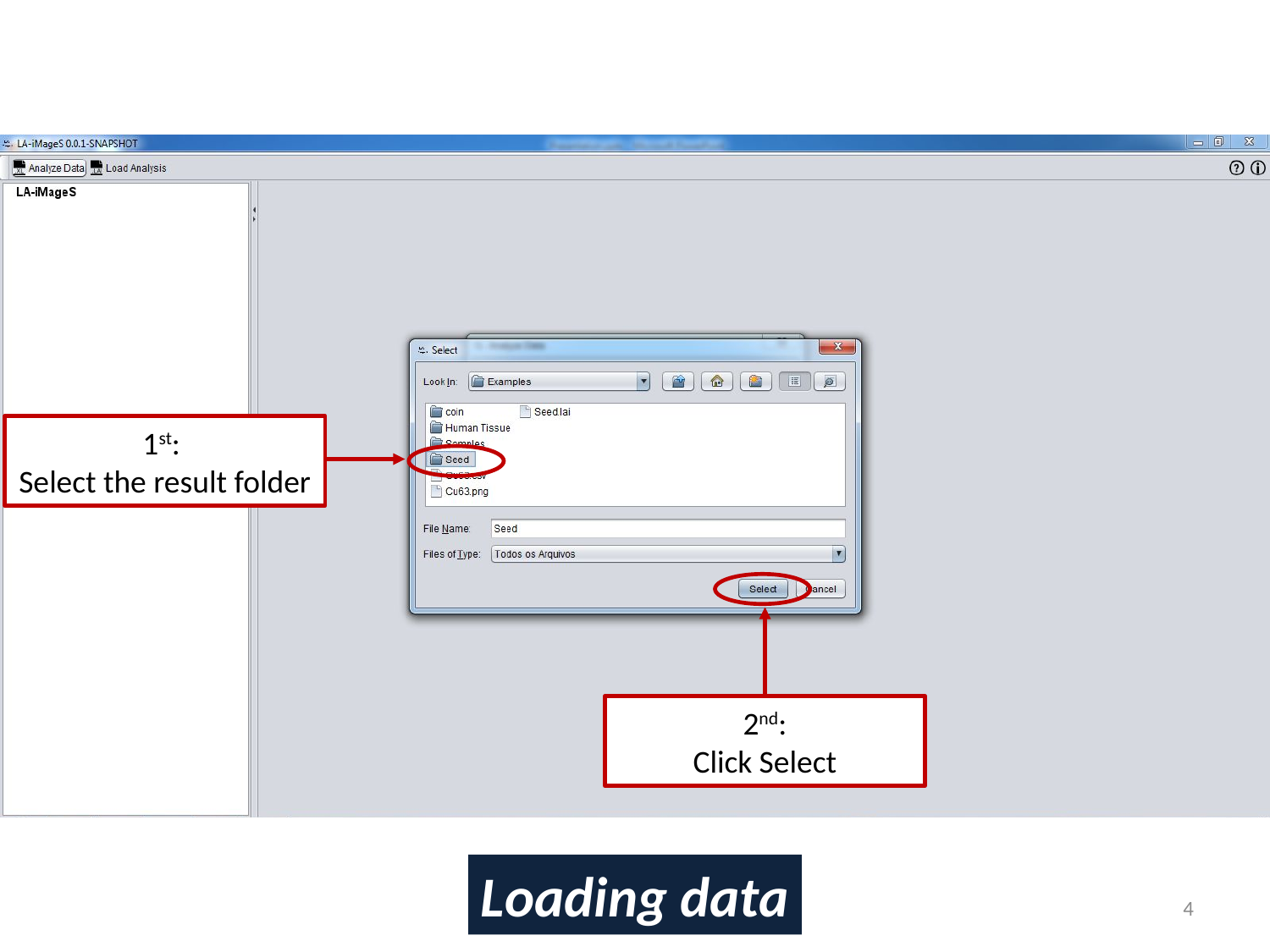

1st:
Select the result folder
2nd:
Click Select
Loading data
4

## Slide 5
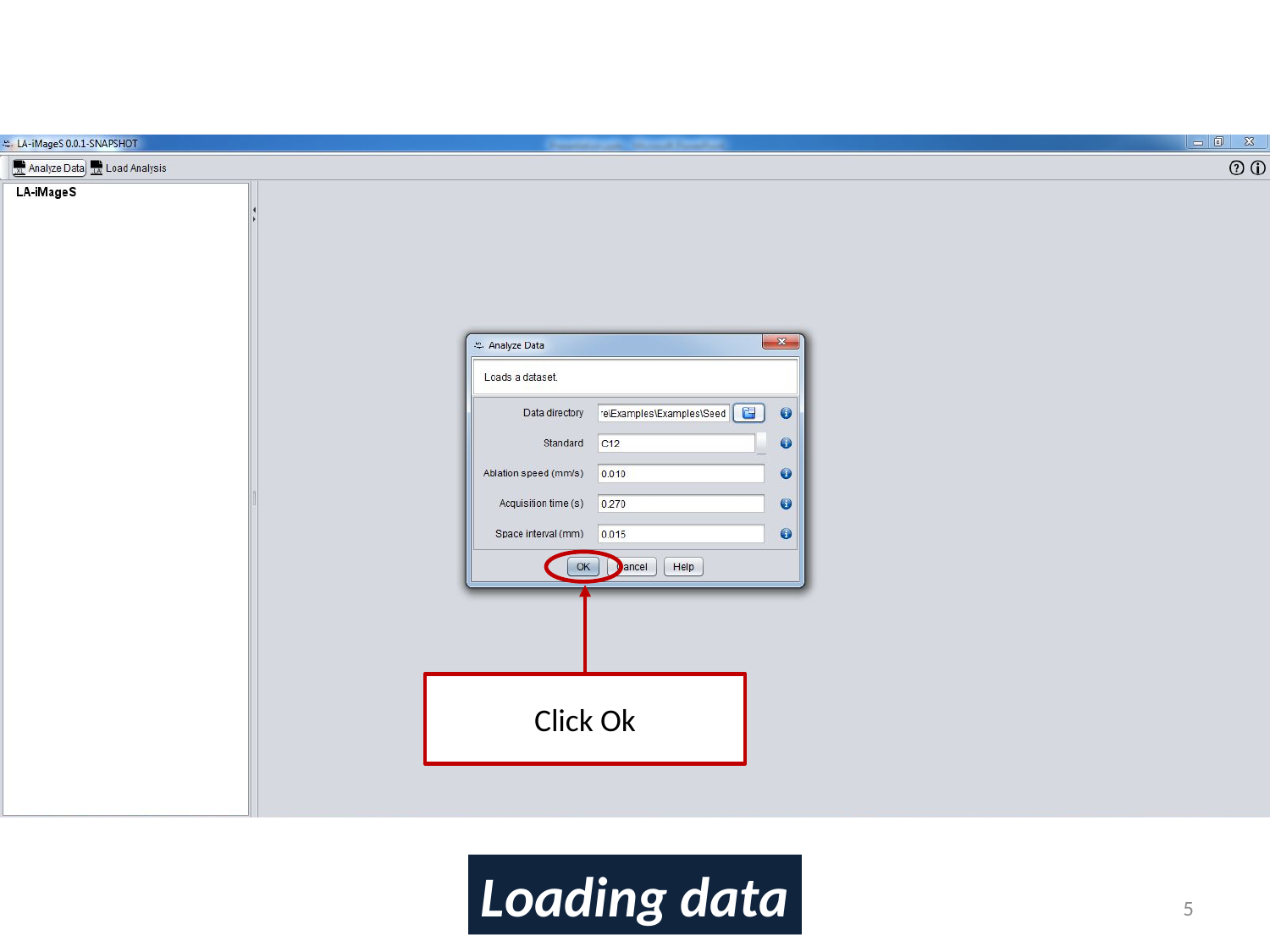

Click Ok
Loading data
5

## Slide 6
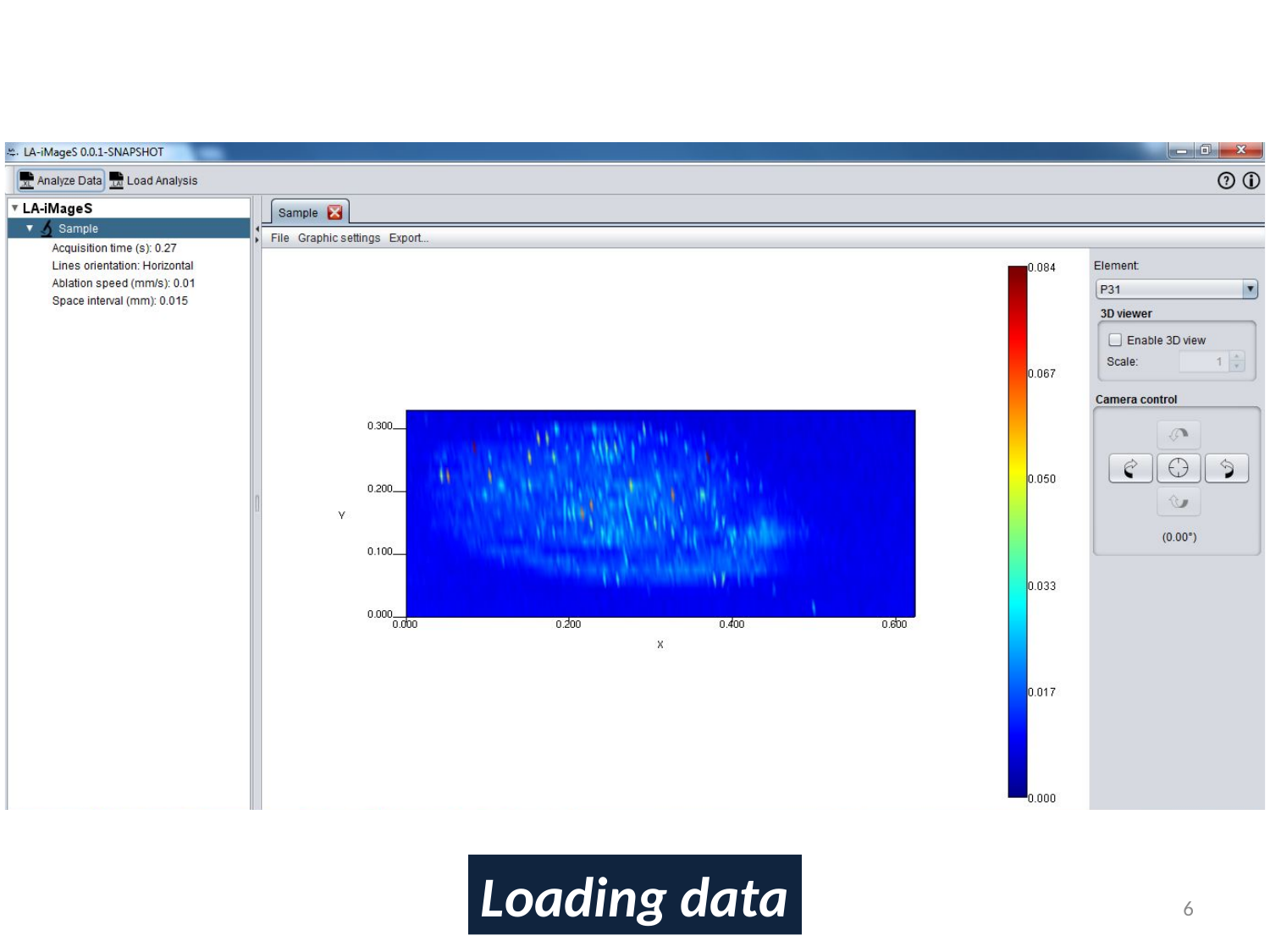

Loading data
6

## Slide 7
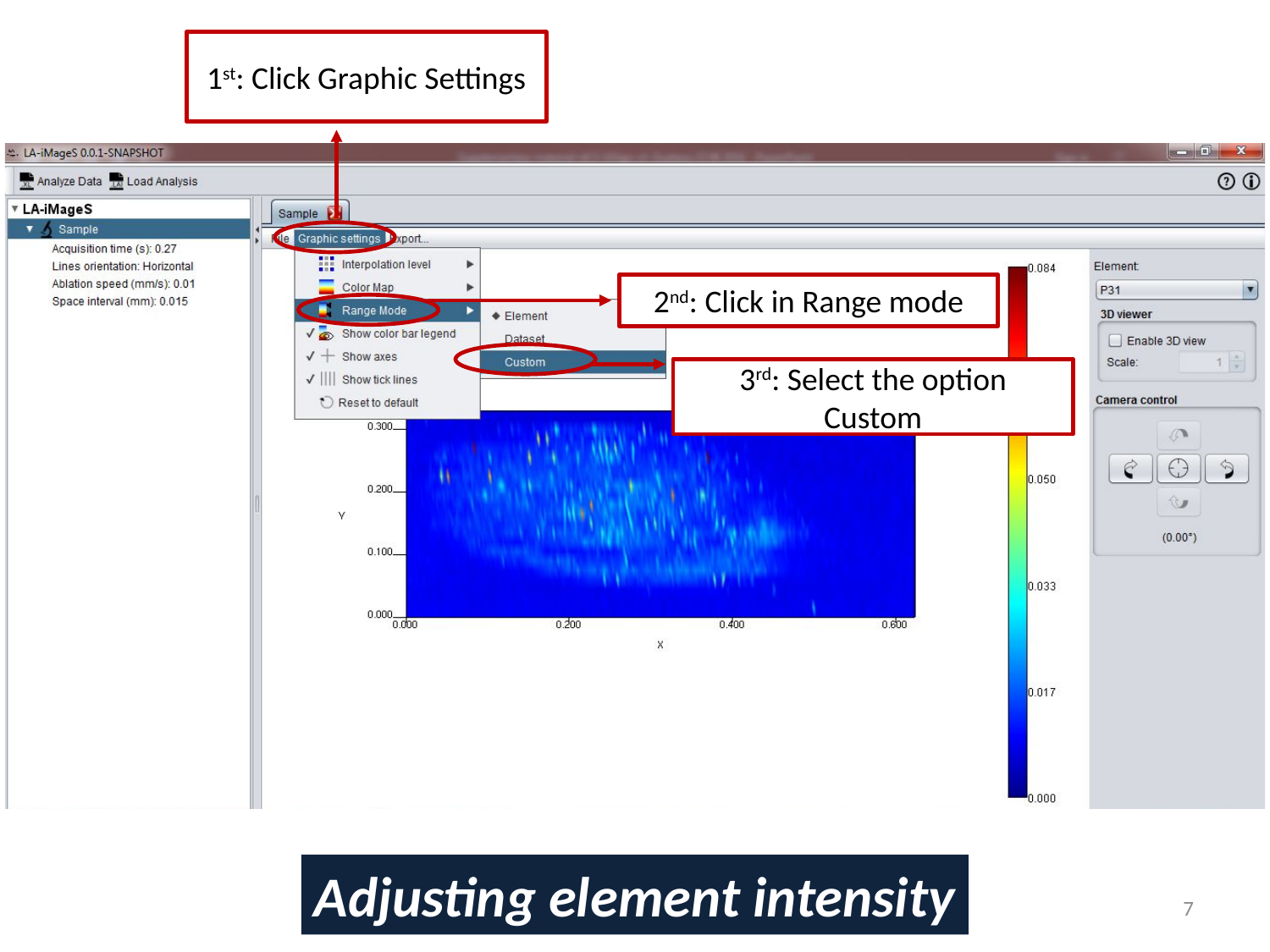

1st: Click Graphic Settings
2nd: Click in Range mode
3rd: Select the option
Custom
Adjusting element intensity
7

## Slide 8
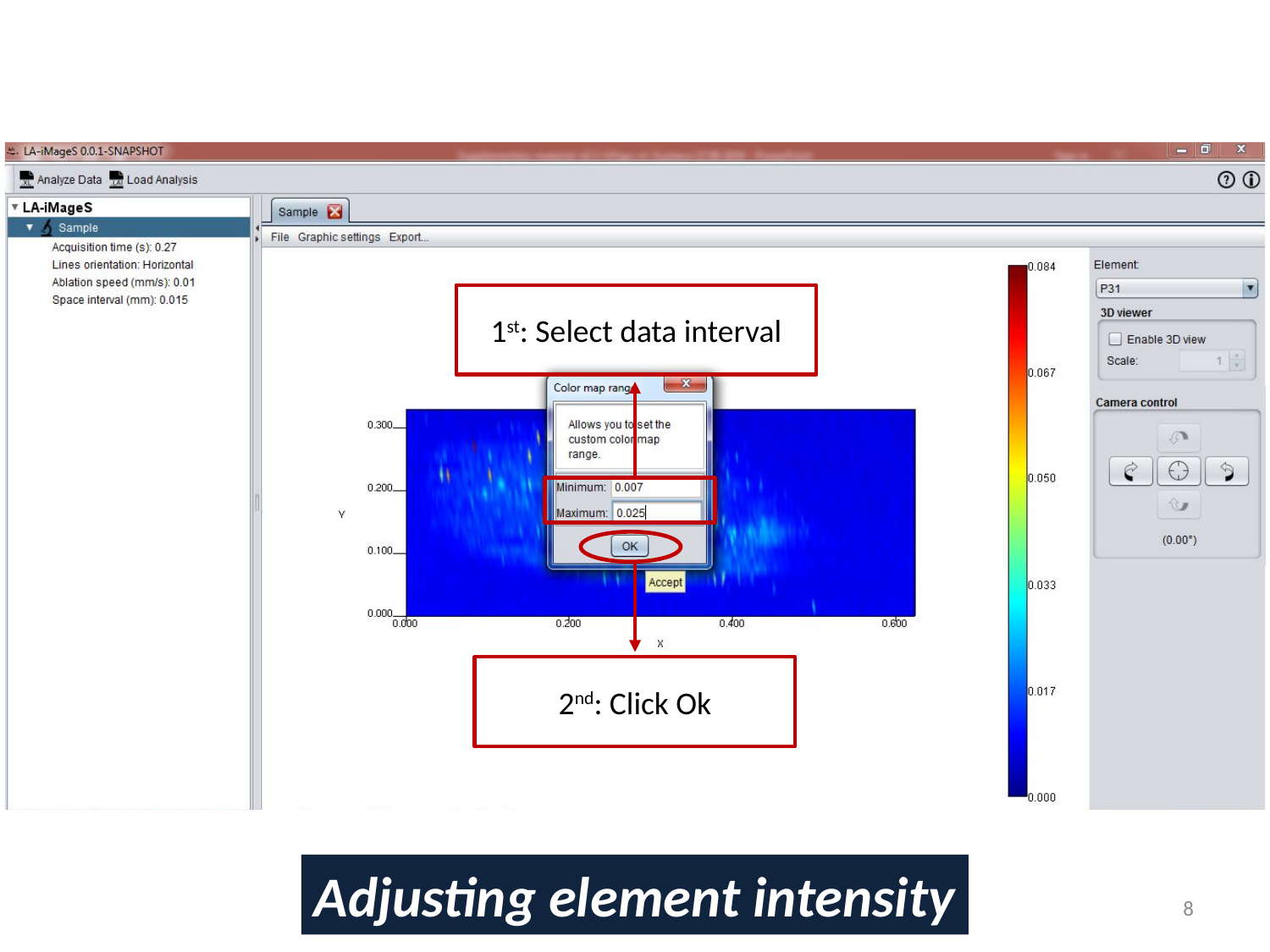

1st: Select data interval
2nd: Click Ok
Adjusting element intensity
8

## Slide 9
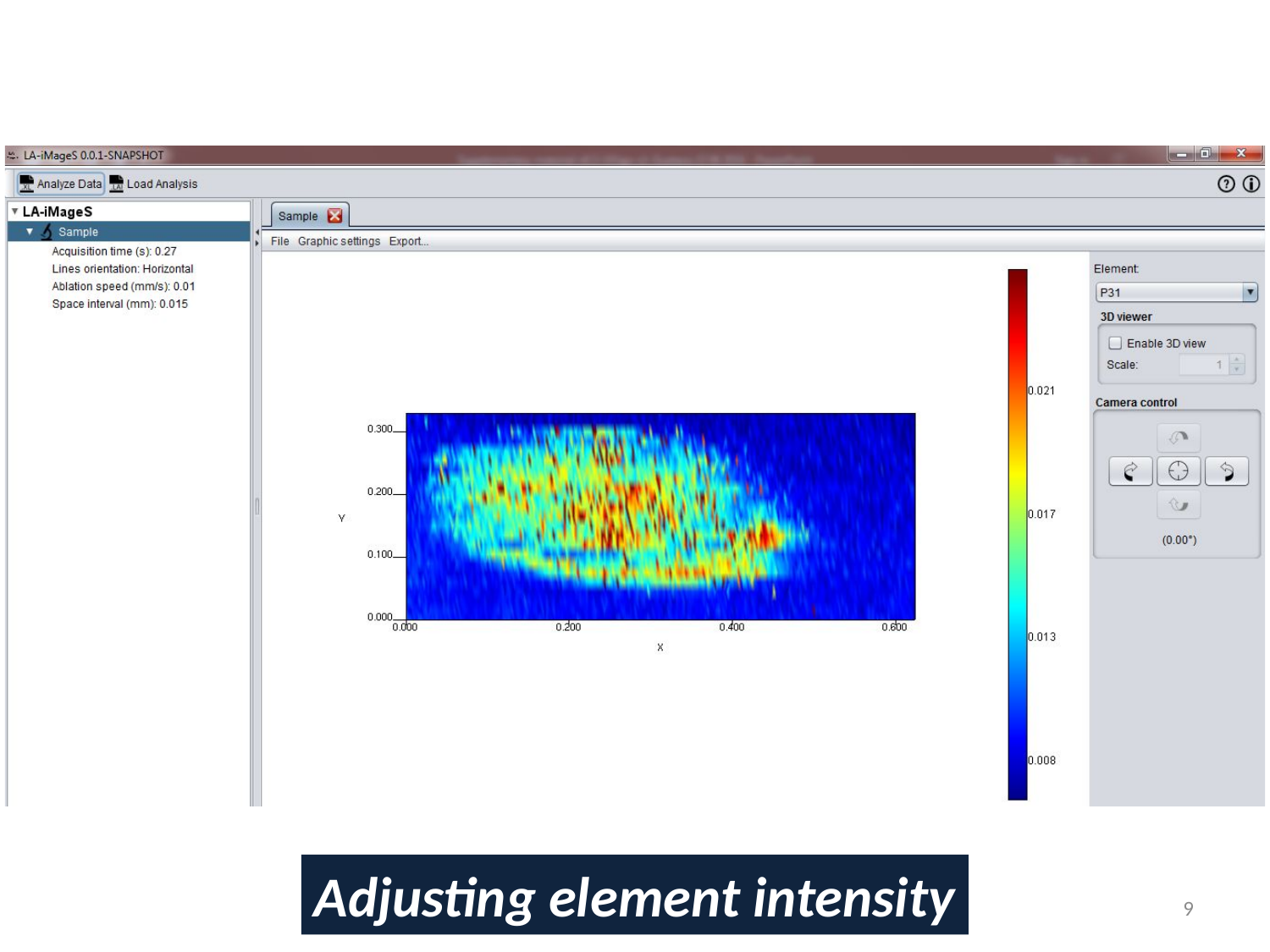

Adjusting element intensity
9

## Slide 10
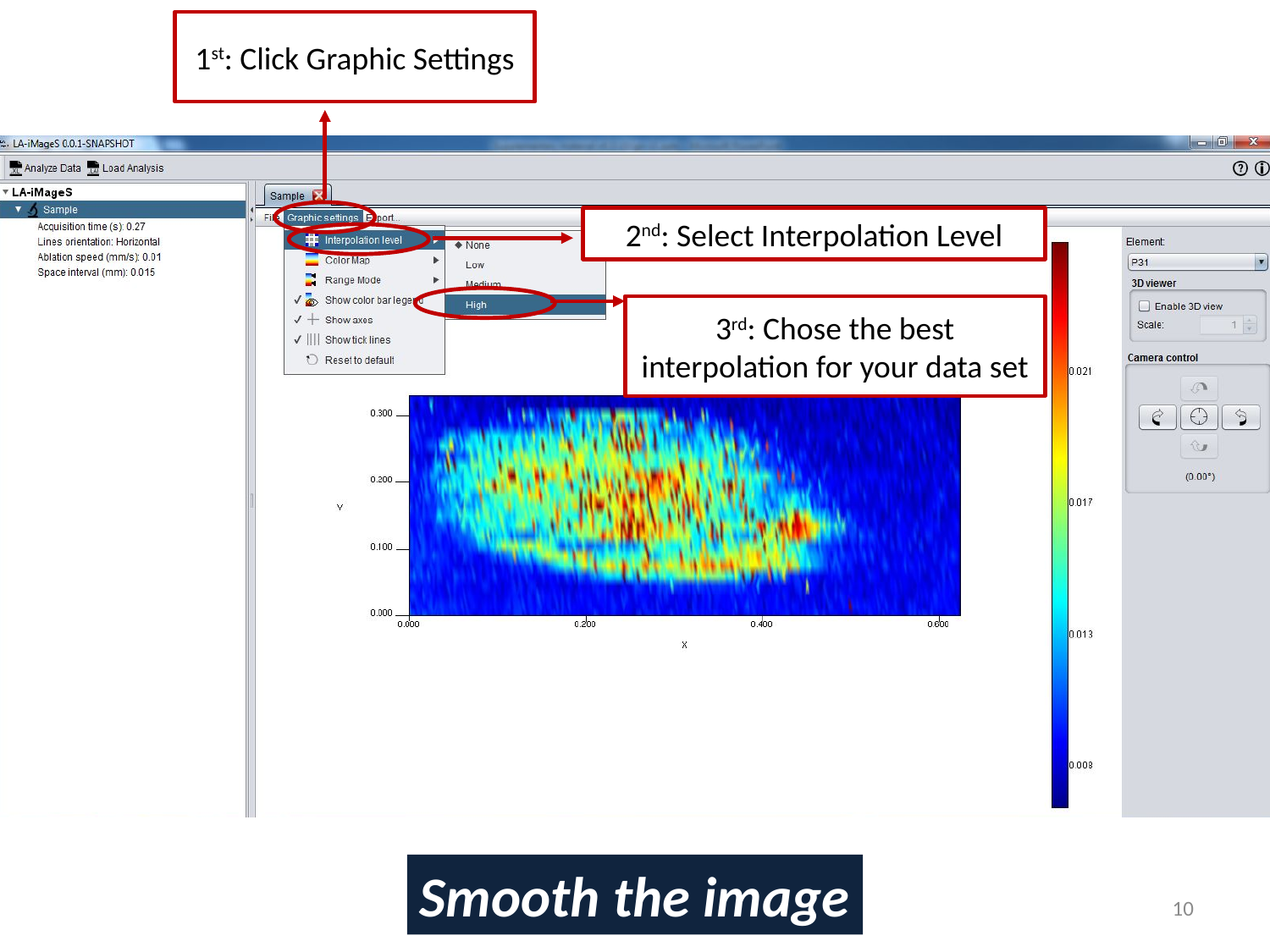

1st: Click Graphic Settings
2nd: Select Interpolation Level
3rd: Chose the best interpolation for your data set
Smooth the image
10

## Slide 11
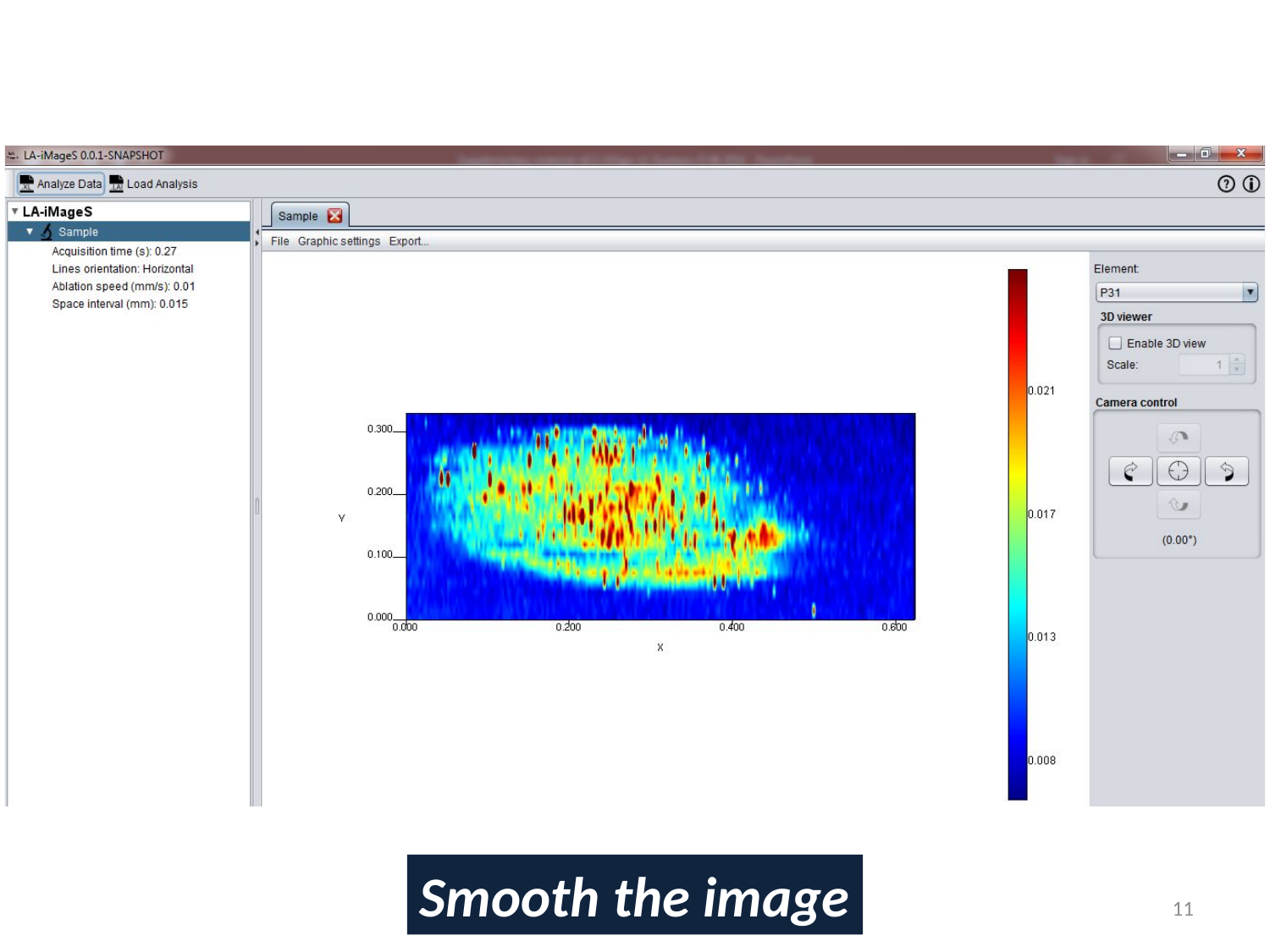

Smooth the image
11

## Slide 12
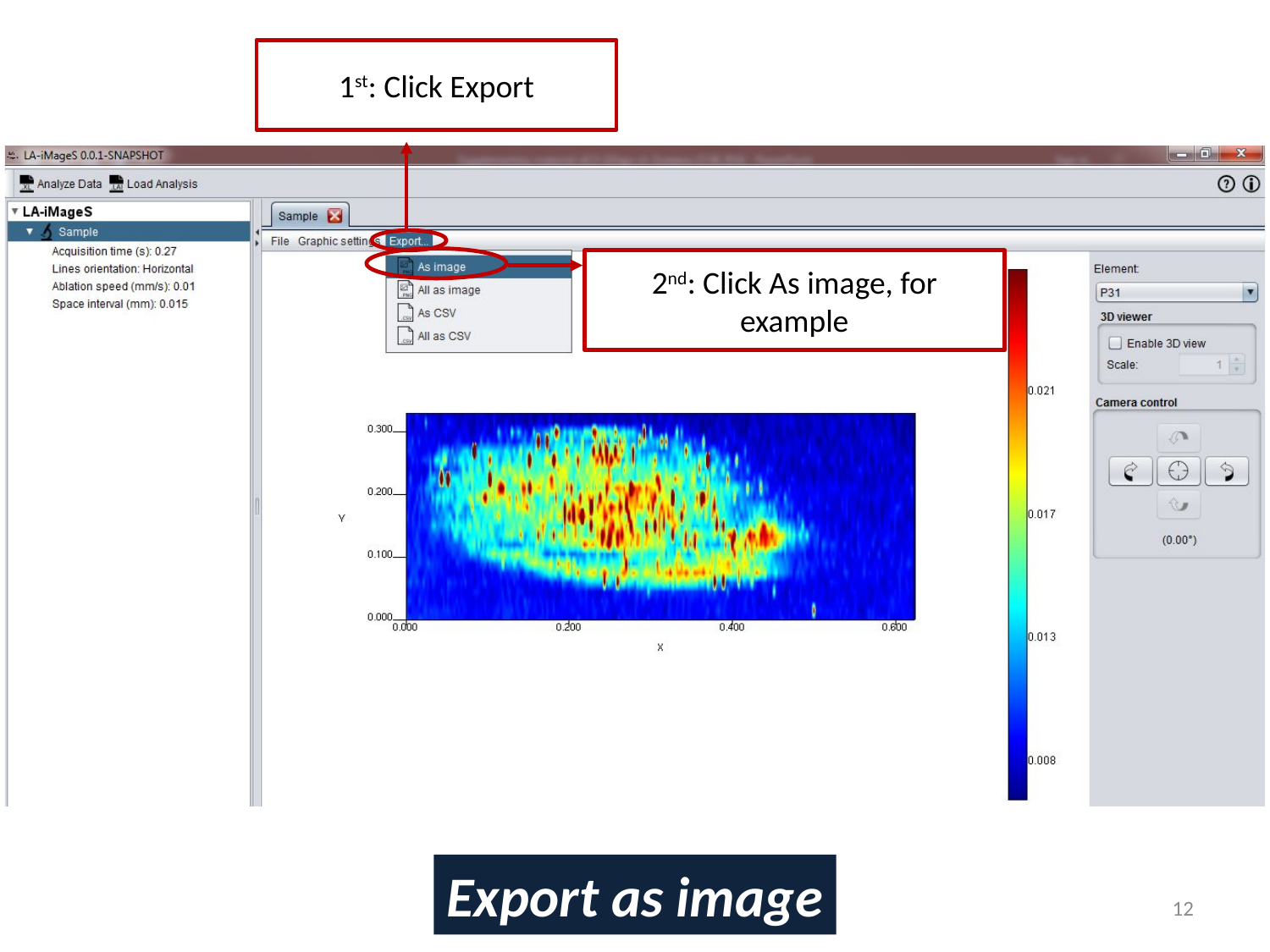

1st: Click Export
2nd: Click As image, for example
Export as image
12

## Slide 13
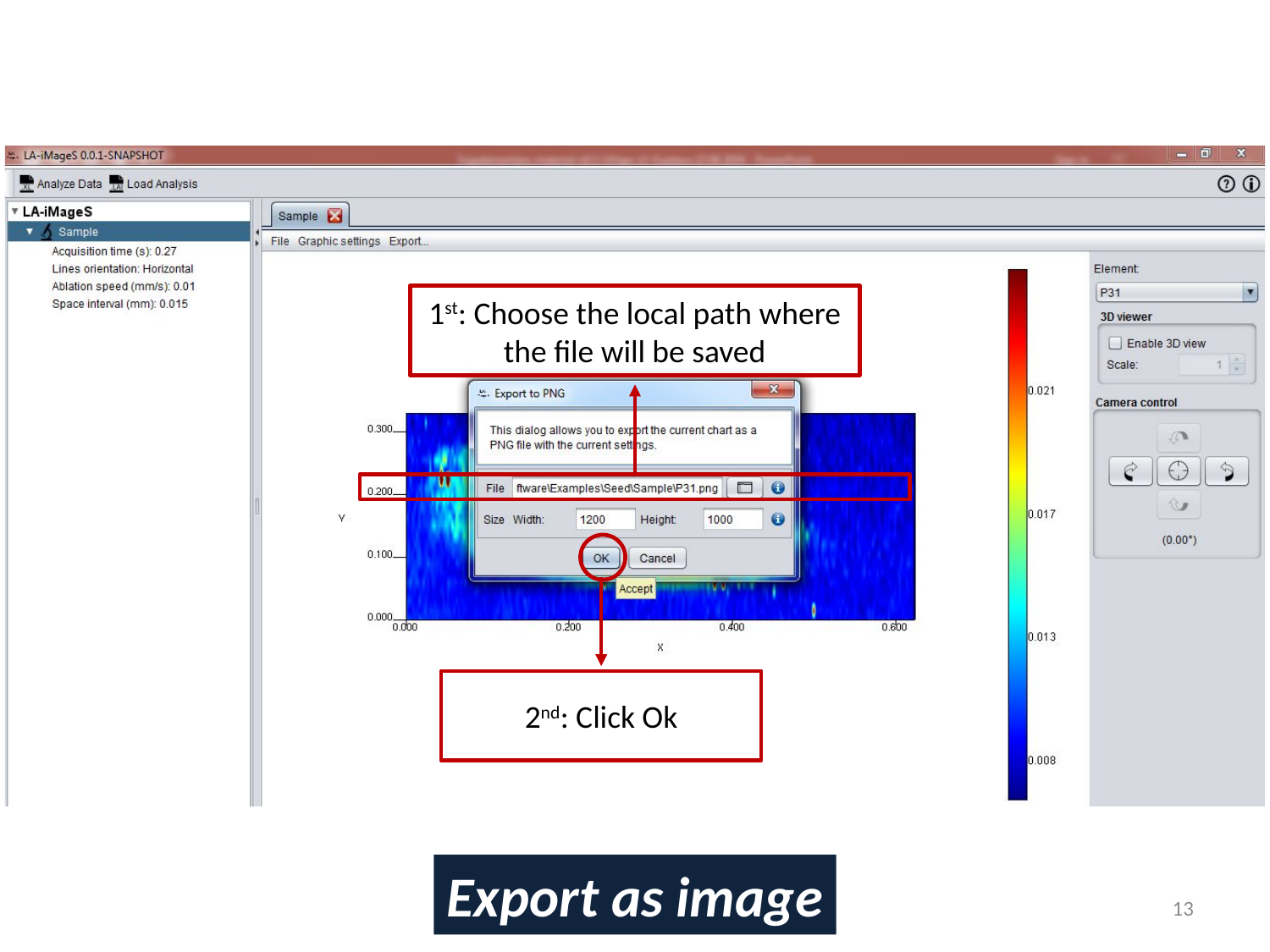

1st: Choose the local path where the file will be saved
2nd: Click Ok
Export as image
13

## Slide 14
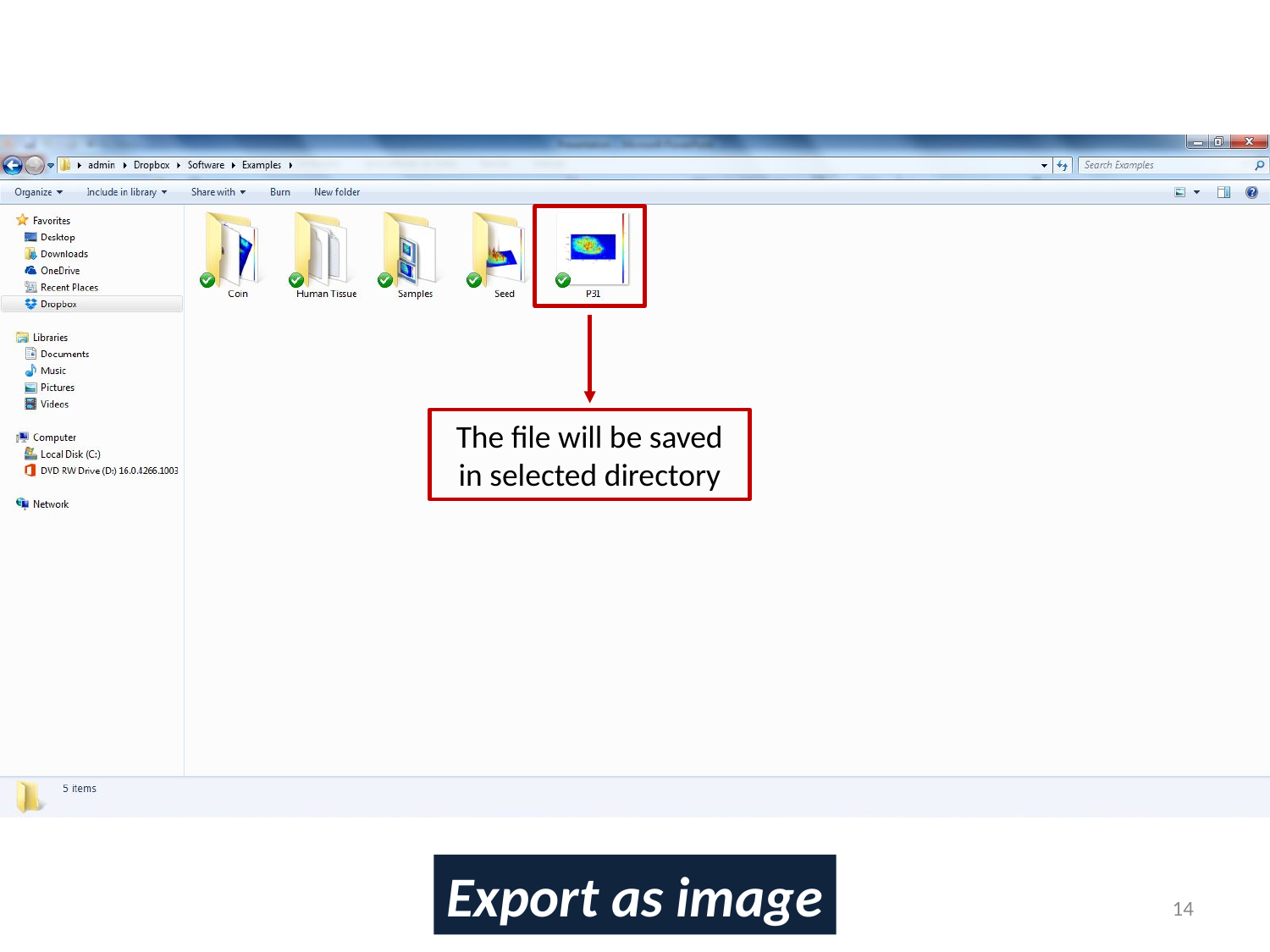

The file will be saved in selected directory
Export as image
14

## Slide 15
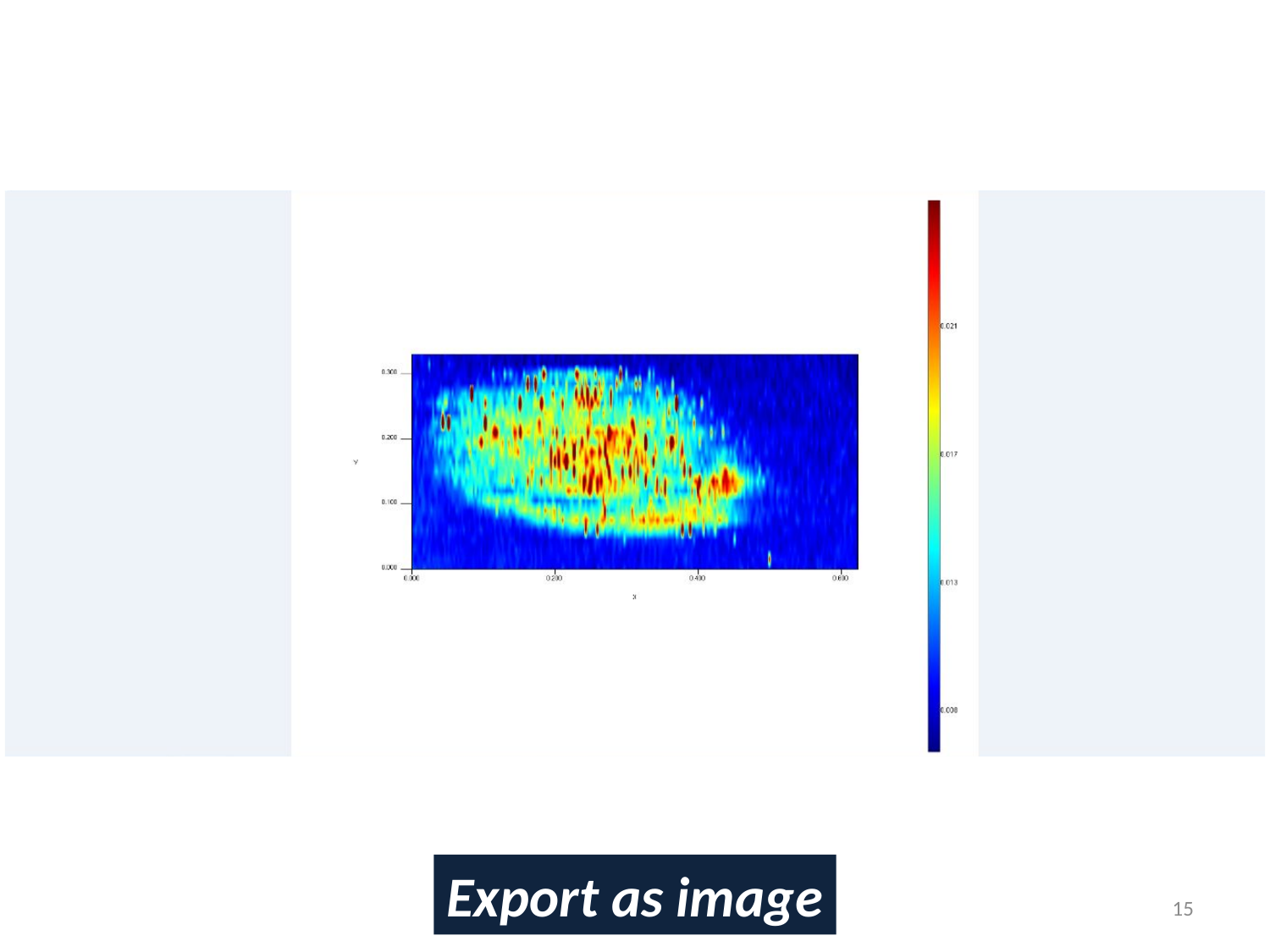

Export as image
15

## Slide 16
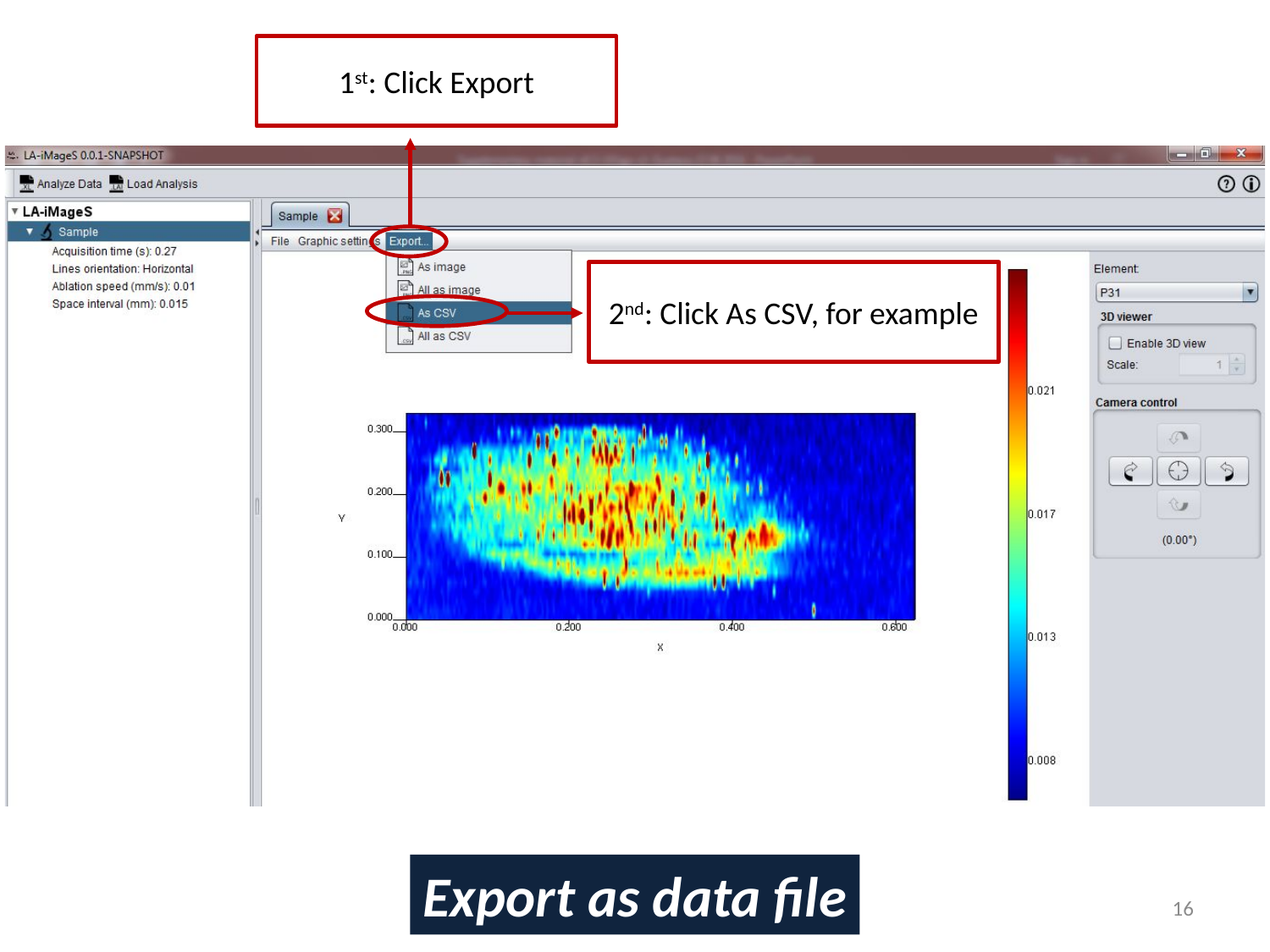

1st: Click Export
2nd: Click As CSV, for example
Export as data file
16

## Slide 17
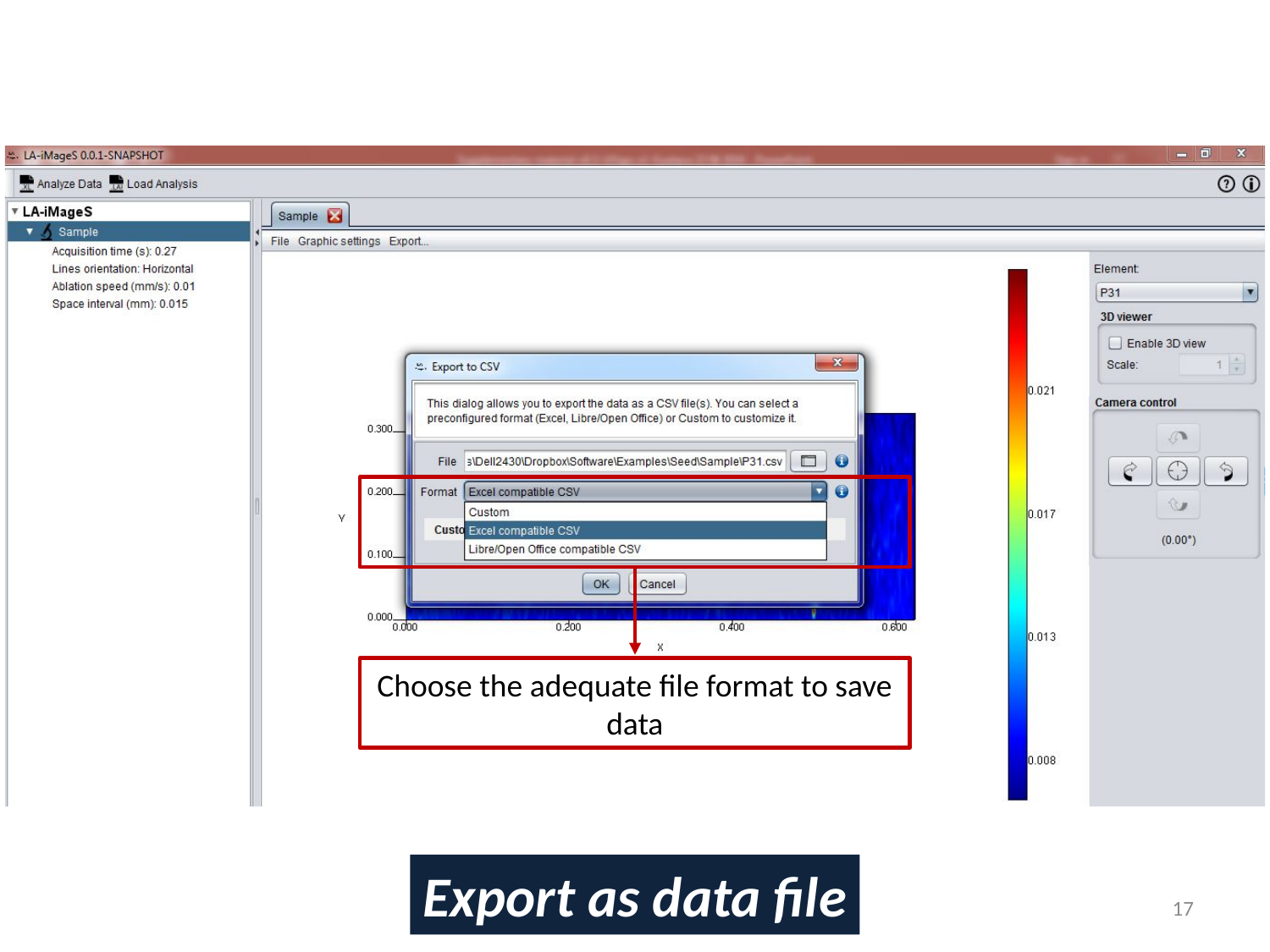

Choose the adequate file format to save data
Export as data file
17

## Slide 18
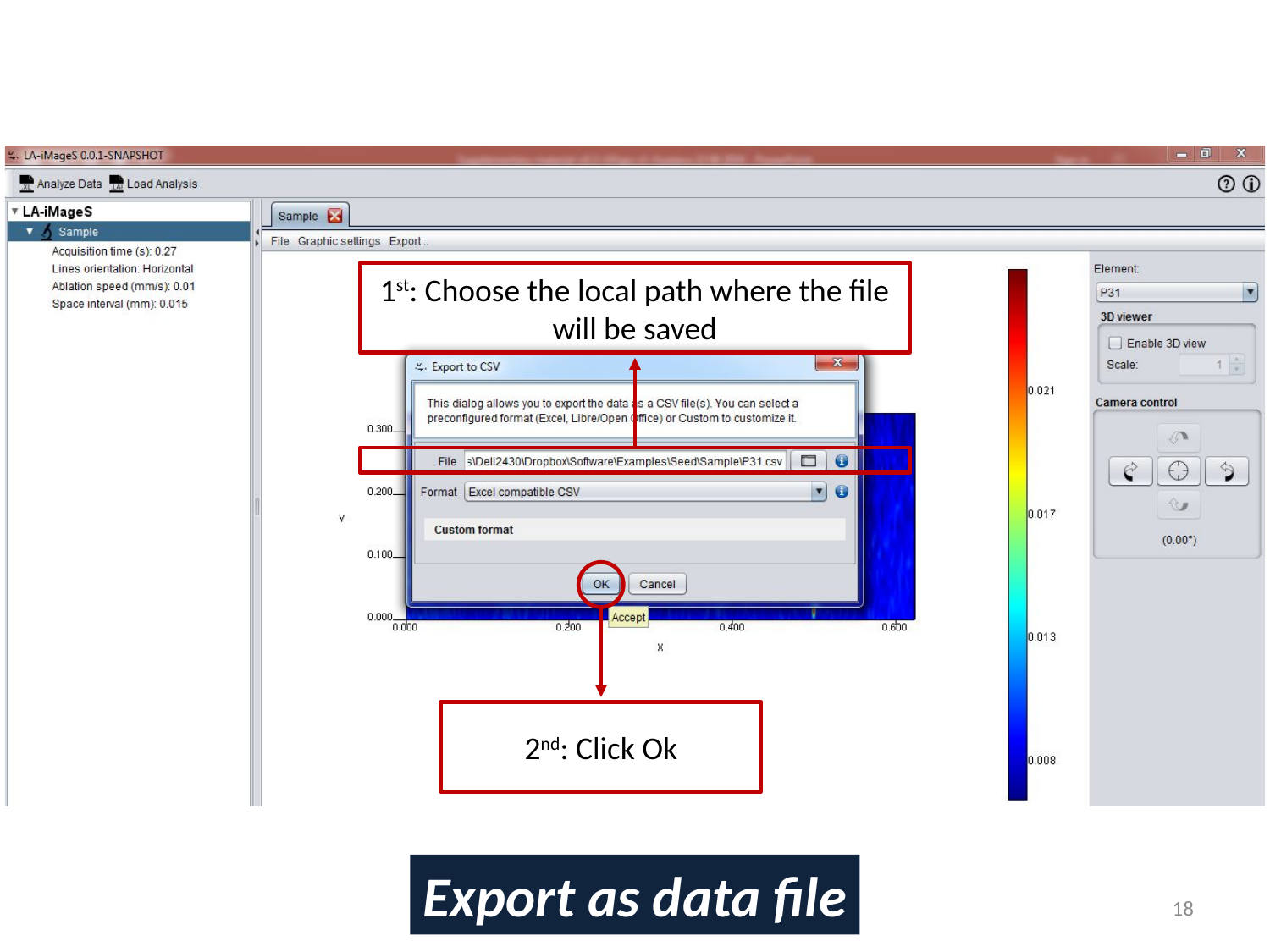

1st: Choose the local path where the file will be saved
2nd: Click Ok
Export as data file
18

## Slide 19
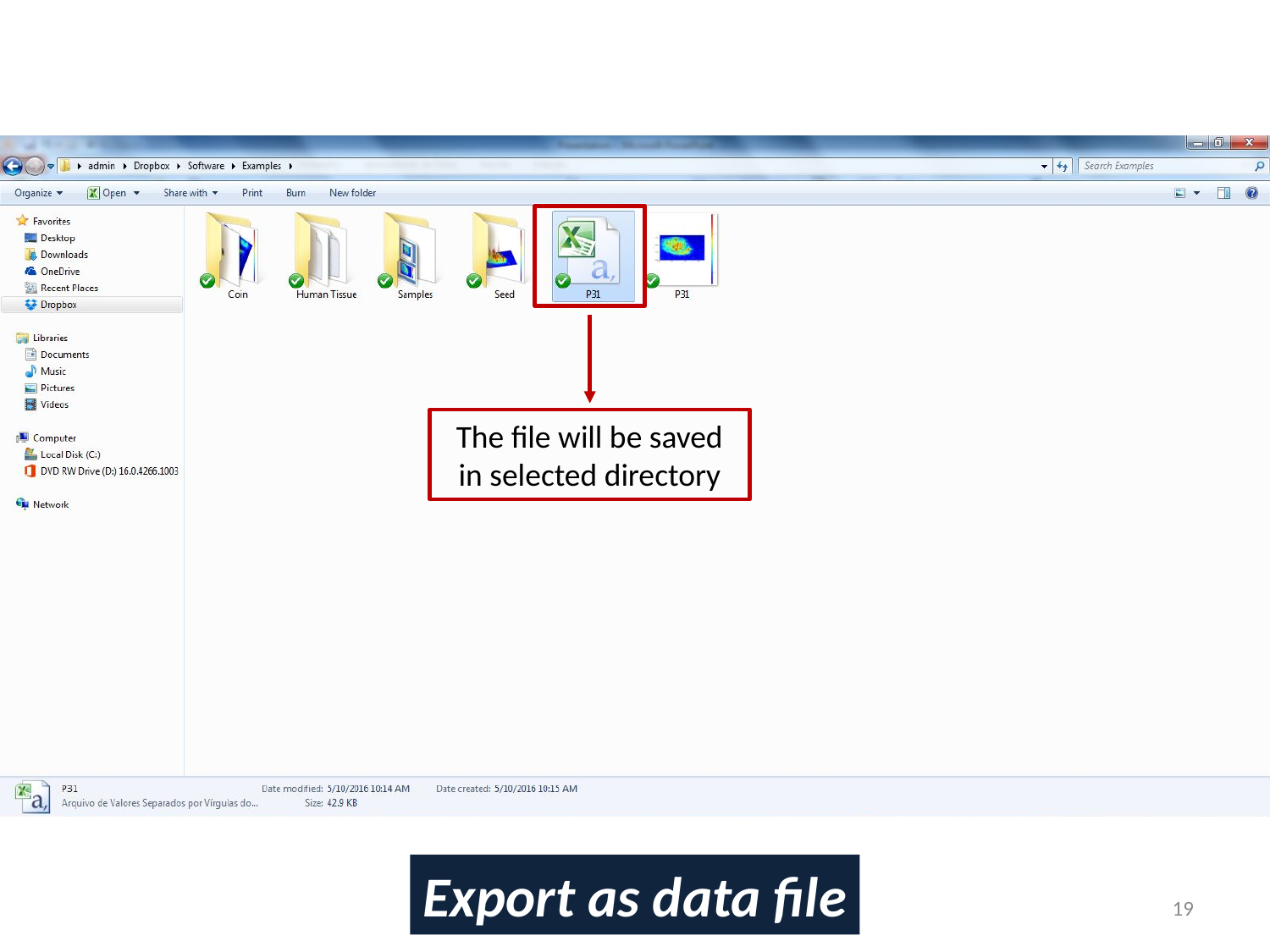

The file will be saved in selected directory
Export as data file
19

## Slide 20
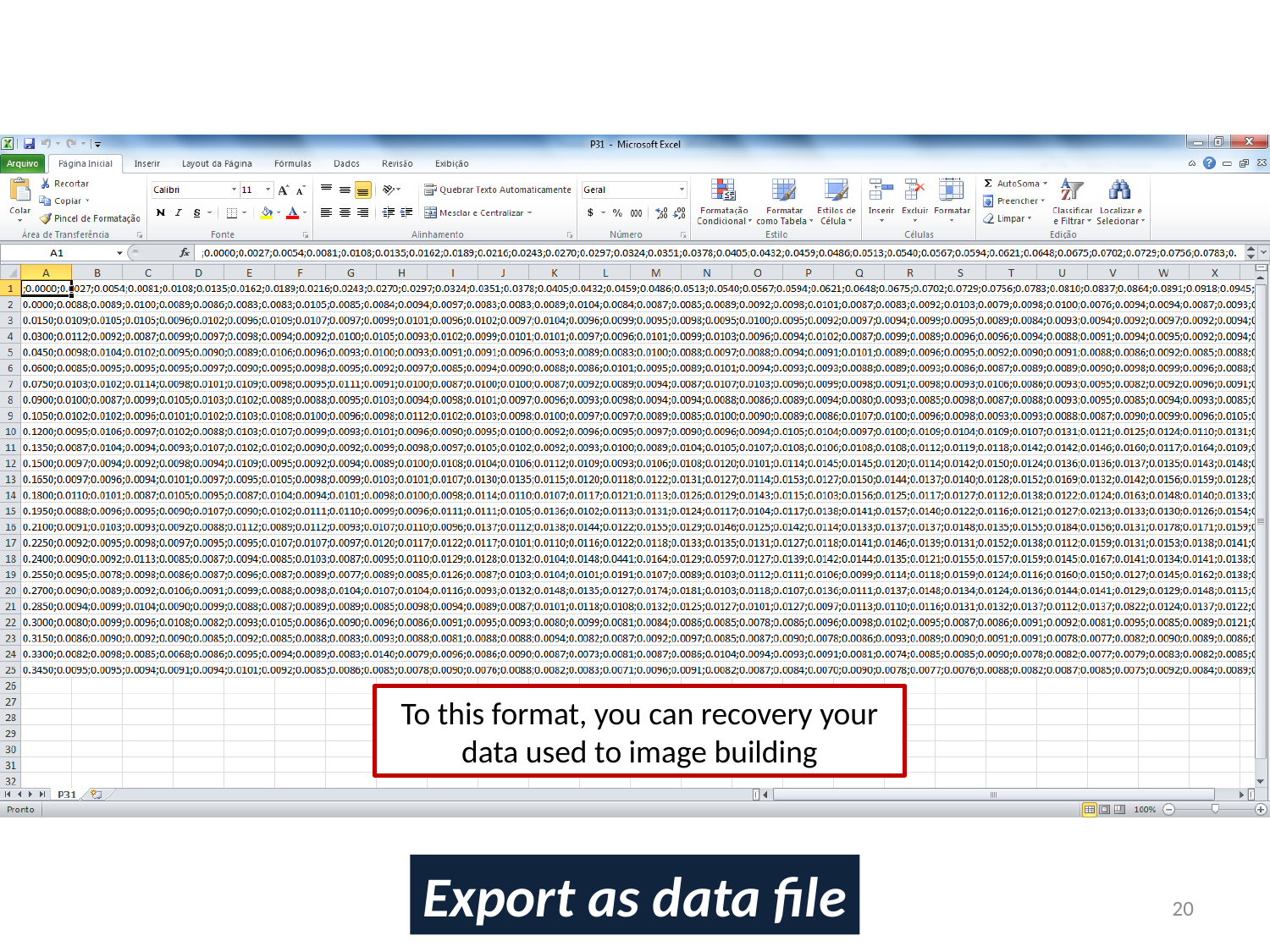

To this format, you can recovery your data used to image building
Export as data file
20

## Slide 21
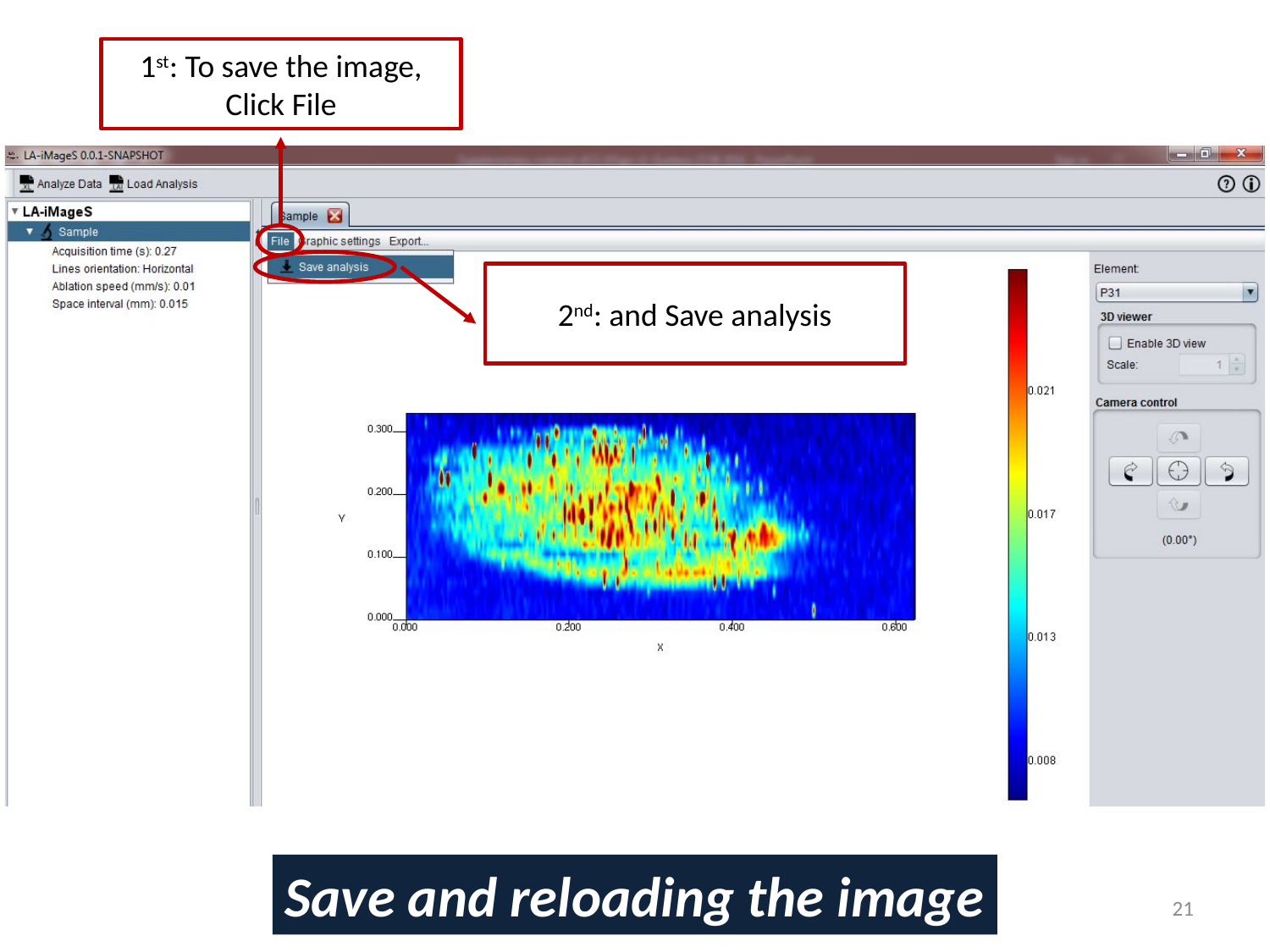

1st: To save the image, Click File
2nd: and Save analysis
Save and reloading the image
21

## Slide 22
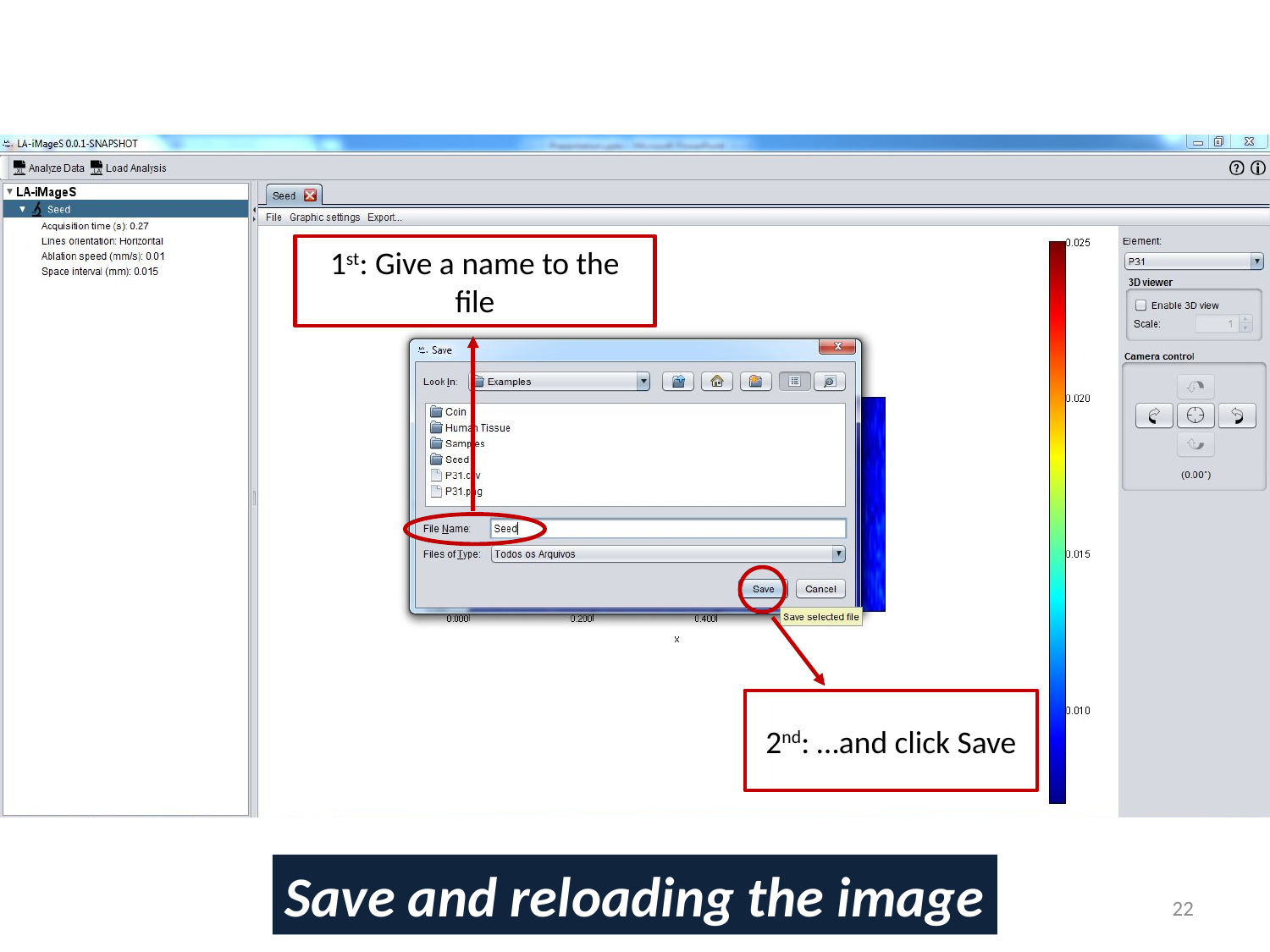

1st: Give a name to the file
2nd: …and click Save
Save and reloading the image
22

## Slide 23
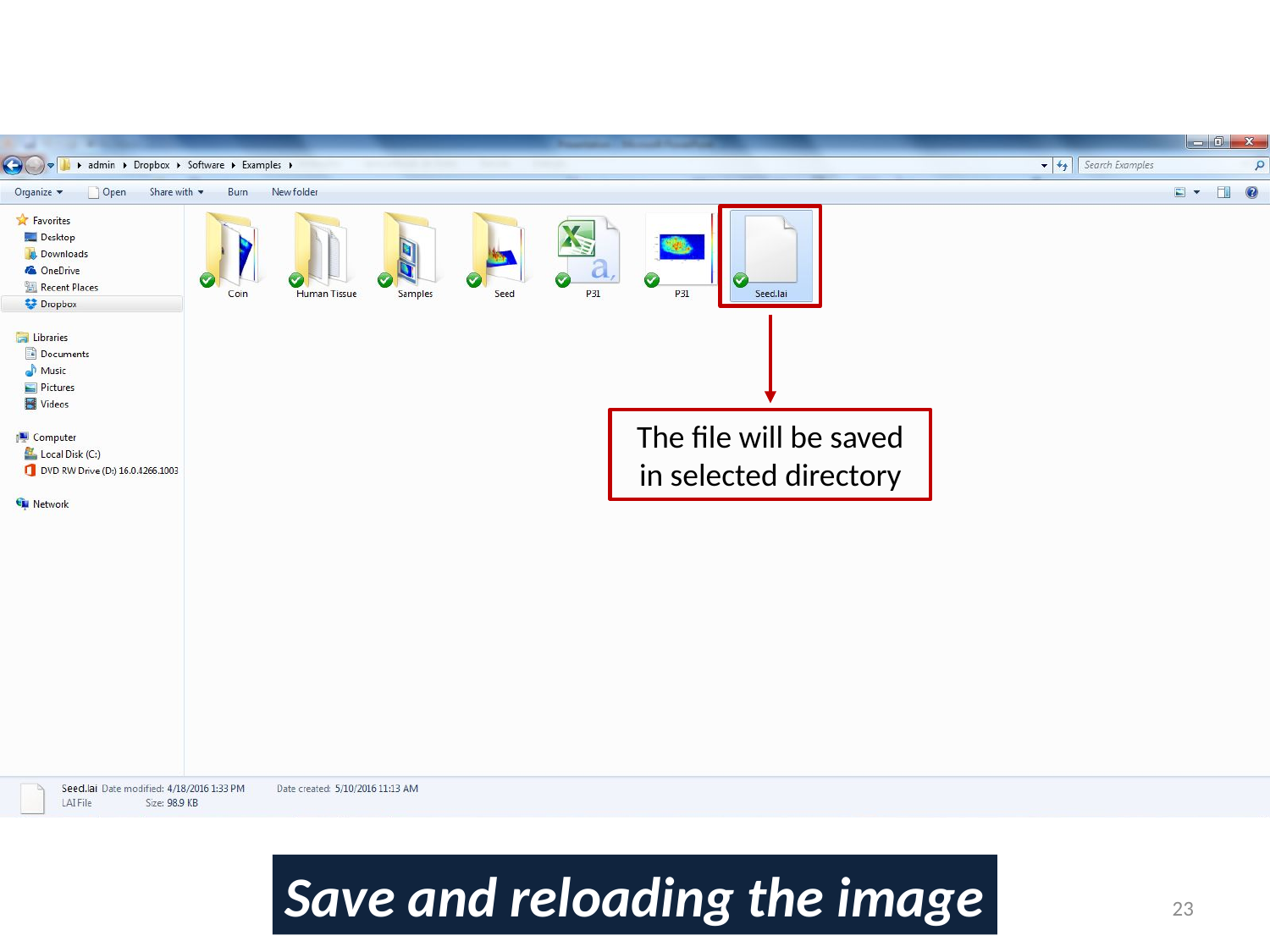

The file will be saved in selected directory
Save and reloading the image
23

## Slide 24
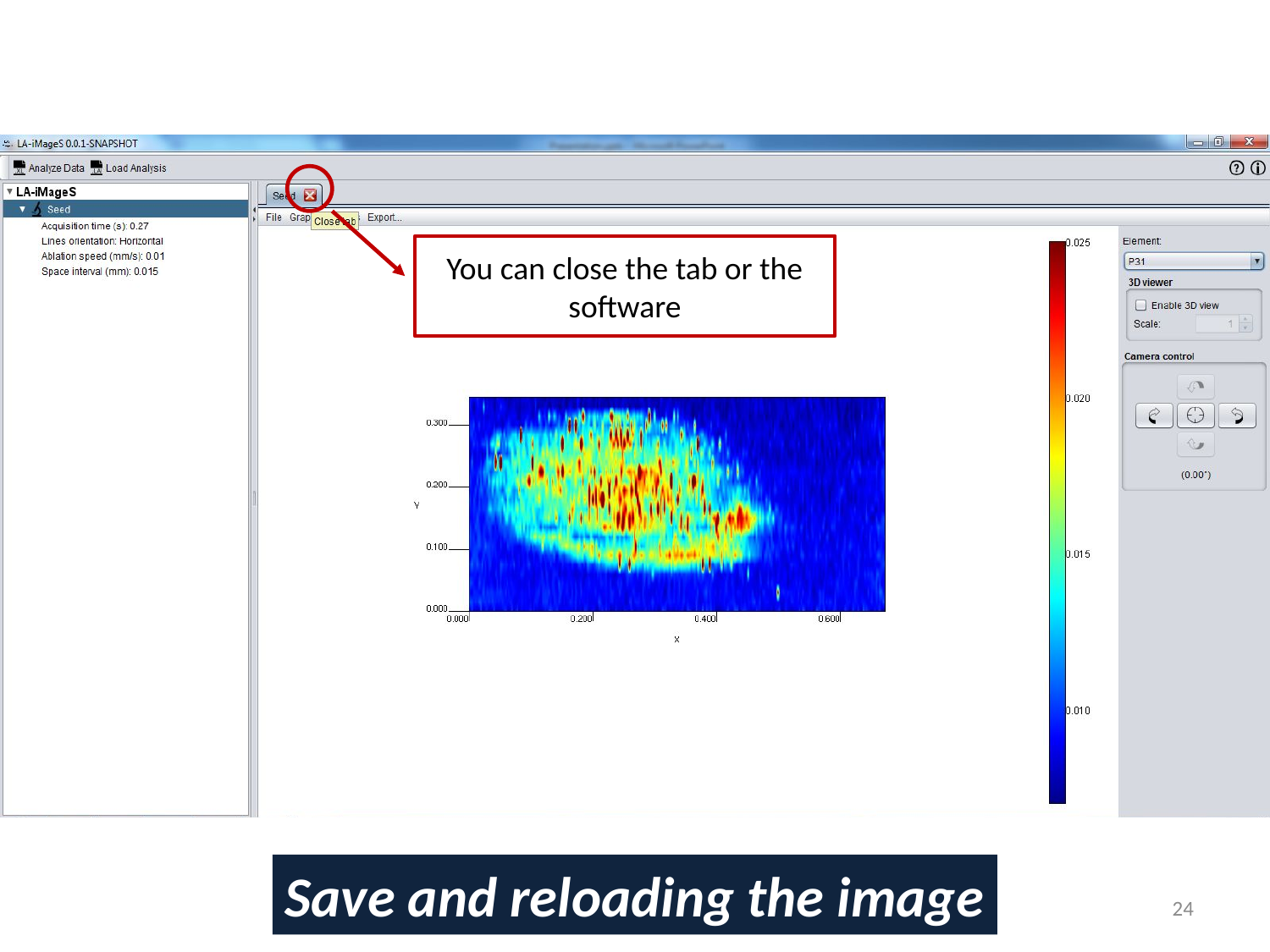

You can close the tab or the software
Save and reloading the image
24

## Slide 25
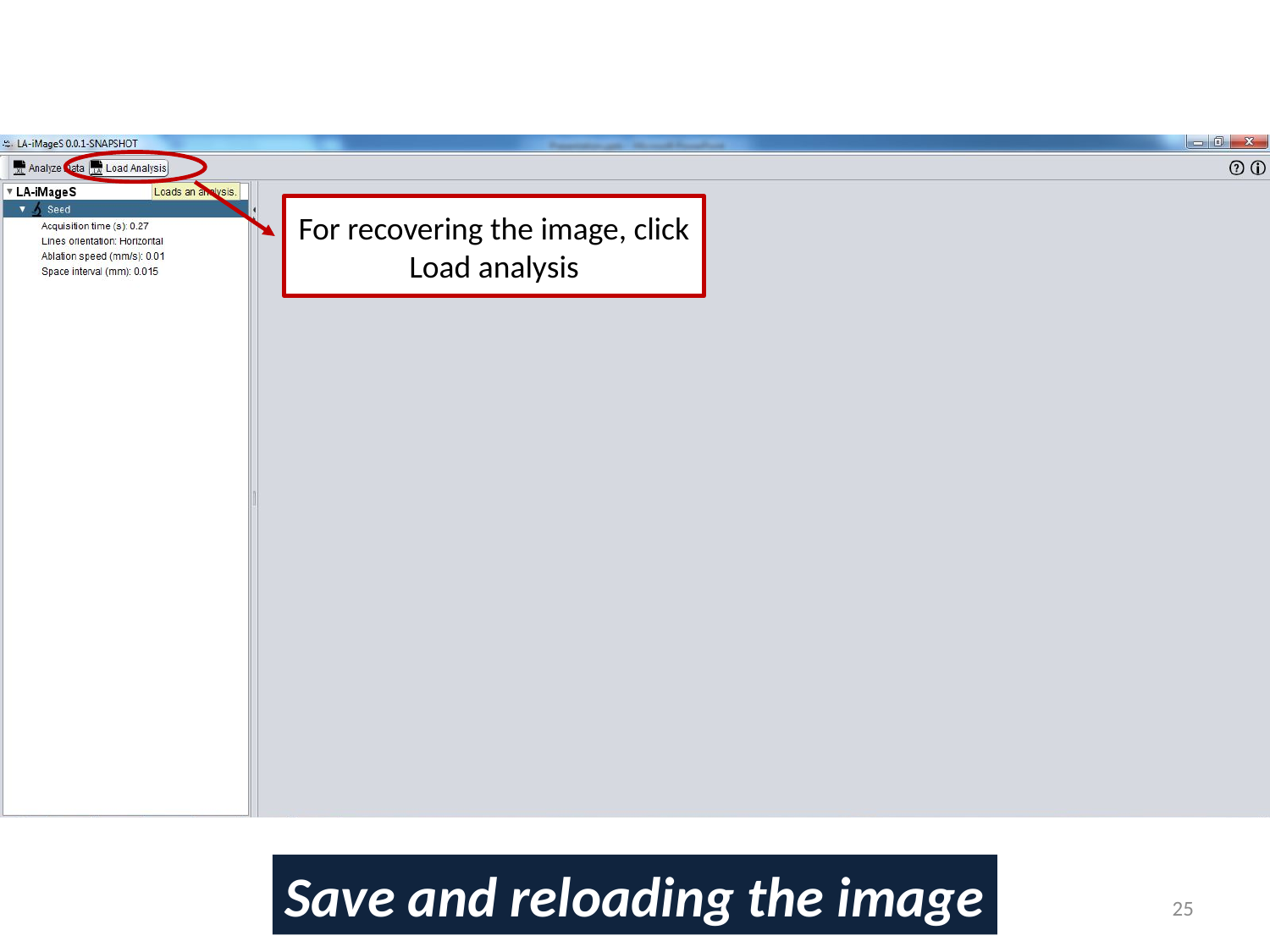

For recovering the image, click Load analysis
Save and reloading the image
25

## Slide 26
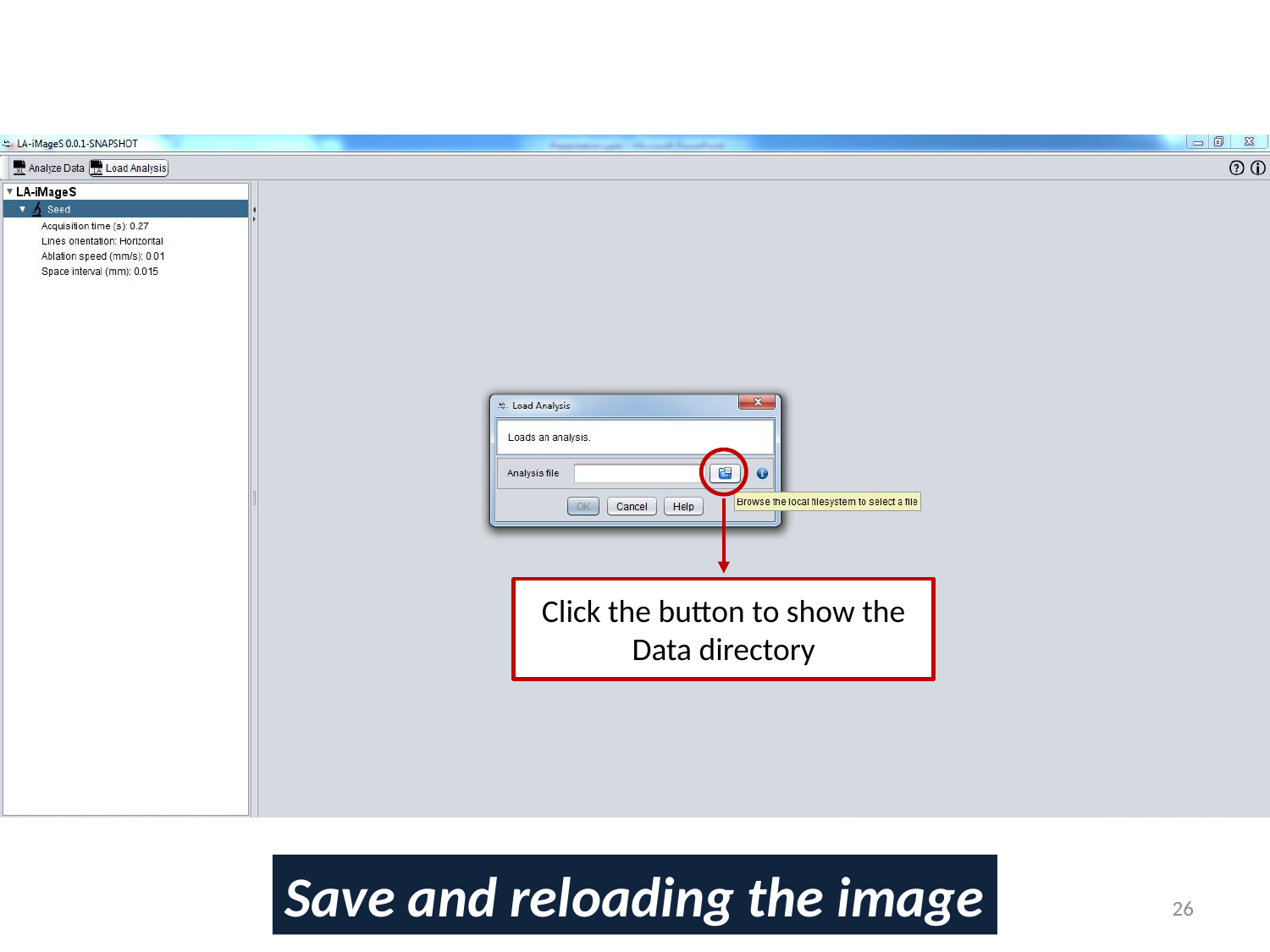

Click the button to show the Data directory
Save and reloading the image
26

## Slide 27
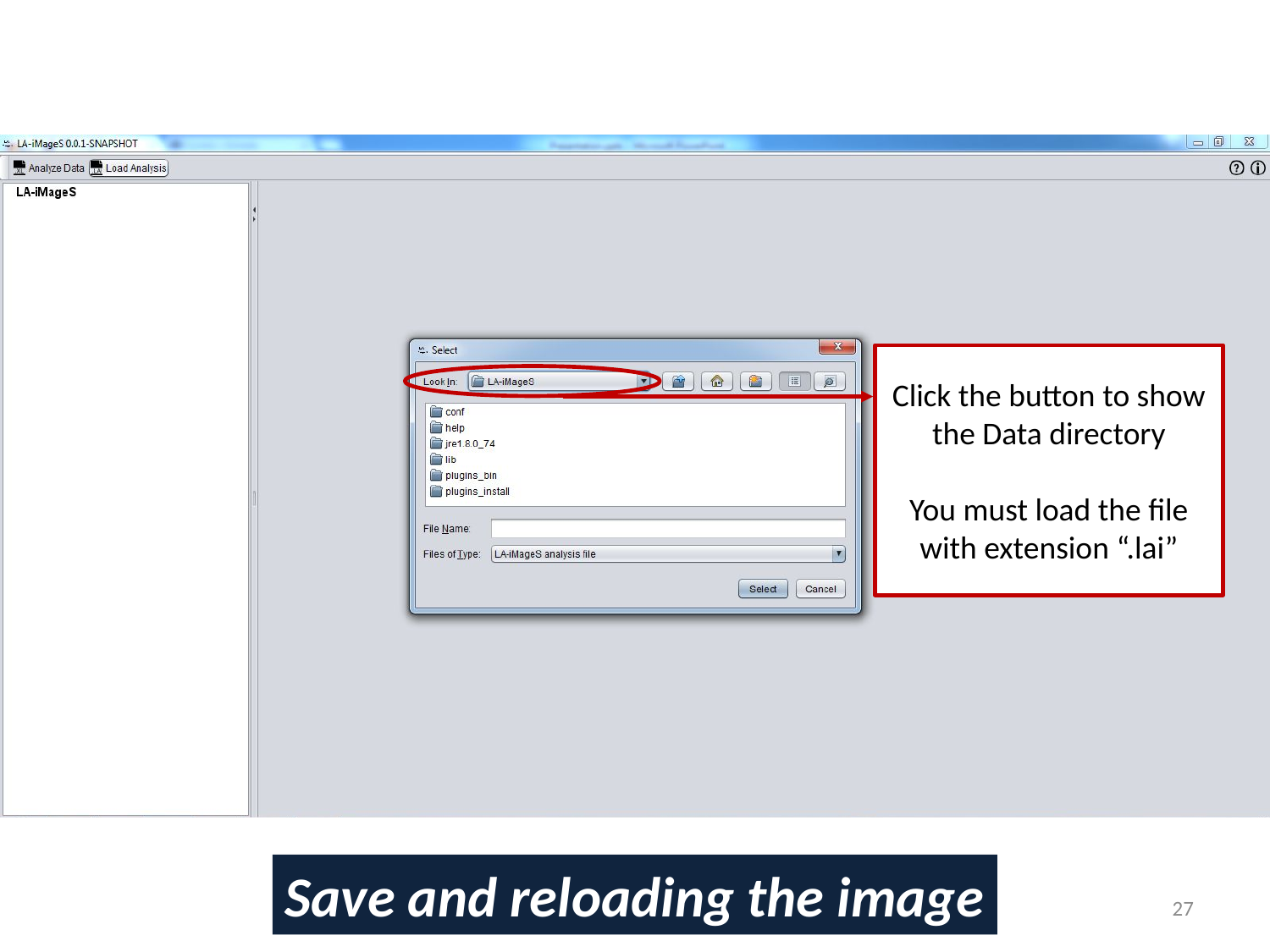

Click the button to show the Data directory
You must load the file with extension “.lai”
Save and reloading the image
27

## Slide 28
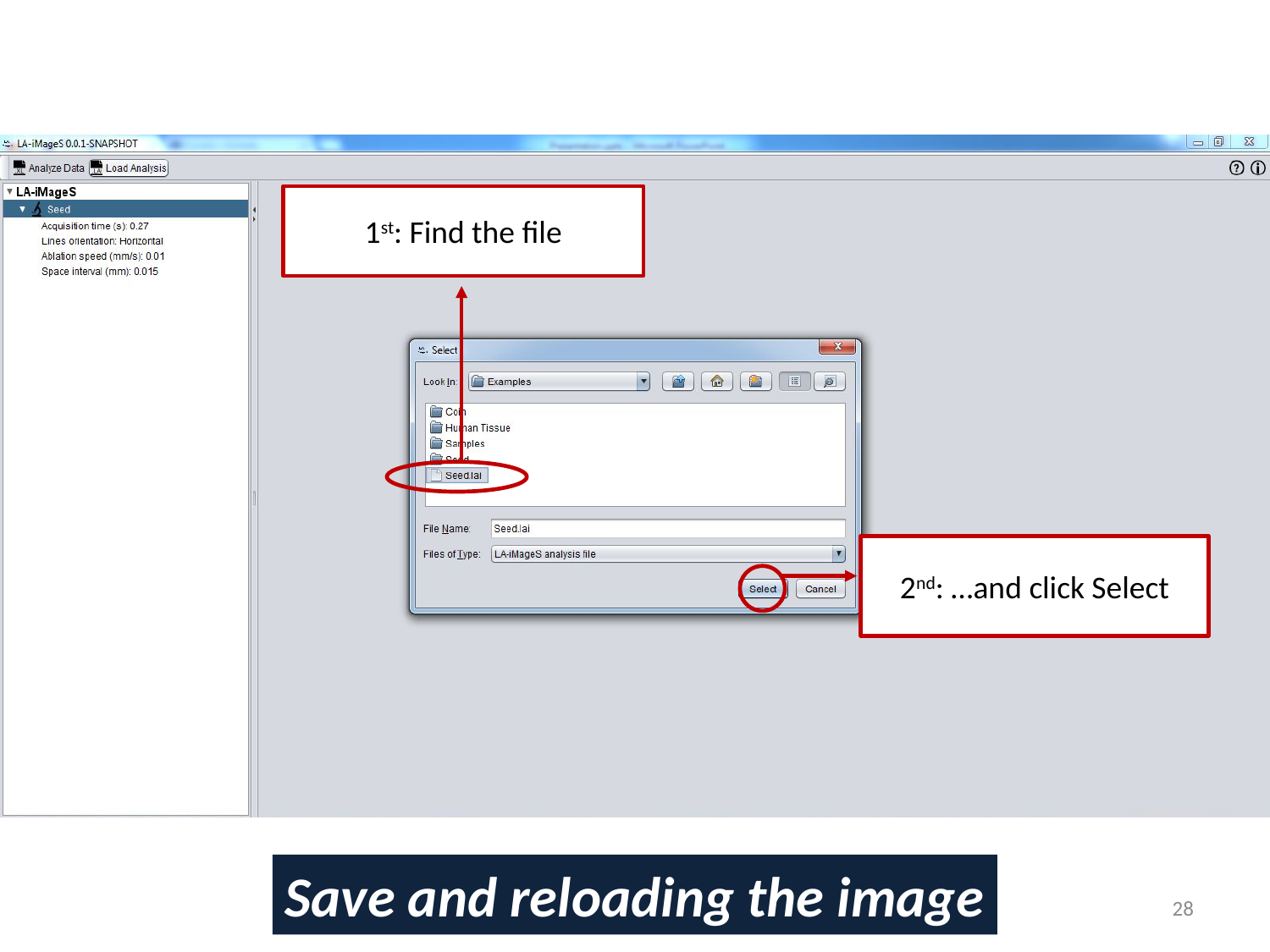

1st: Find the file
2nd: …and click Select
Save and reloading the image
28

## Slide 29
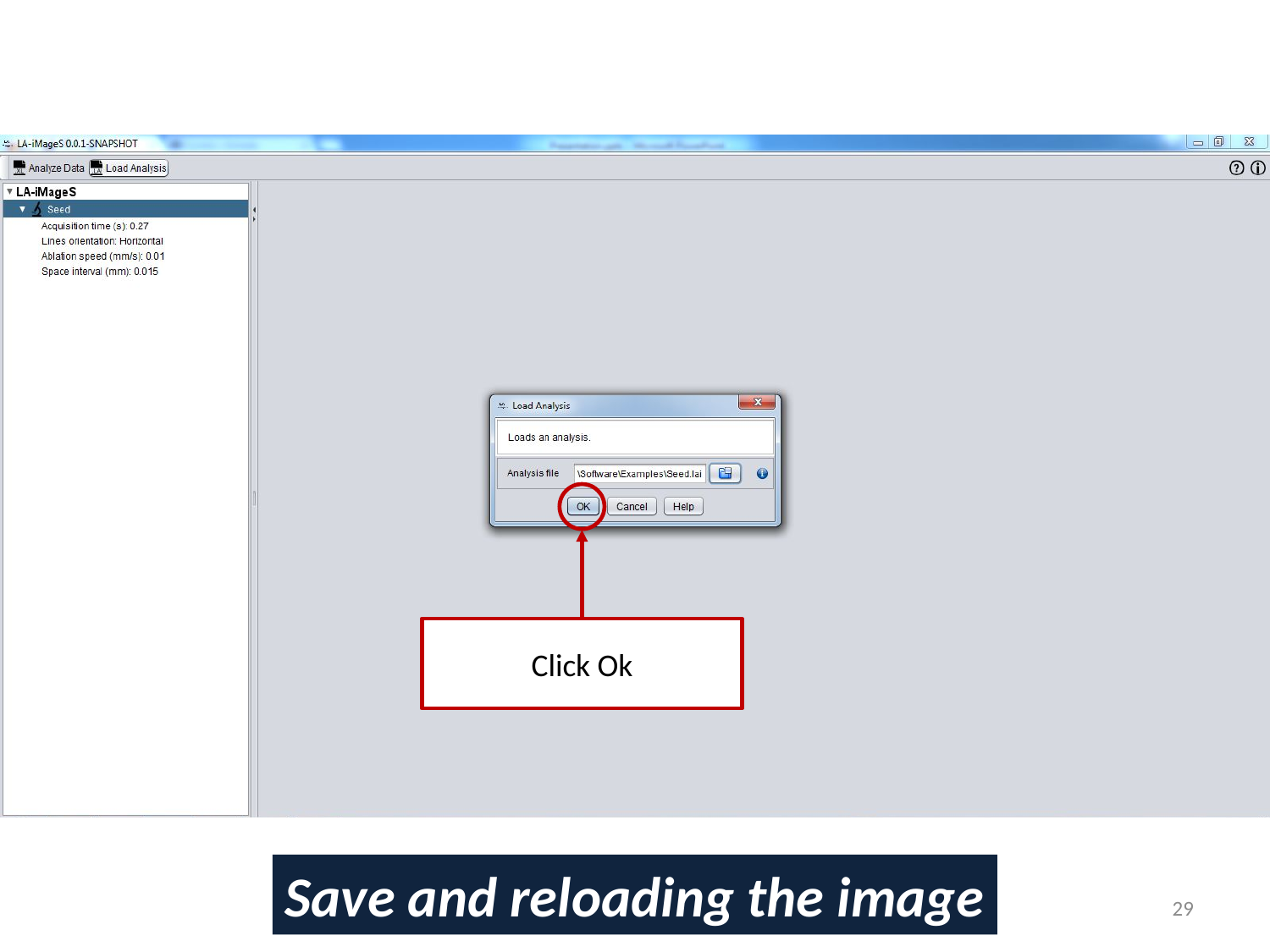

Click Ok
Save and reloading the image
29

## Slide 30
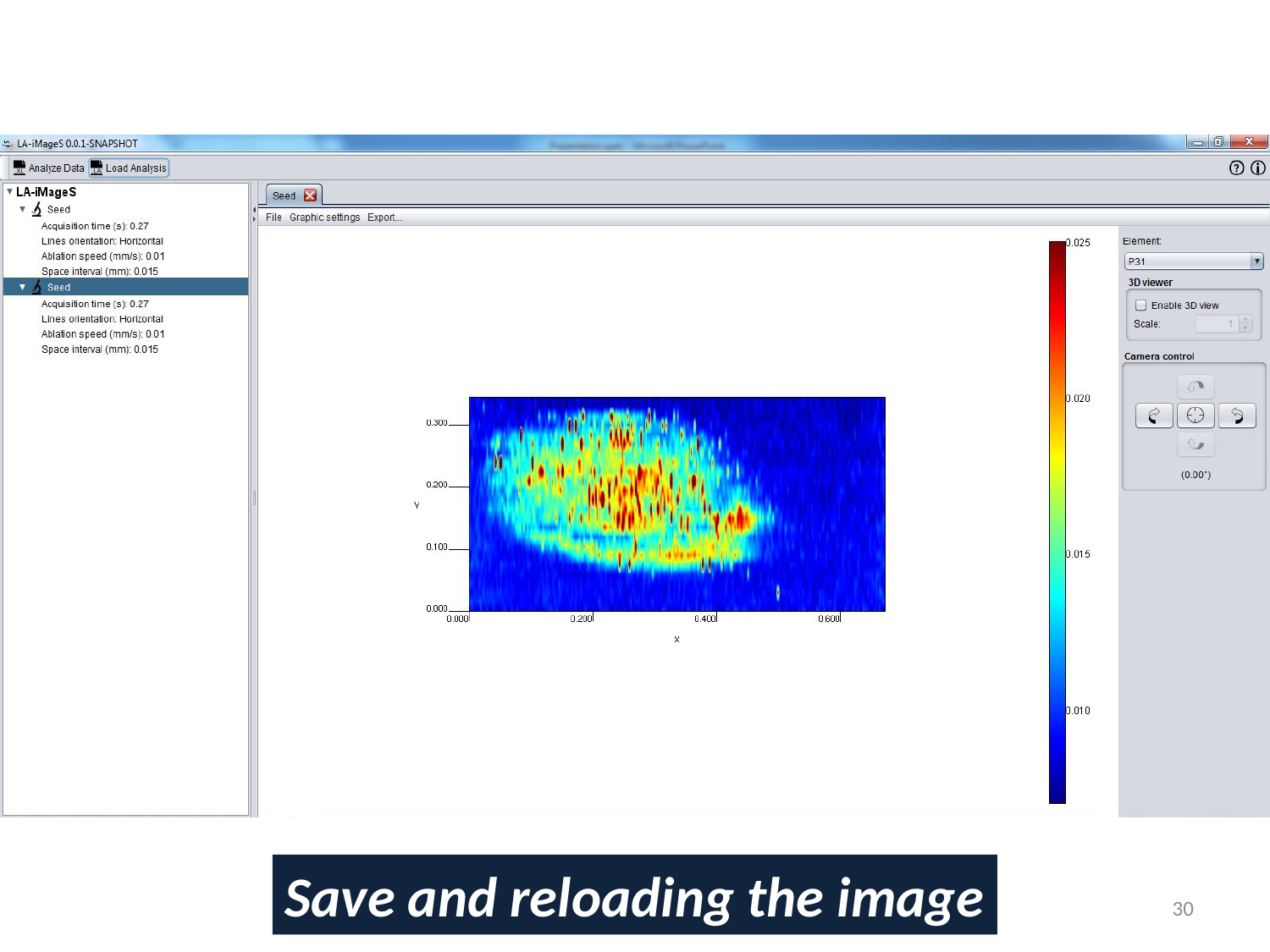

Save and reloading the image
30

## Slide 31
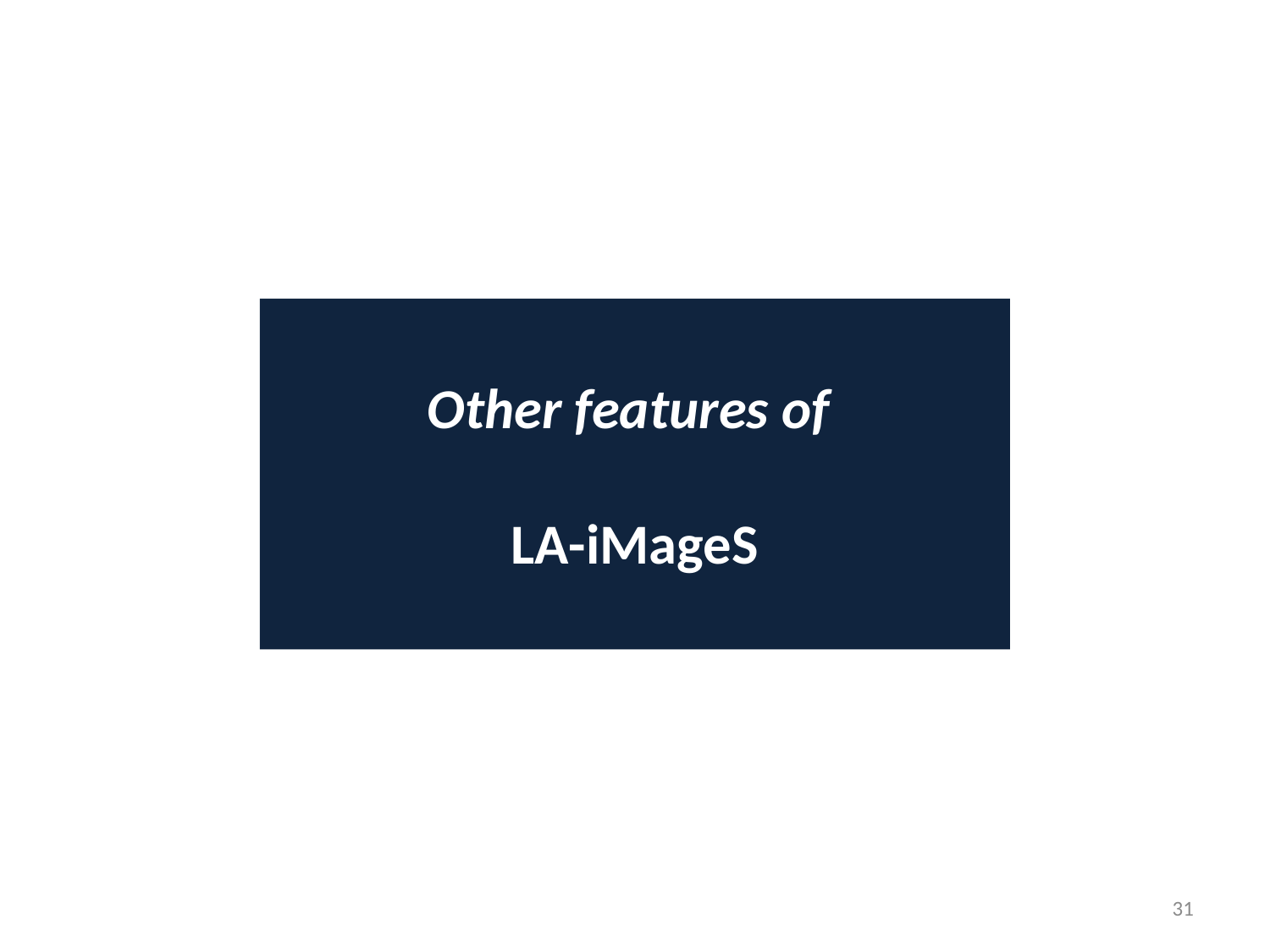

Other features of
LA-iMageS
31

## Slide 32
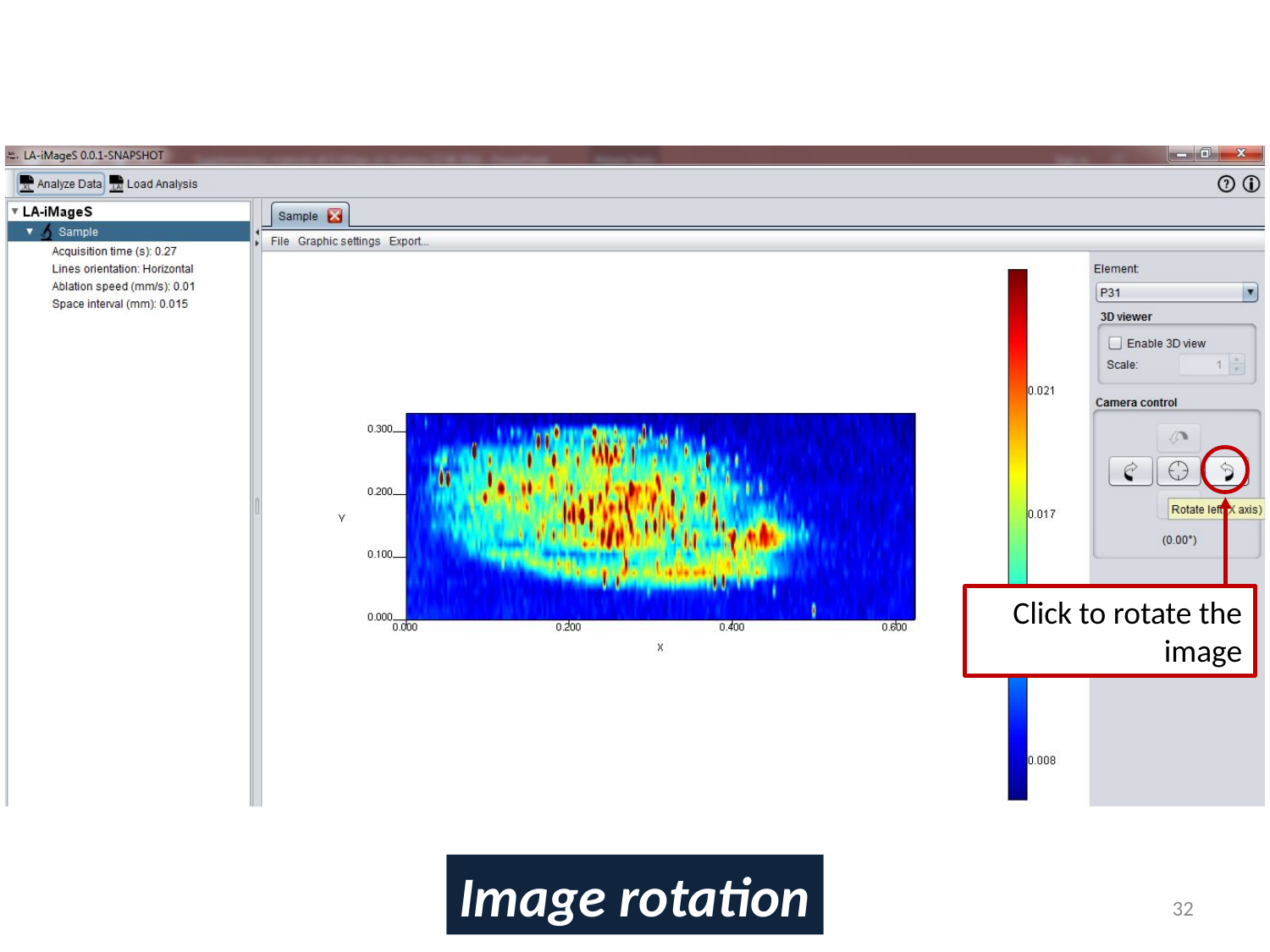

Click to rotate the image
Image rotation
32

## Slide 33
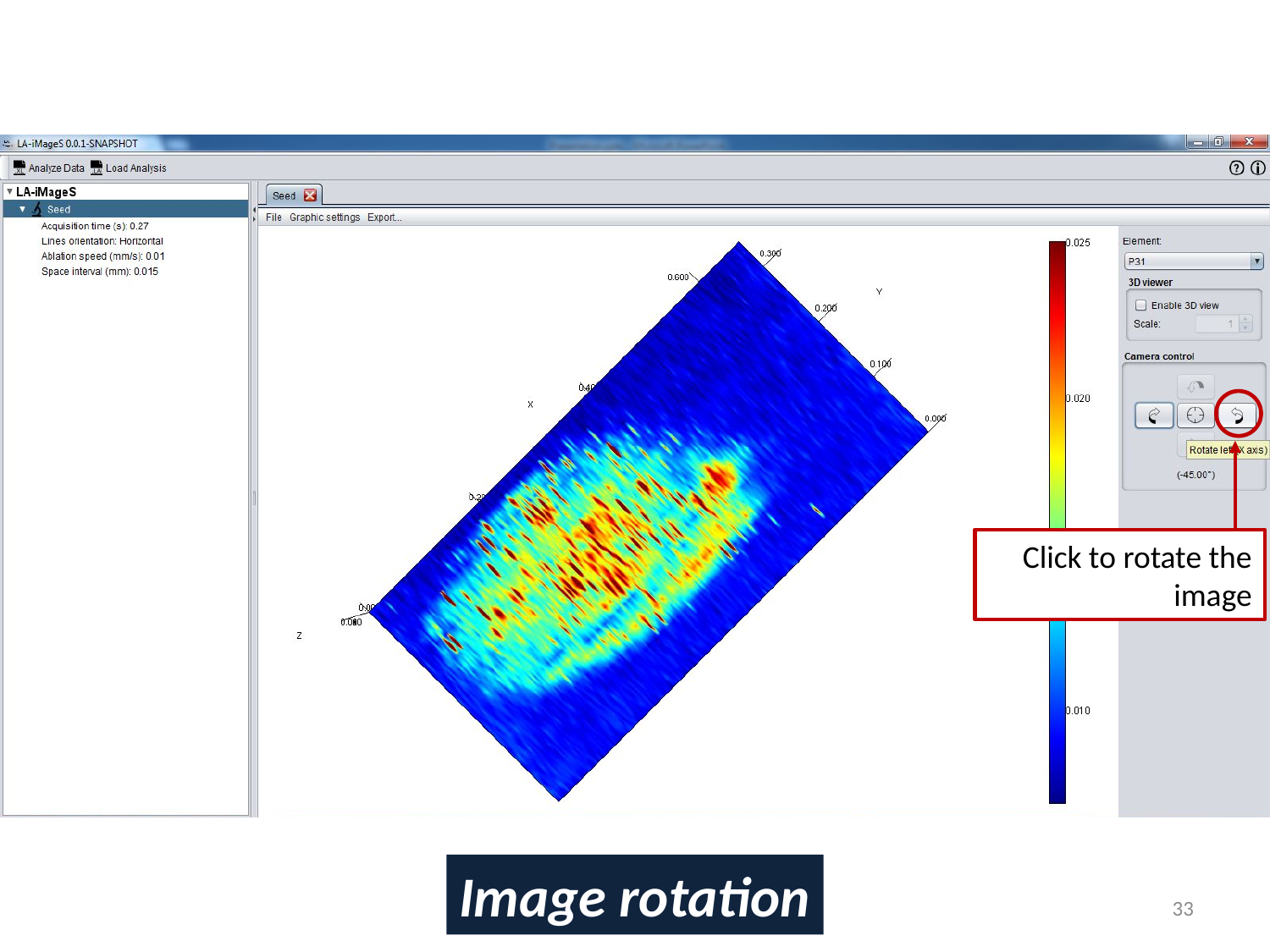

Click to rotate the image
Image rotation
33

## Slide 34
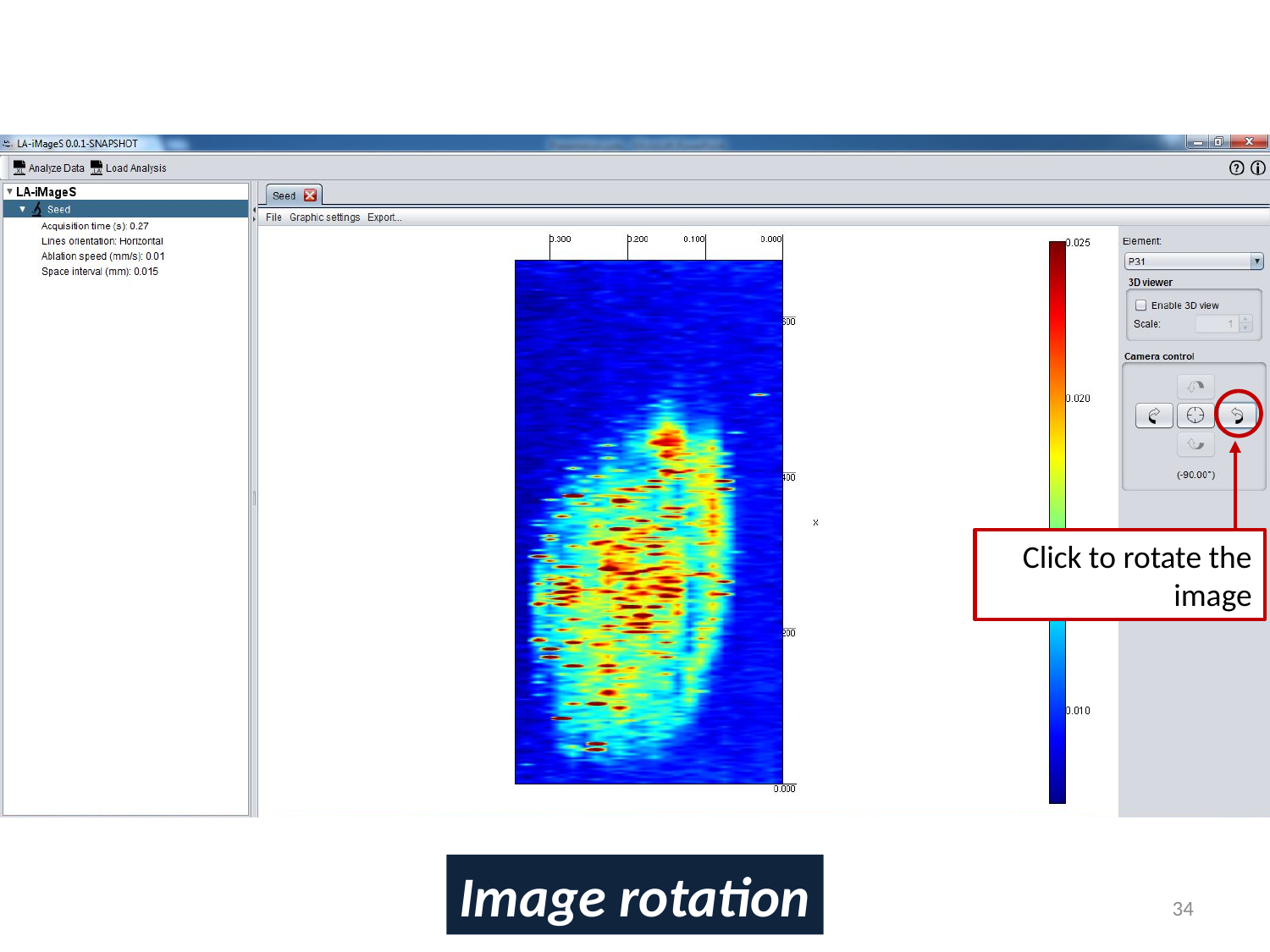

Click to rotate the image
Image rotation
34

## Slide 35
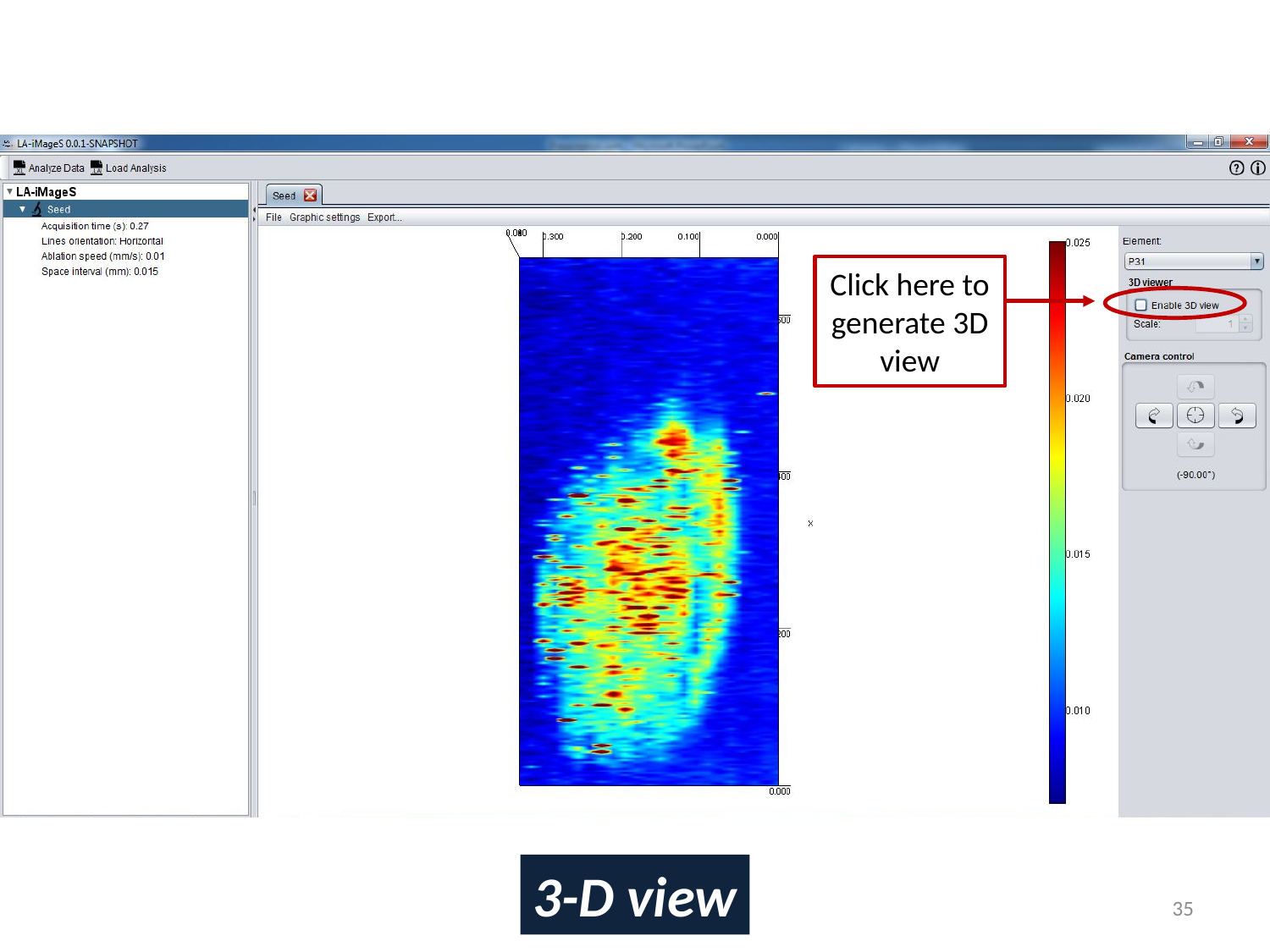

Click here to generate 3D view
3-D view
35

## Slide 36
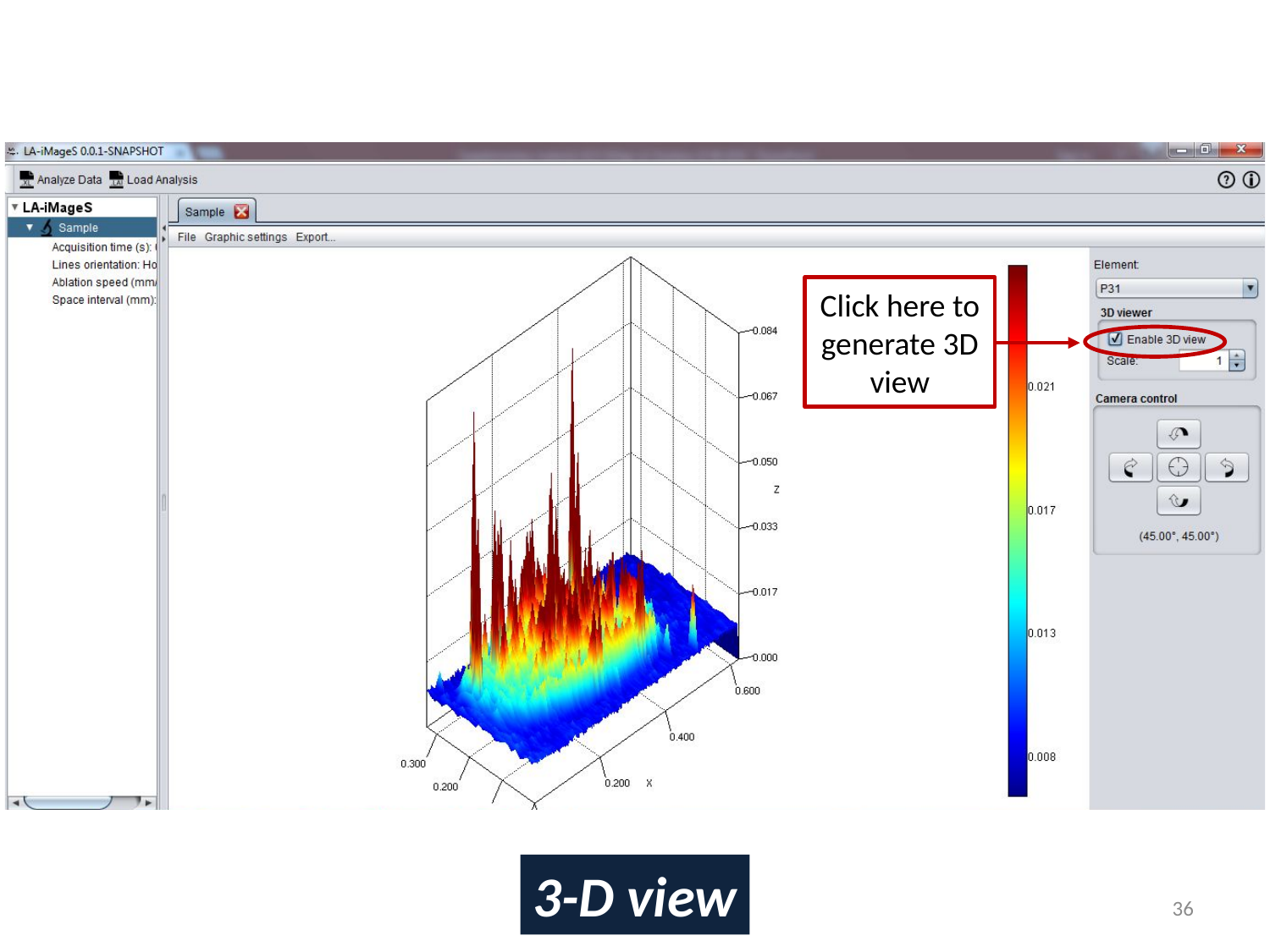

Click here to generate 3D view
3-D view
36

## Slide 37
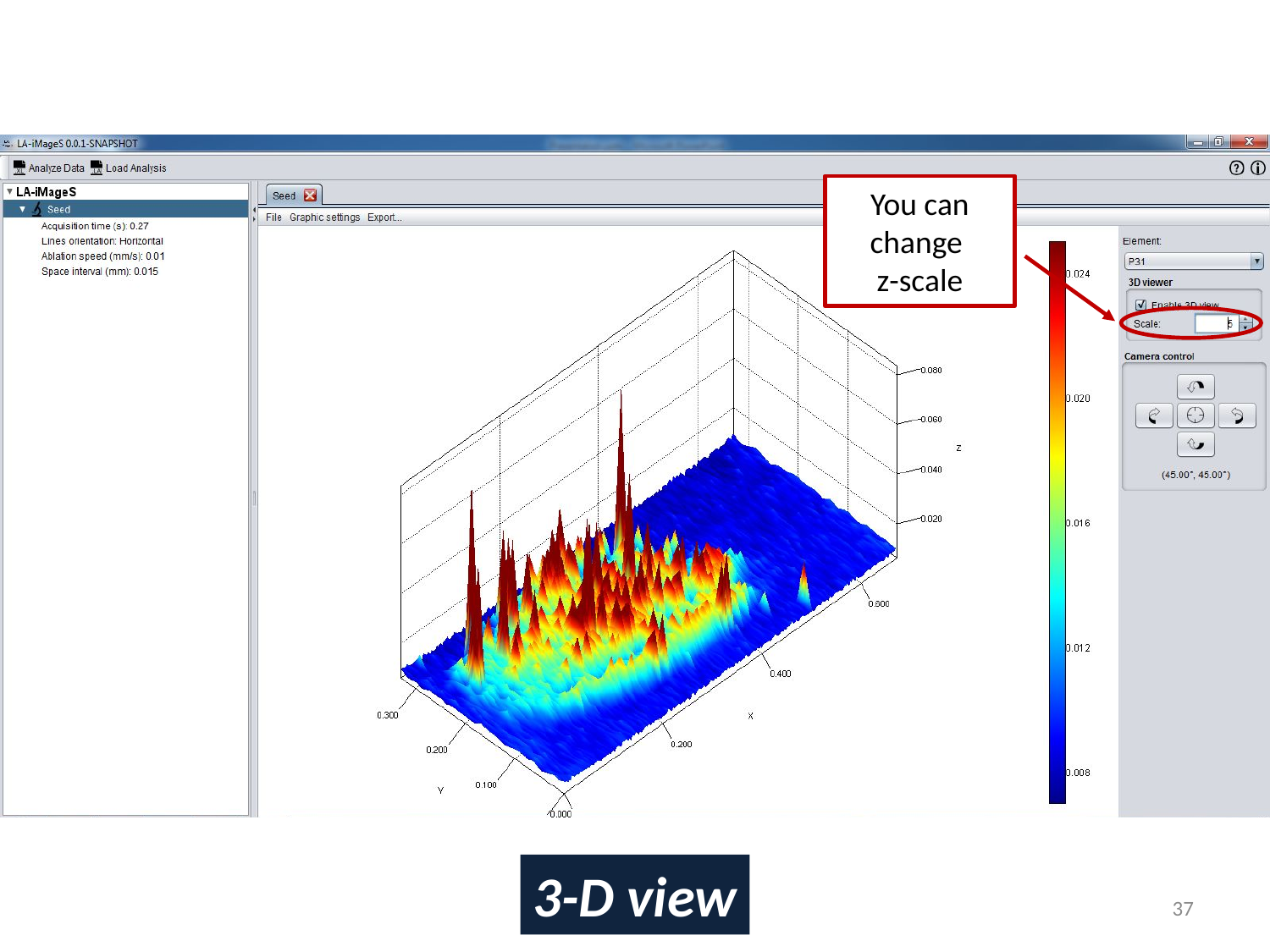

You can change
z-scale
3-D view
37

## Slide 38
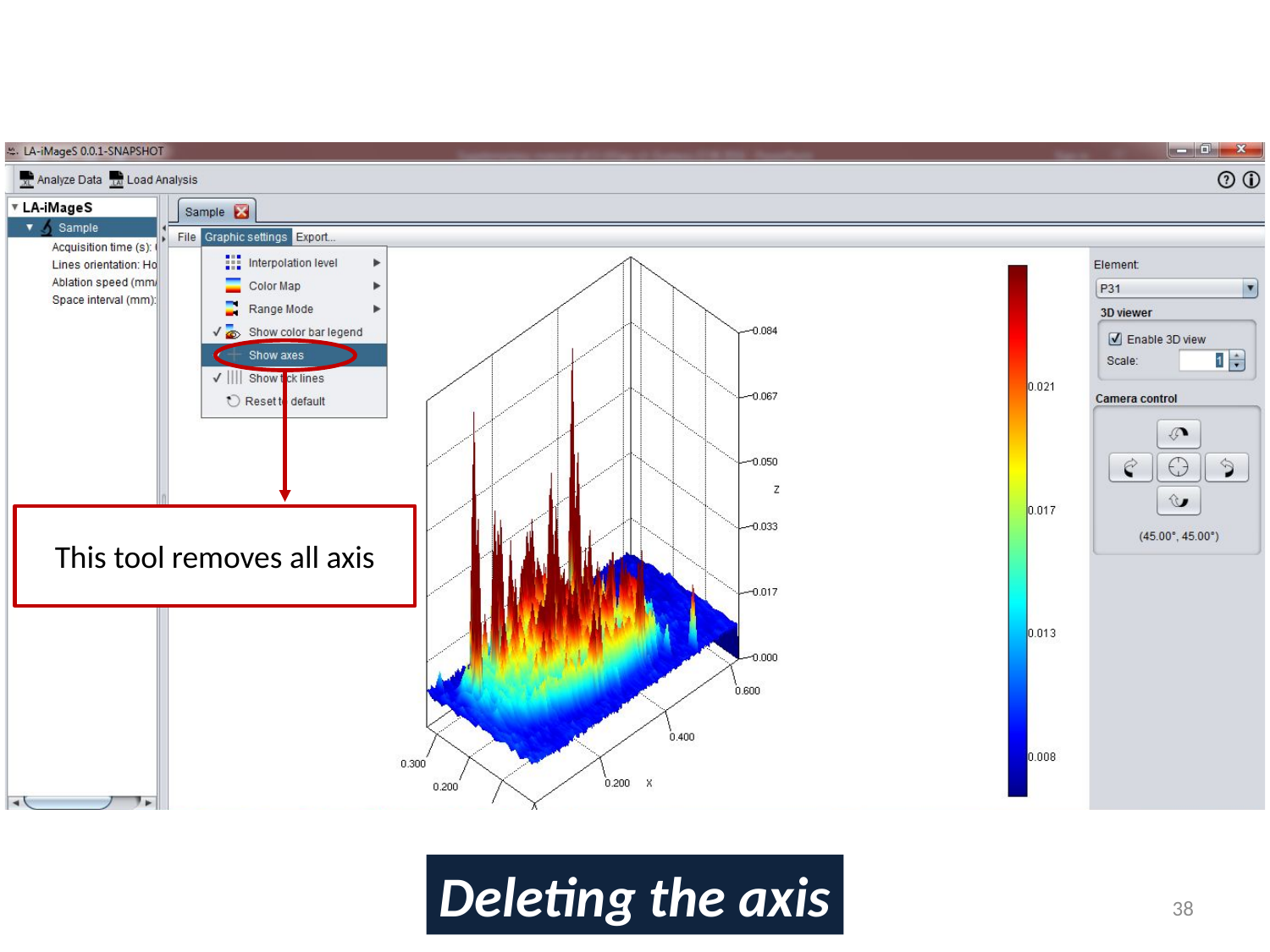

This tool removes all axis
Deleting the axis
38

## Slide 39
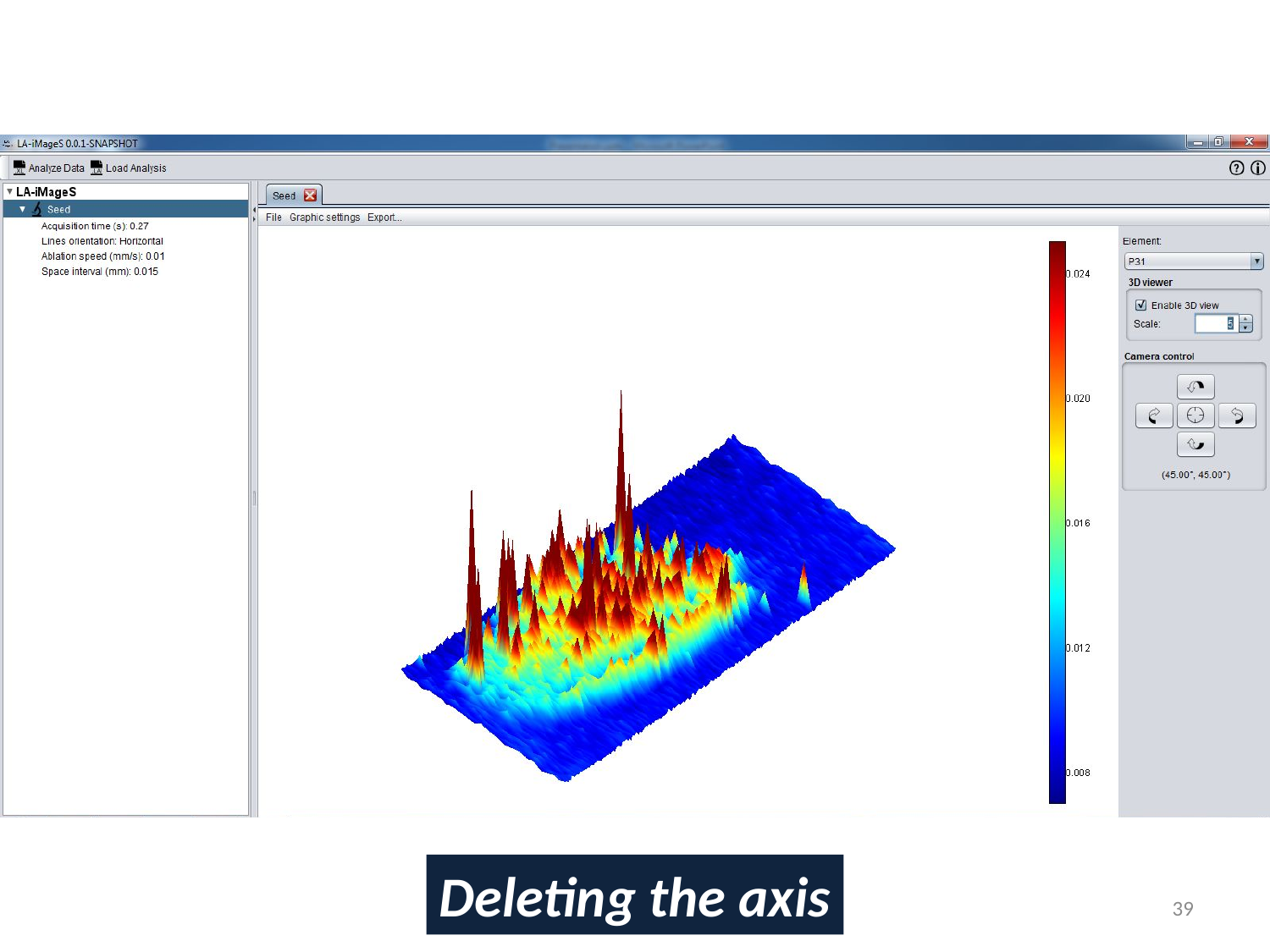

Deleting the axis
39

## Slide 40
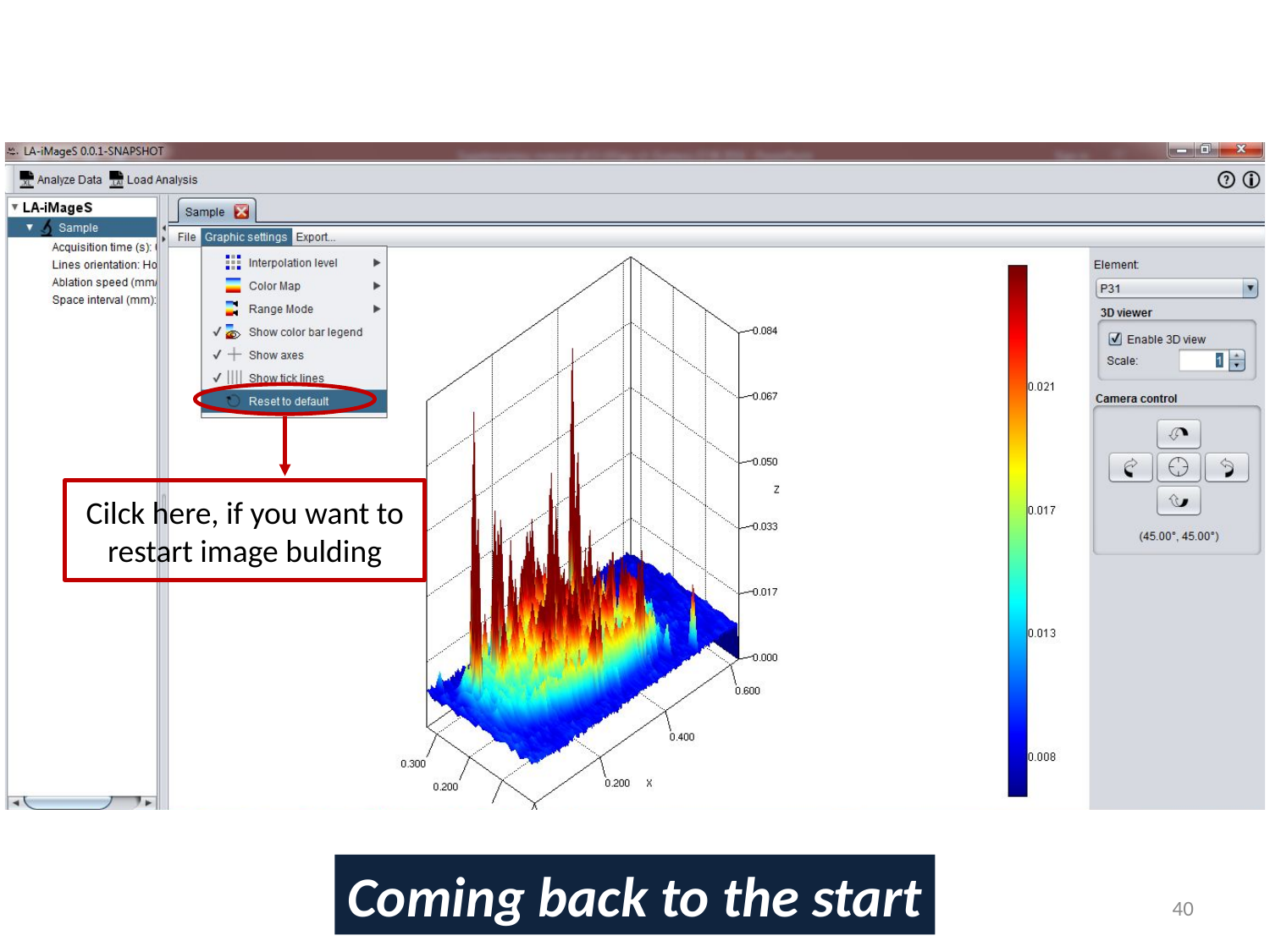

Cilck here, if you want to restart image bulding
Coming back to the start
40

## Slide 41
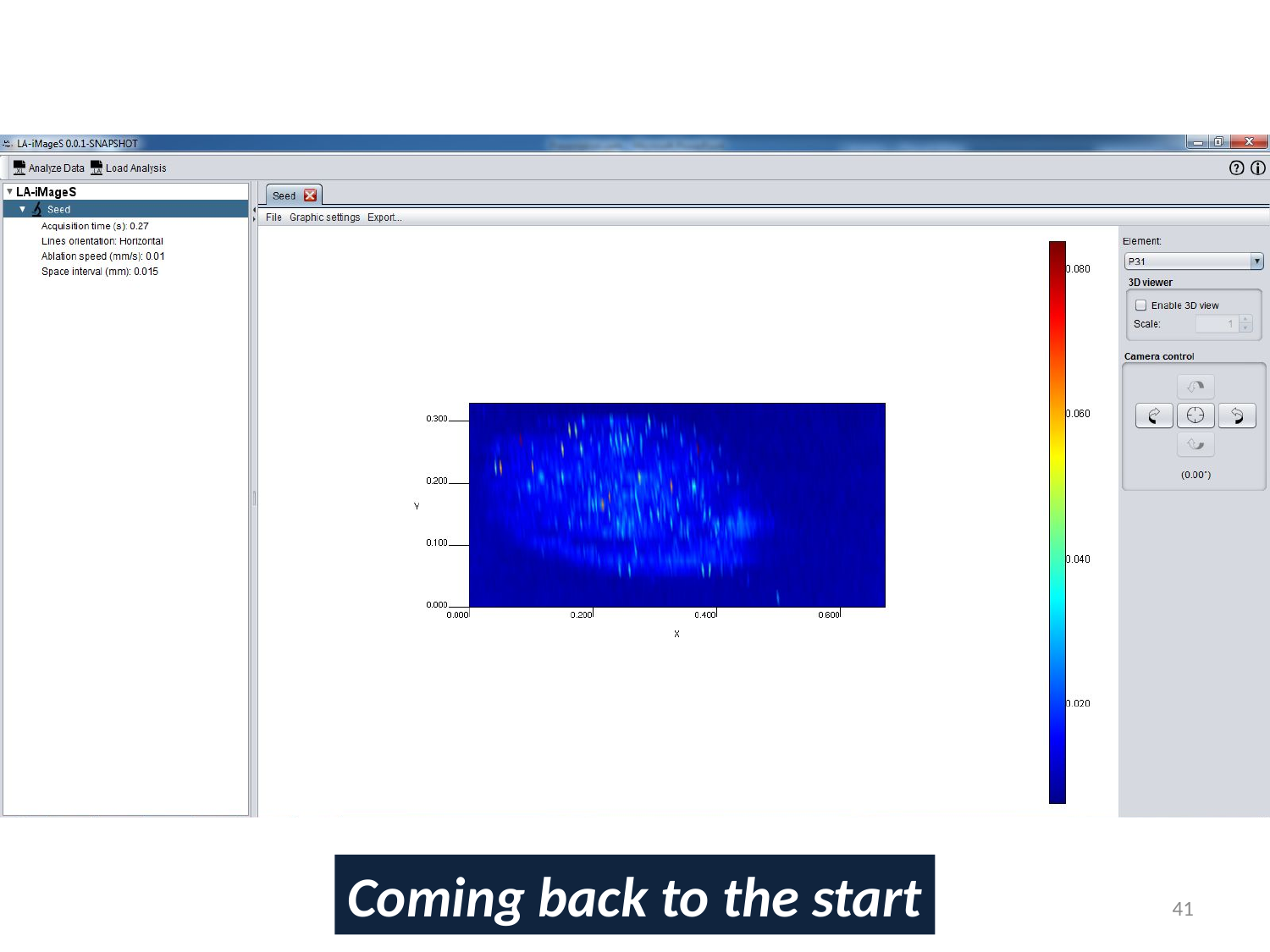

Coming back to the start
41

## Slide 42
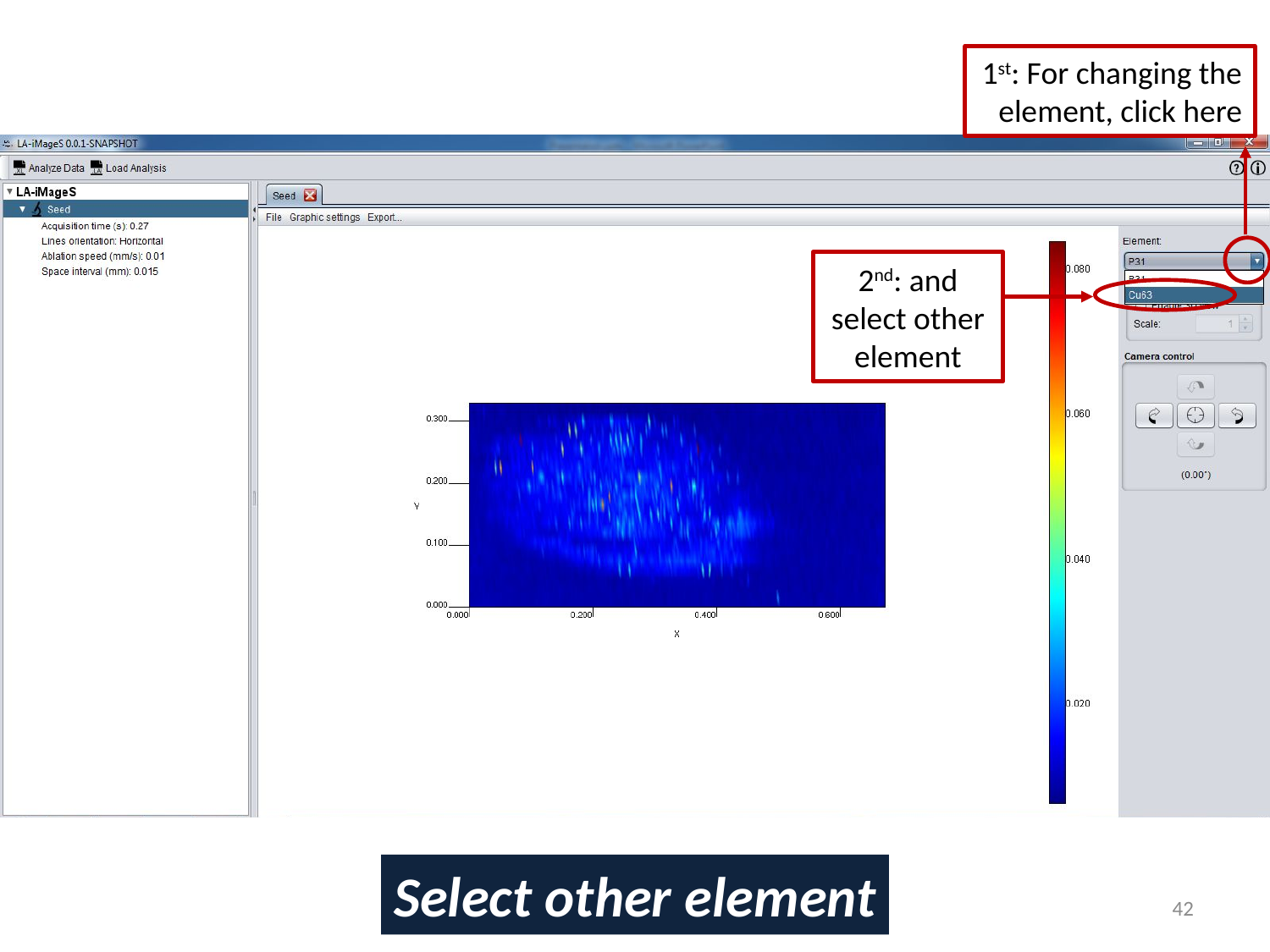

1st: For changing the element, click here
2nd: and select other element
Select other element
42

## Slide 43
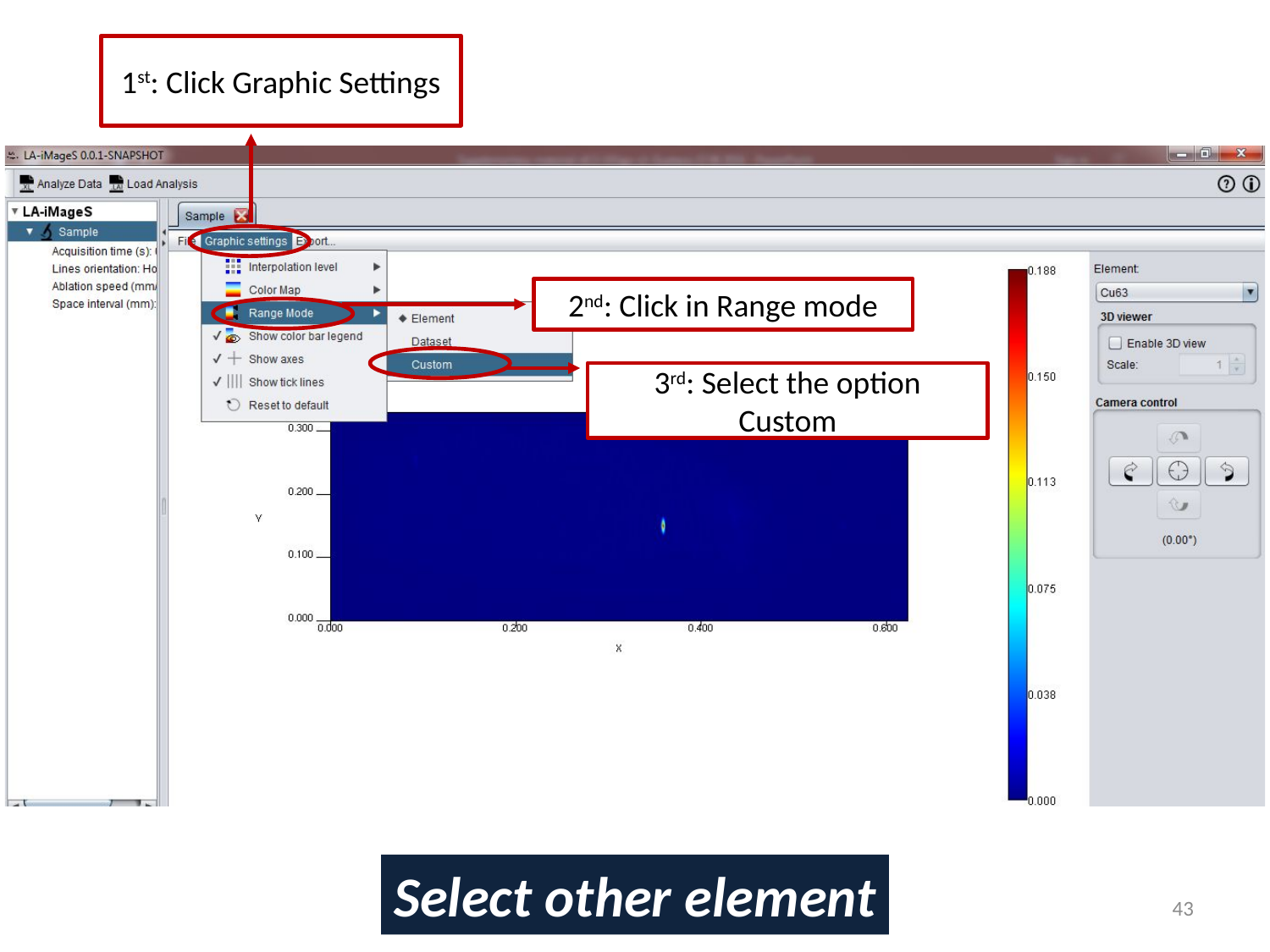

1st: Click Graphic Settings
2nd: Click in Range mode
3rd: Select the option
Custom
Select other element
43

## Slide 44
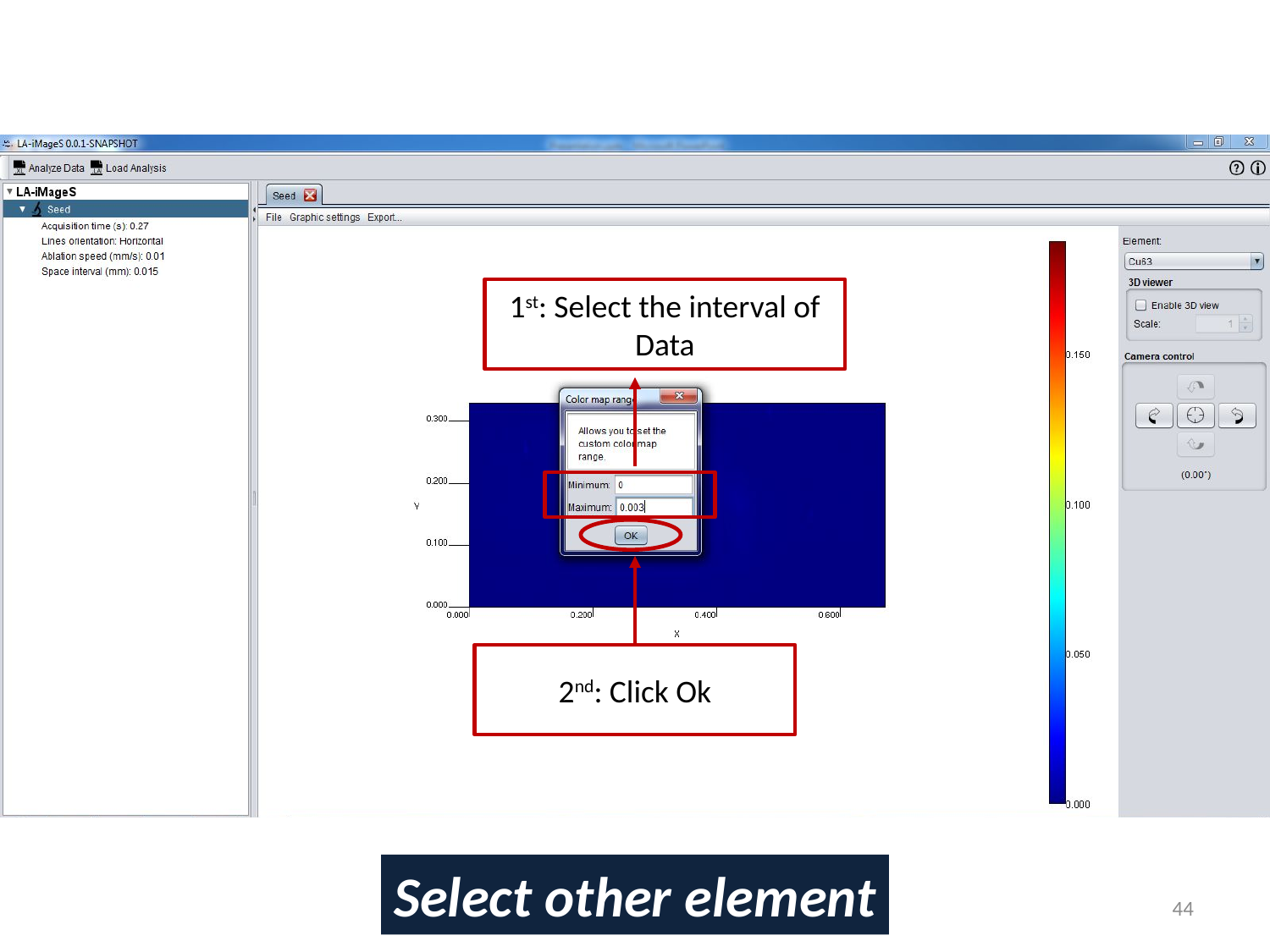

1st: Select the interval of Data
2nd: Click Ok
Select other element
44

## Slide 45
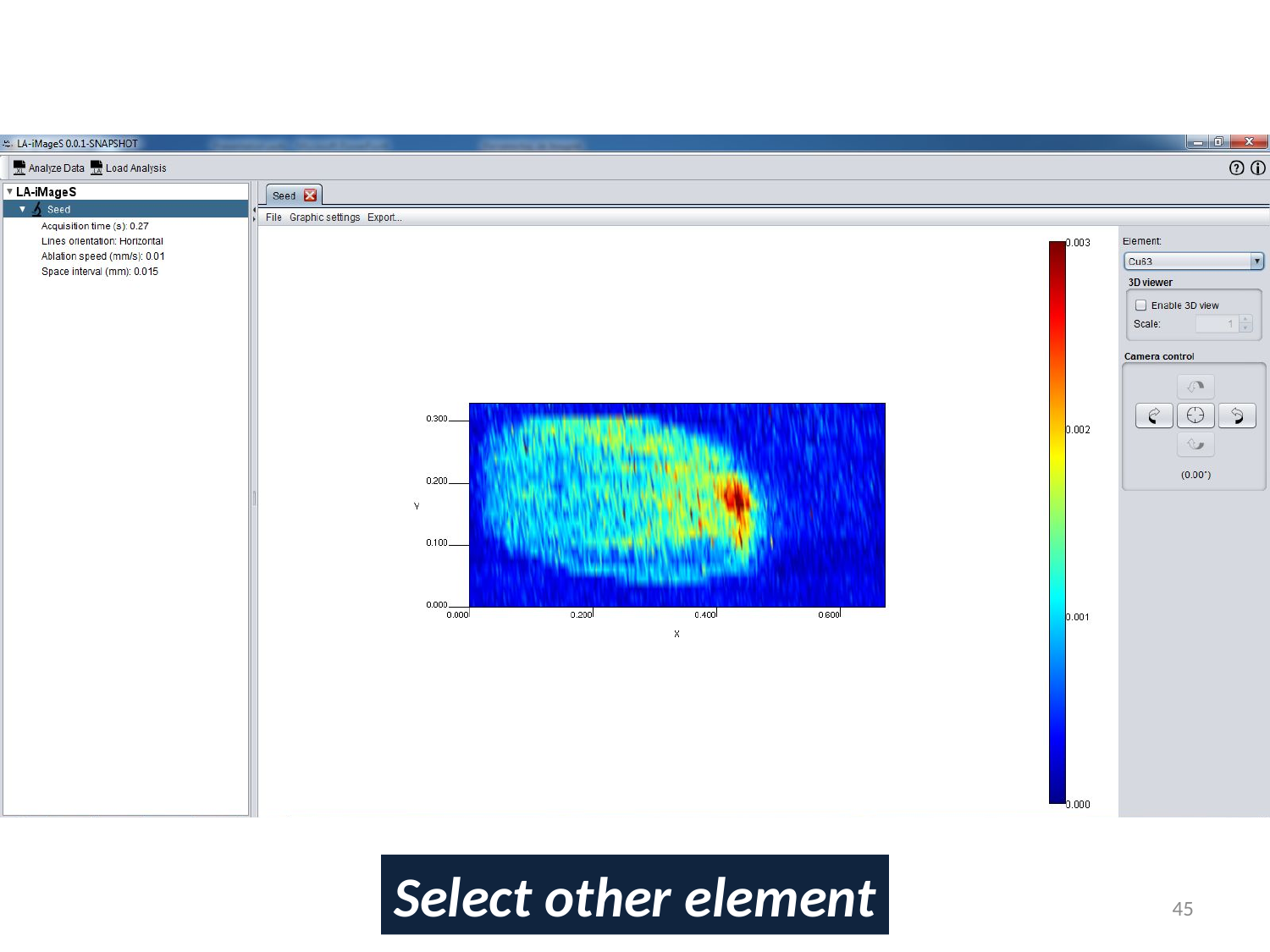

Select other element
45

## Slide 46
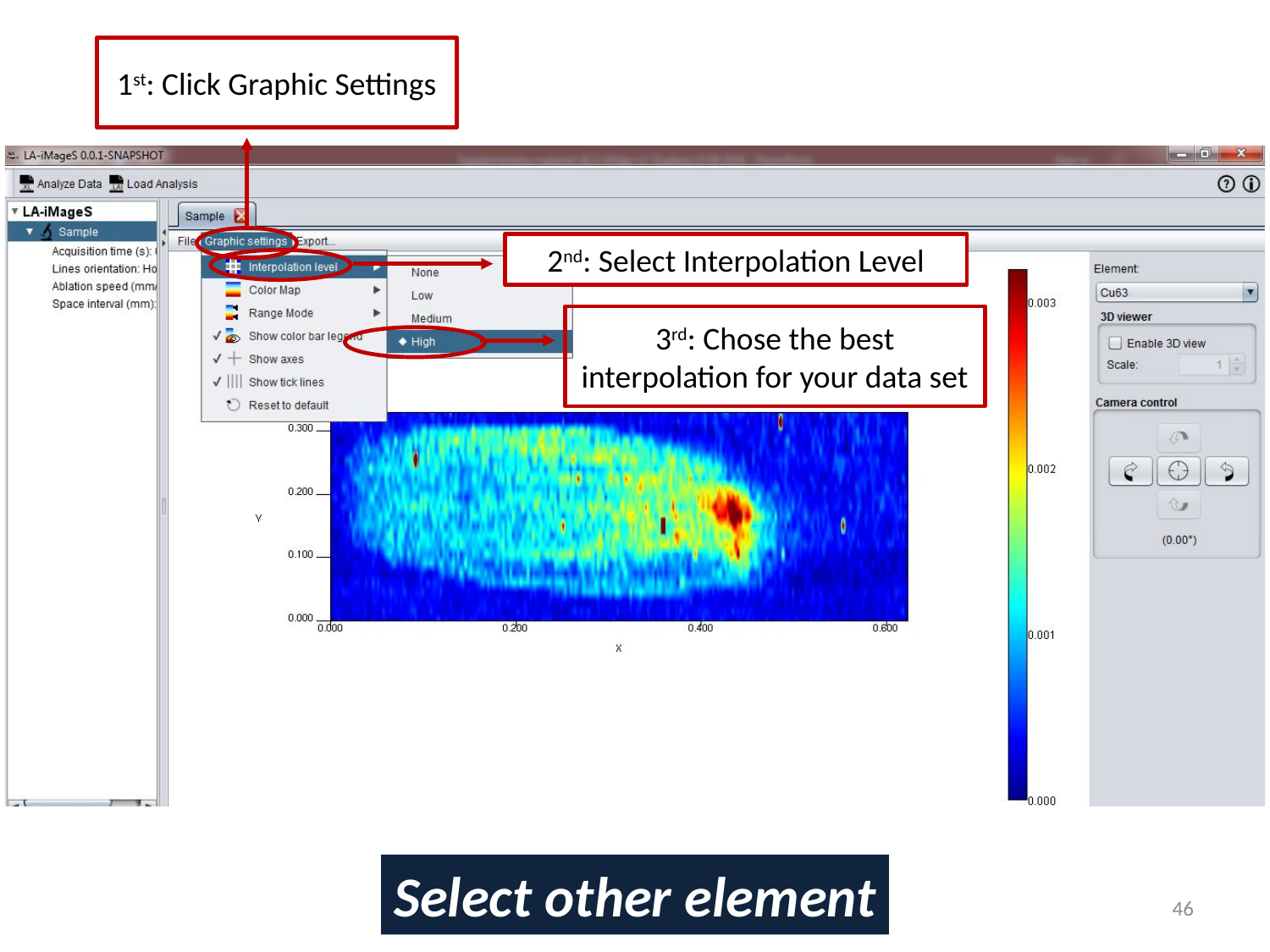

1st: Click Graphic Settings
2nd: Select Interpolation Level
3rd: Chose the best interpolation for your data set
Select other element
46

## Slide 47
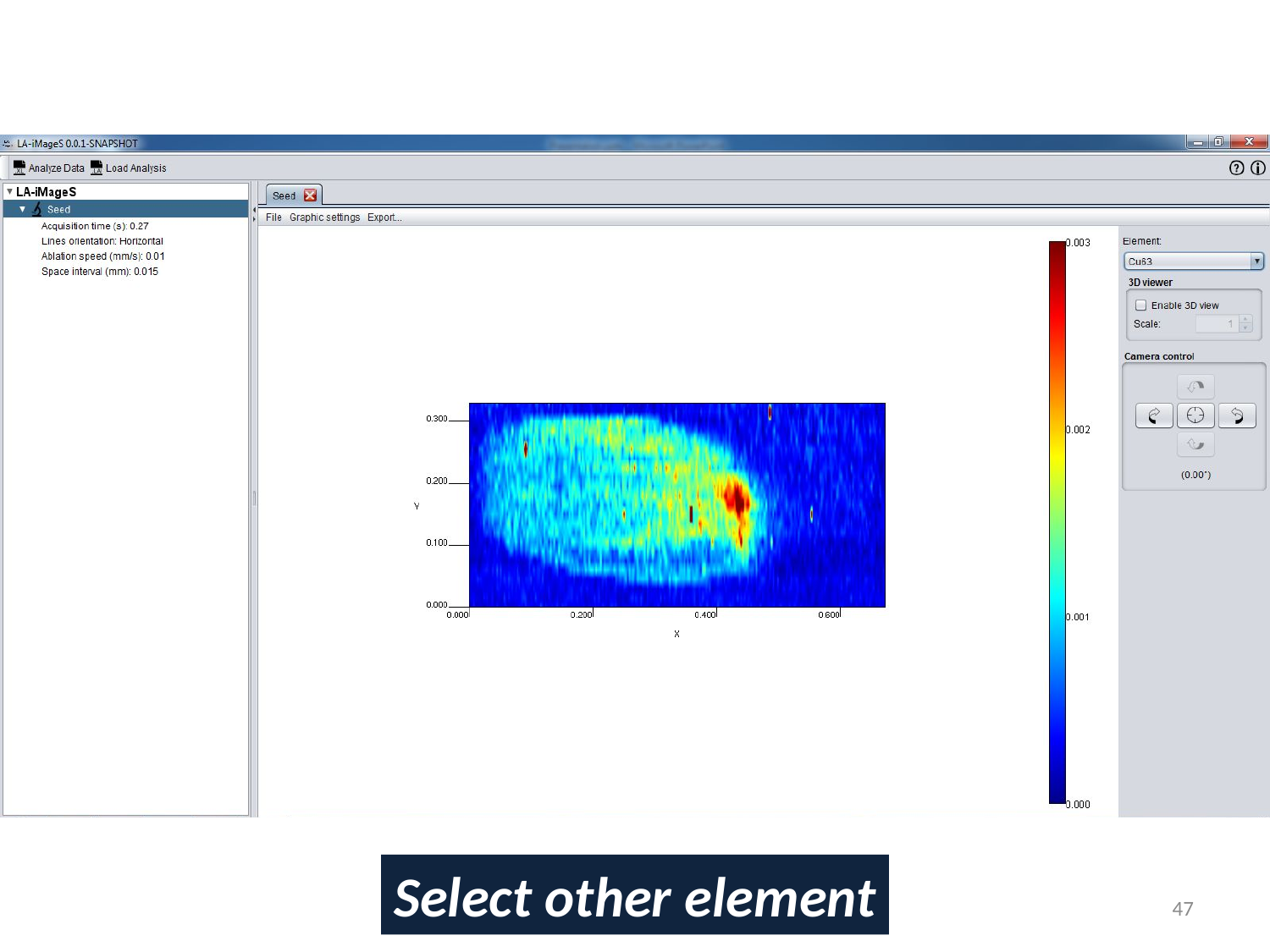

Select other element
47

## Slide 48
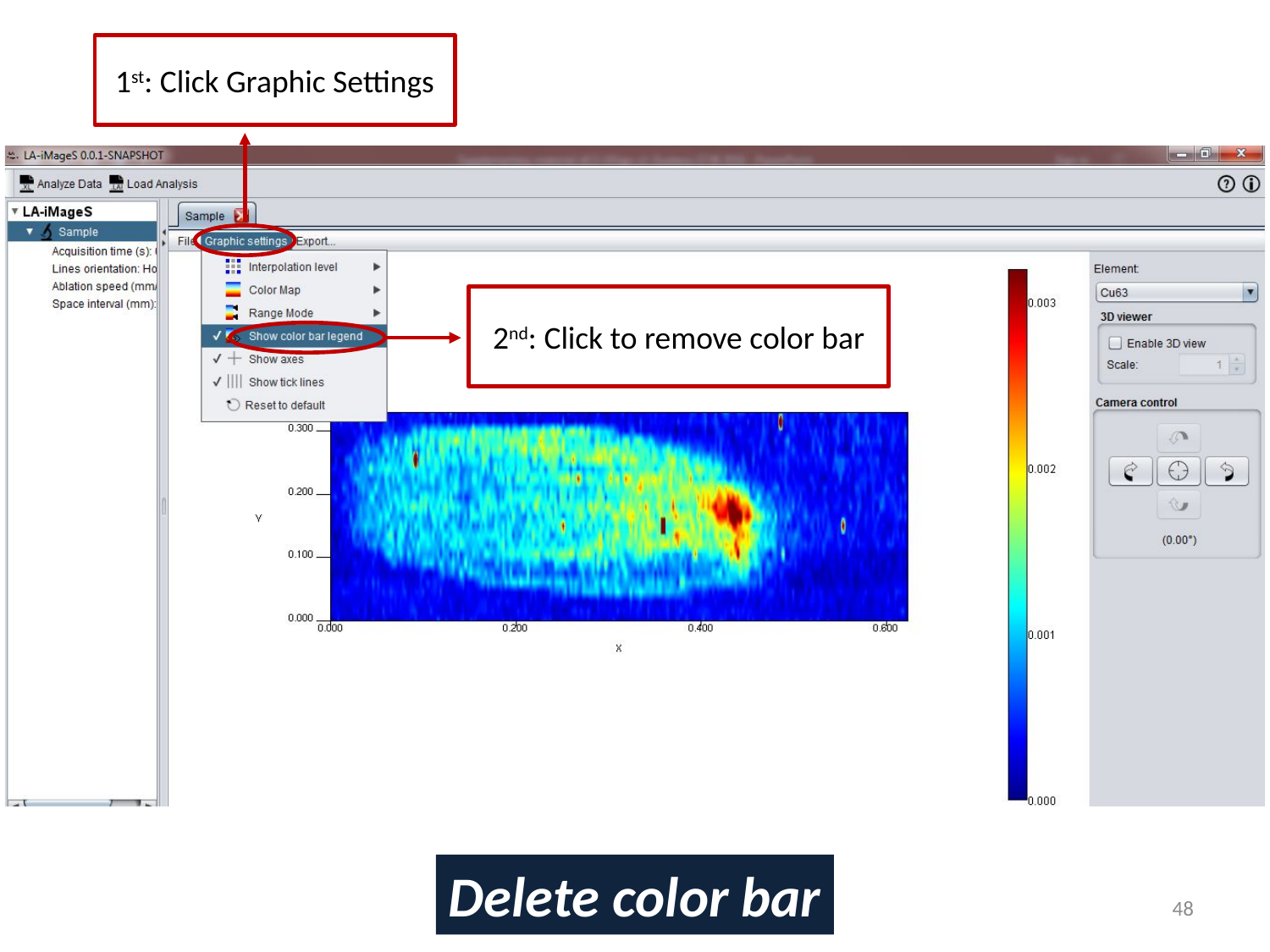

1st: Click Graphic Settings
2nd: Click to remove color bar
Delete color bar
48

## Slide 49
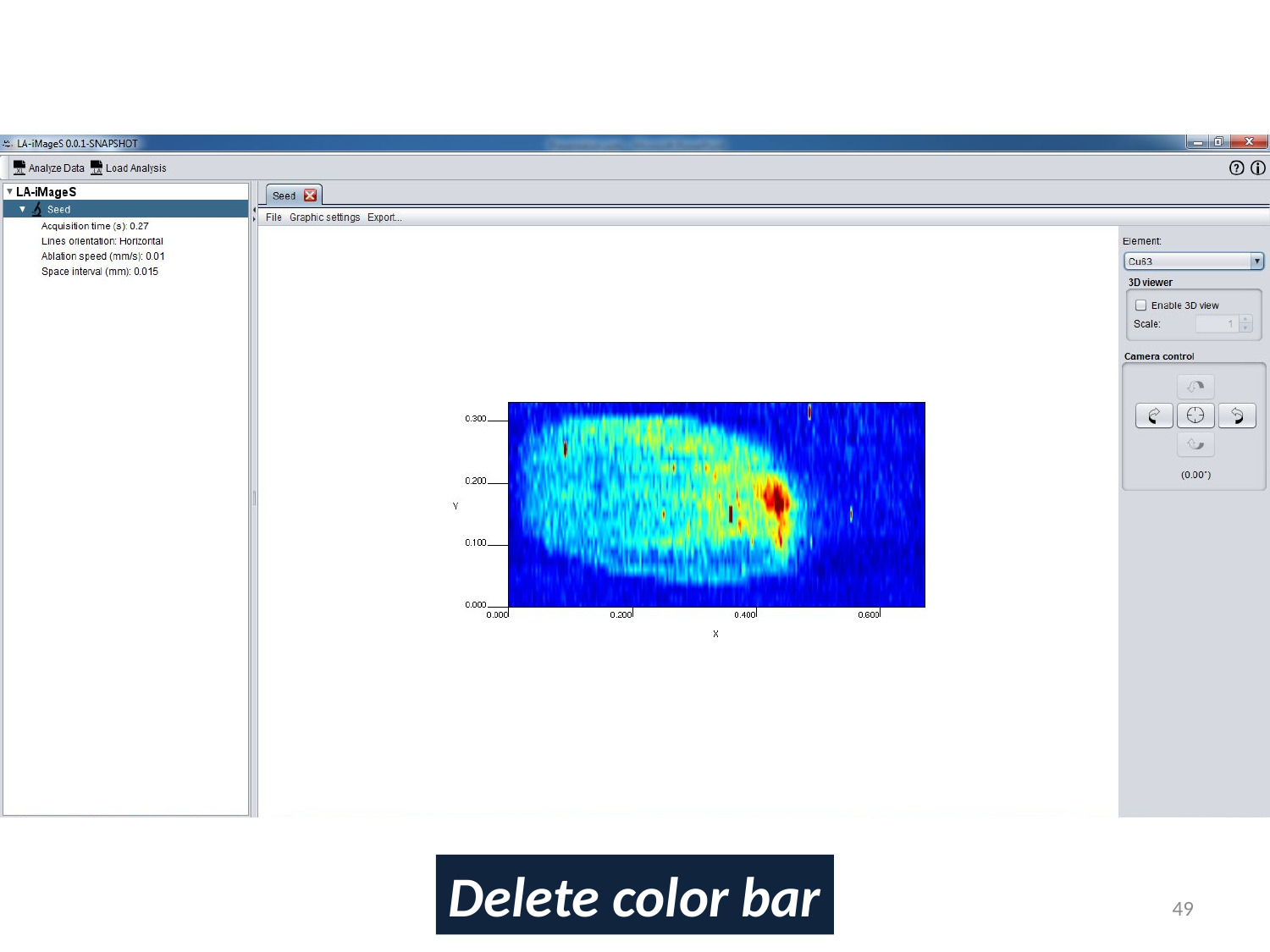

Delete color bar
49

## Slide 50
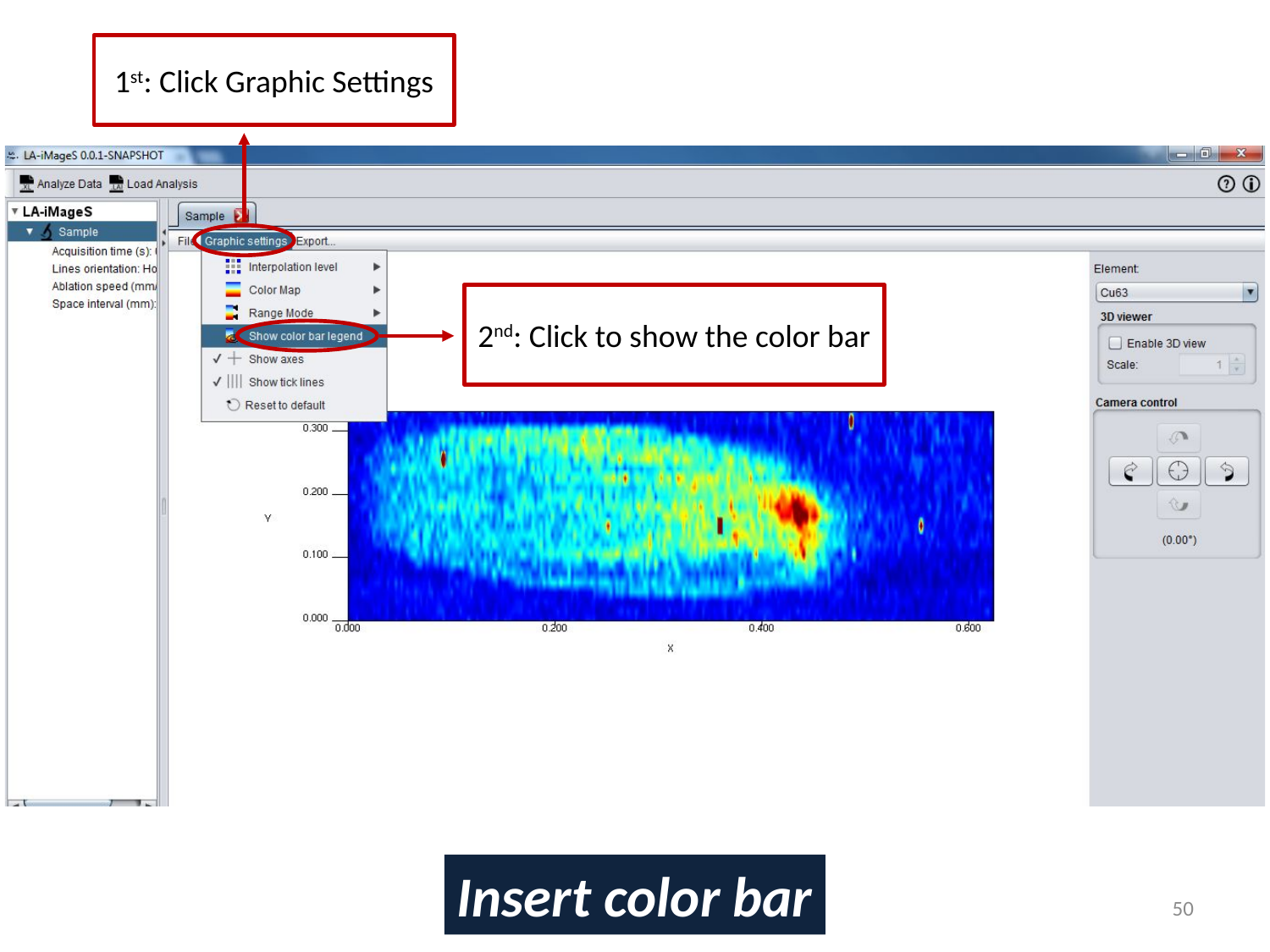

1st: Click Graphic Settings
2nd: Click to show the color bar
Insert color bar
50

## Slide 51
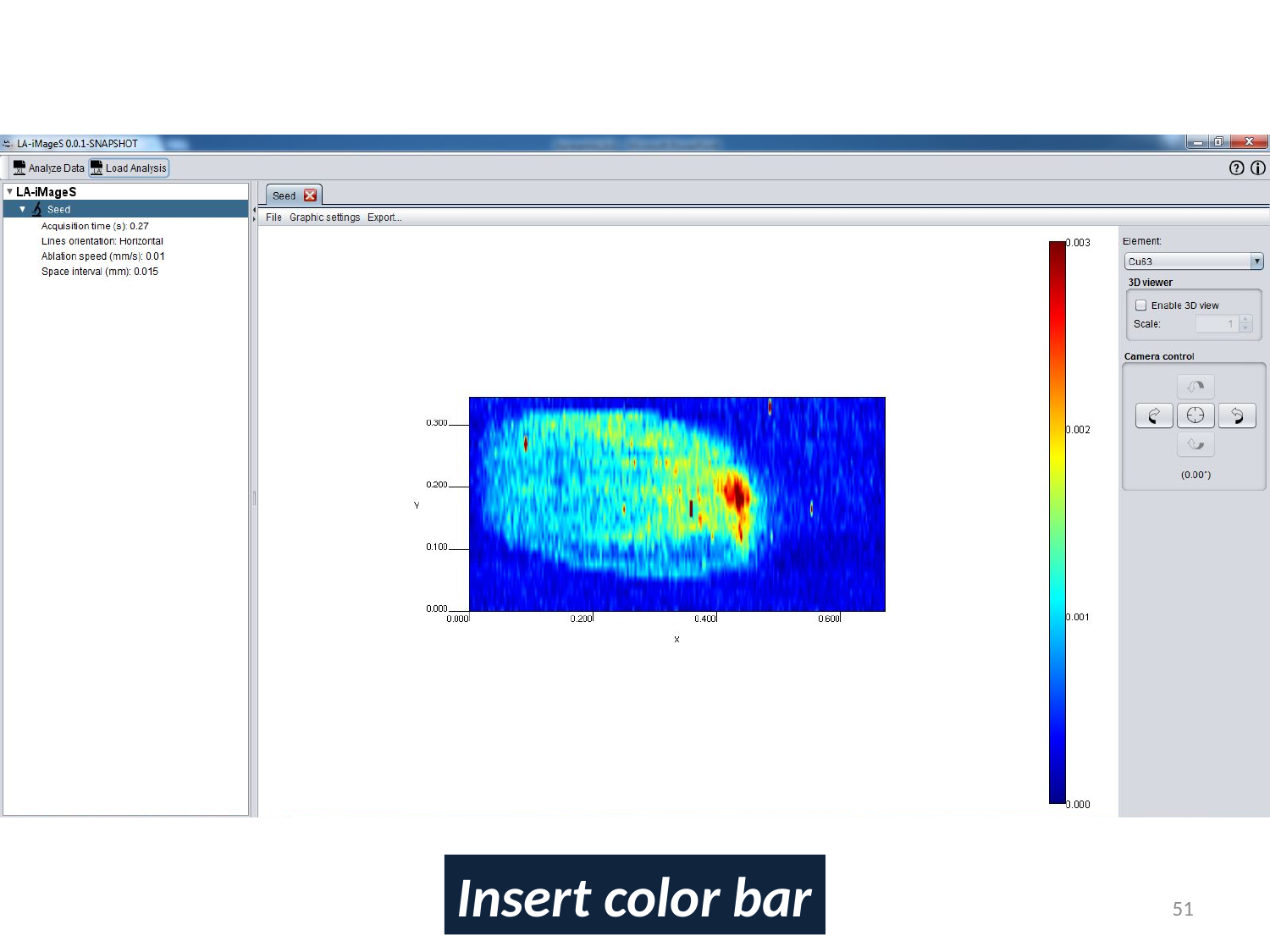

Insert color bar
51

## Slide 52
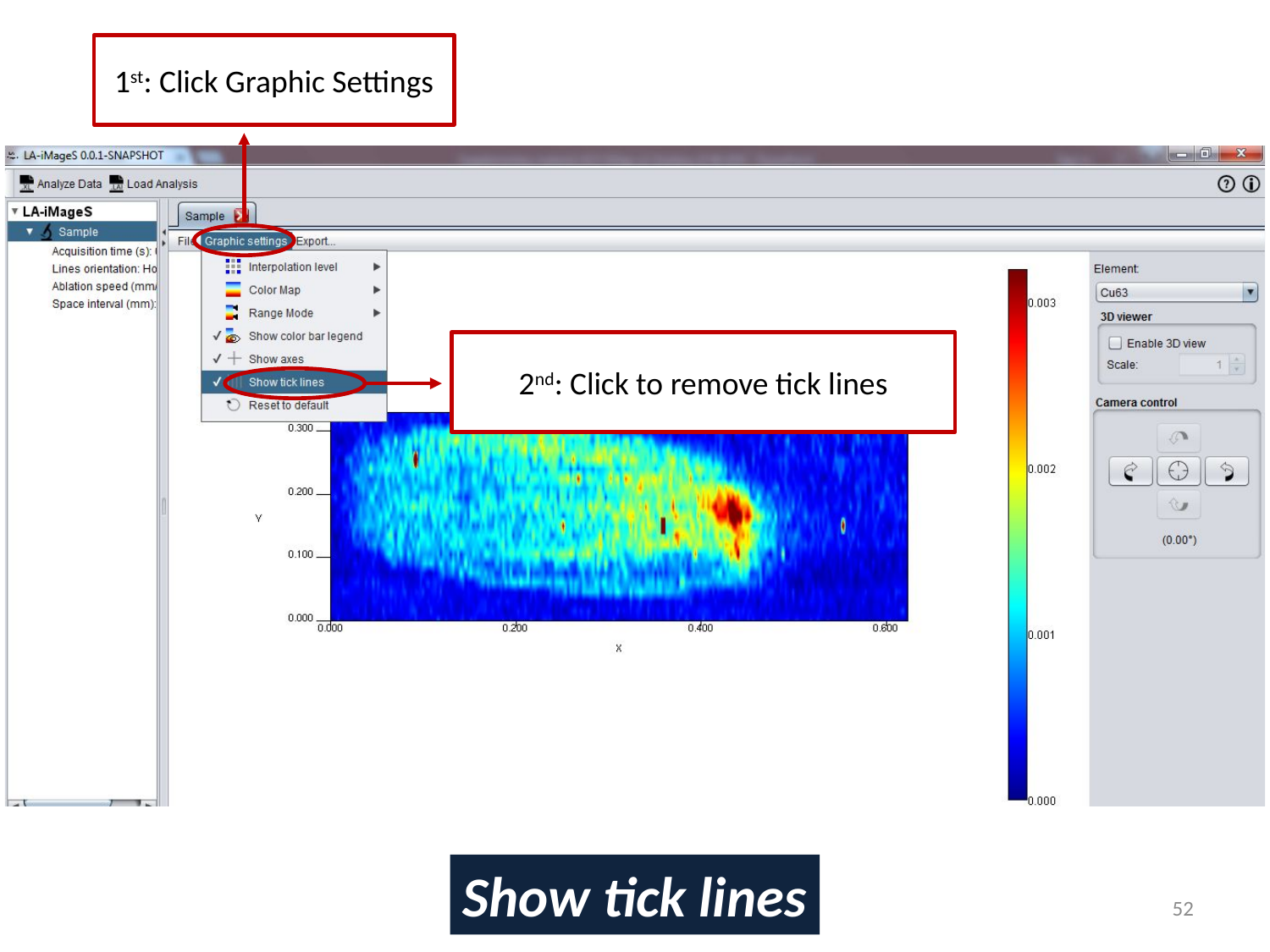

1st: Click Graphic Settings
2nd: Click to remove tick lines
Show tick lines
52

## Slide 53
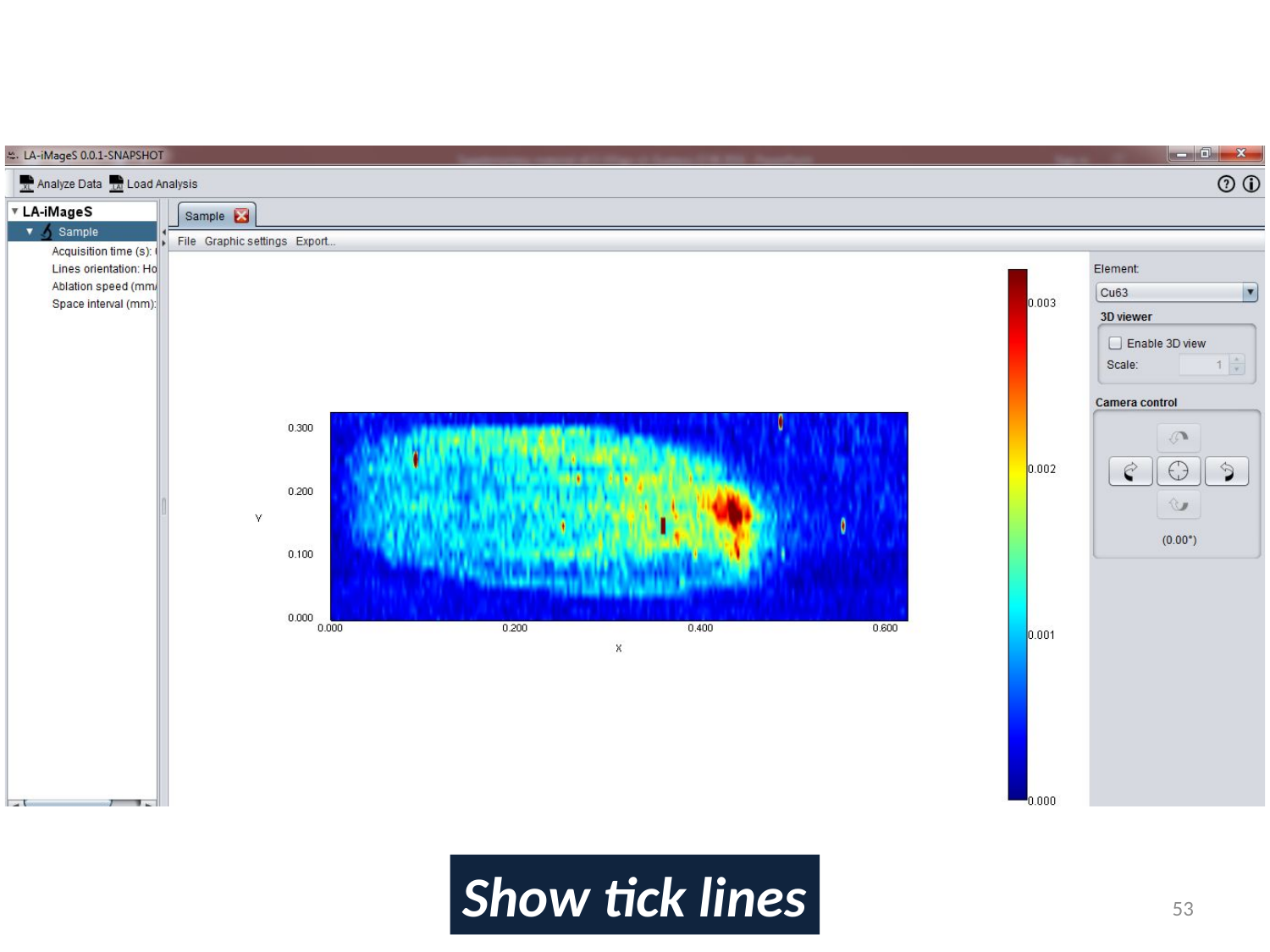

Show tick lines
53

## Slide 54
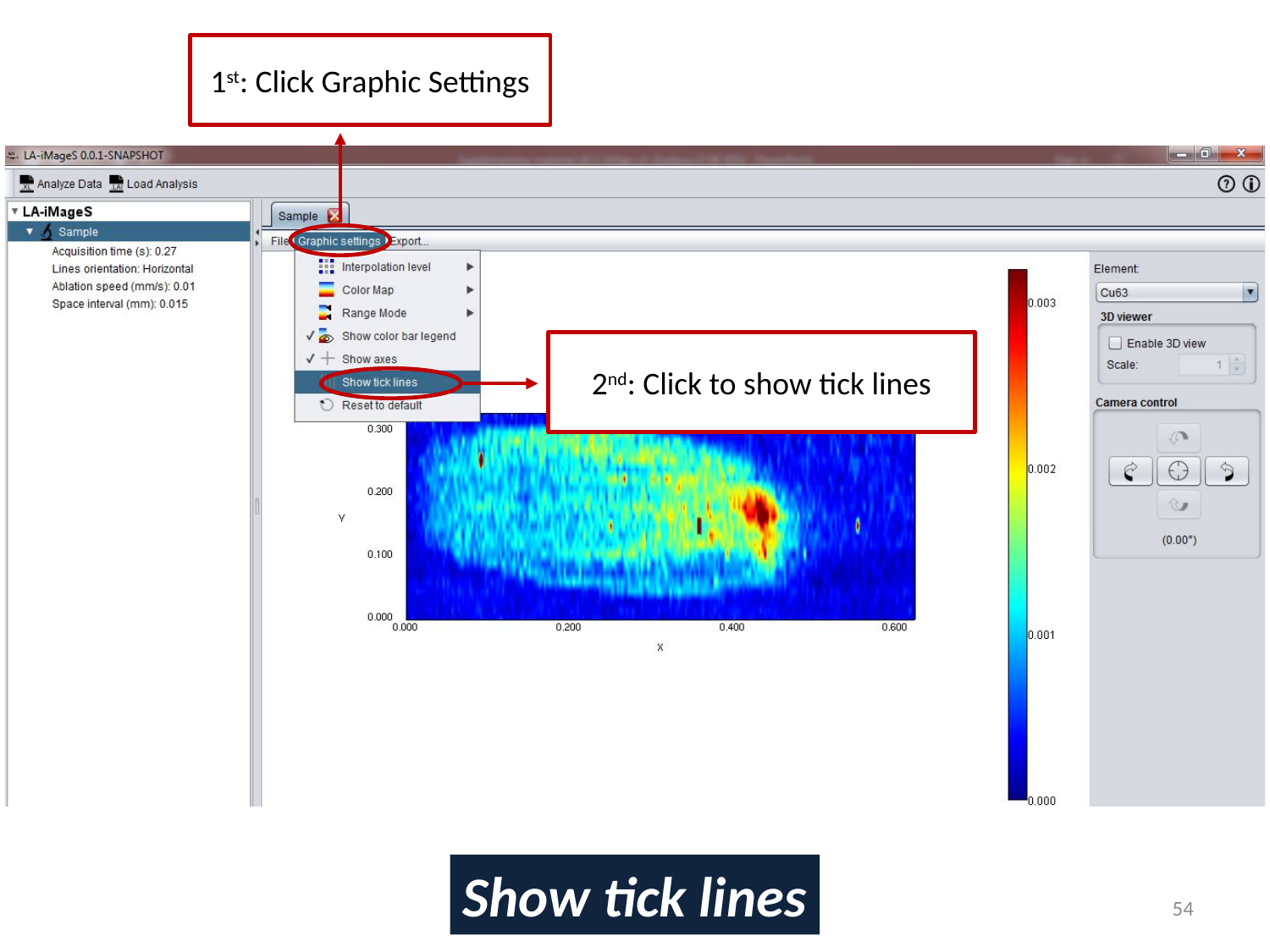

1st: Click Graphic Settings
2nd: Click to show tick lines
Show tick lines
54

## Slide 55
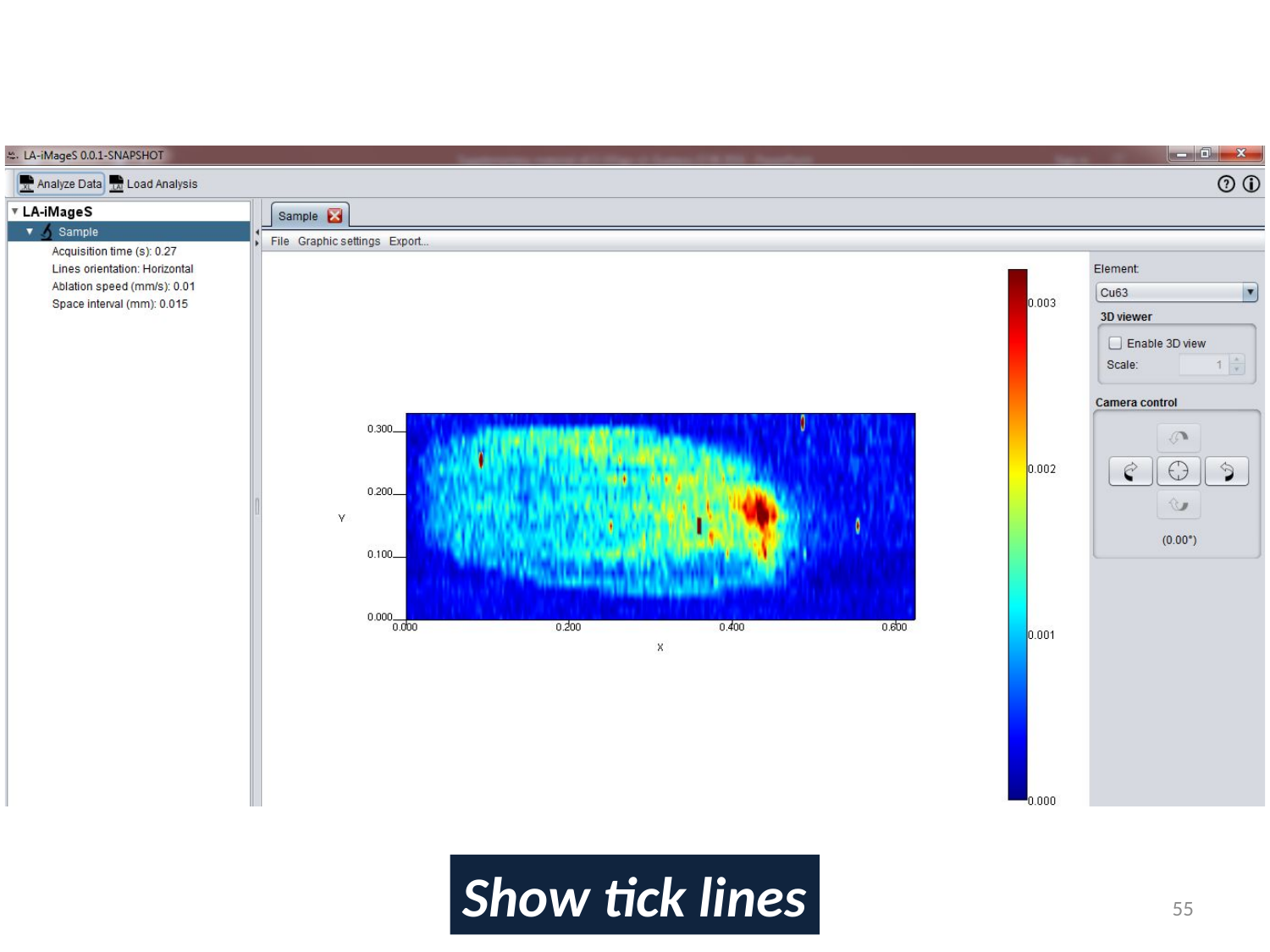

Show tick lines
55
